# A T-cell antigen atlas for meningioma: novel options for immunotherapy

**DOI:** 10.1007/s00401-023-02605-w

**Published:** 2023-06-27

**Authors:** Gioele Medici, Lena K. Freudenmann, Julia Velz, Sophie Shih-Yüng Wang, Konstantina Kapolou, Nagarajan Paramasivam, Lena Mühlenbruch, Daniel J. Kowalewski, Flavio Vasella, Tatjana Bilich, Beat M. Frey, Marissa L. Dubbelaar, Angelica Brooke Patterson, Anna Maria Zeitlberger, Manuela Silginer, Patrick Roth, Tobias Weiss, Hans-Georg Wirsching, Niklaus Krayenbühl, Oliver Bozinov, Luca Regli, Hans-Georg Rammensee, Elisabeth Jane Rushing, Felix Sahm, Juliane S. Walz, Michael Weller, Marian C. Neidert

**Affiliations:** 1 Laboratory of Molecular Neuro-Oncology, Department of Neurology, Clinical Neuroscience Center, University Hospital and University of Zurich, Frauenklinikstrasse 26, 8091 Zurich, Switzerland; 2 Department of Neurosurgery, Clinical Neuroscience Center, University Hospital and University of Zurich, Frauenklinikstrasse 10, 8091 Zurich, Switzerland; 3 Institute for Cell Biology, Department of Immunology, University of Tübingen, Tübingen, Germany; 4 DKFZ Partner Site Tübingen, German Cancer Consortium (DKTK), Tübingen, Germany; 5 Cluster of Excellence iFIT (EXC2180) “Image-Guided and Functionally Instructed Tumor Therapies”, University of Tübingen, Tübingen, Germany; 6 Department of Neurosurgery and Neurotechnology, Eberhard Karls University Tübingen, Tübingen, Germany; 7 Roche Diagnostics International Ltd, Rotkreuz, Switzerland; 8 Computational Oncology Group, Molecular Precision Oncology Program, NCT Heidelberg and DKFZ, Heidelberg, Germany; 9 Department of Peptide-Based Immunotherapy, University and University Hospital Tübingen, Tübingen, Germany; 10 Clinical Collaboration Unit Translational Immunology, German Cancer Consortium (DKTK), Department of Internal Medicine, University Hospital Tübingen, Tübingen, Germany; 11 Institute for Immunity, Transplantation and Infection, Stanford University School of Medicine, Stanford, CA USA; 12 Blood Transfusion Service, Swiss Red Cross, Schlieren, Switzerland; 13 Quantitative Biology Center (QBiC), Eberhard Karls University Tübingen, 72076 Tübingen, Baden-Württemberg Germany; 14 Institute of Immunobiology, Cantonal Hospital St.Gallen, 9007 St. Gallen, Switzerland; 15 Department of Neurosurgery, Cantonal Hospital St. Gallen, Rorschacher Strasse 95, 9007 St. Gallen, Switzerland; 16 Department of Neuropathology, University Hospital and University of Zurich, Schmelzbergstrasse 12, 8091 Zurich, Switzerland; 17 Department of Neuropathology, Heidelberg University Hospital, Heidelberg, Germany; 18 CCU Neuropathology, German Consortium for Translational Cancer Research (DKTK), German Cancer Research Center (DKFZ), Heidelberg, Germany

**Keywords:** Meningioma, Immunotherapy, HLA ligand, T-cell immunotherapy, Tumor-associated antigen

## Abstract

Meningiomas are the most common primary intracranial tumors. Although most symptomatic cases can be managed by surgery and/or radiotherapy, a relevant number of patients experience an unfavorable clinical course and additional treatment options are needed. As meningiomas are often perfused by dural branches of the external carotid artery, which is located outside the blood–brain barrier, they might be an accessible target for immunotherapy. However, the landscape of naturally presented tumor antigens in meningioma is unknown. We here provide a T-cell antigen atlas for meningioma by in-depth profiling of the naturally presented immunopeptidome using LC–MS/MS. Candidate target antigens were selected based on a comparative approach using an extensive immunopeptidome data set of normal tissues. Meningioma-exclusive antigens for HLA class I and II are described here for the first time. Top-ranking targets were further functionally characterized by showing their immunogenicity through in vitro T-cell priming assays. Thus, we provide an atlas of meningioma T-cell antigens which will be publicly available for further research. In addition, we have identified novel actionable targets that warrant further investigation as an immunotherapy option for meningioma.

**Supplementary Information:**

The online version contains supplementary material available at 10.1007/s00401-023-02605-w.

## Introduction

Meningiomas are the most common intracranial tumors [30] and the World Health Organization (WHO) classification distinguishes three grades of malignancy [24]. The mainstay of treatment for symptomatic tumors is surgery with radiotherapy reserved for selected cases with unfavorable clinical behavior or higher grade histological and molecular features [15]. Despite recent advances in the molecular understanding of meningiomas and the advent of modern diagnostic methods, such as next-generation sequencing and DNA methylation profiling [36], alternative systemic therapy options remain experimental. Currently, these approaches have demonstrated only modest success, such that no standard of care has been defined. There remains an urgent need for effective treatment options for meningiomas that cannot be controlled by surgery and radiotherapy.

Given the efficacy and safety of T-cell immunotherapy in other tumor entities, immunotherapy may also be a promising approach for meningiomas. Furthermore, meningiomas are supplied by vascular branches of the external carotid artery, which is not subject to the blood–brain barrier and thus accessible to immune cell infiltration [9, 25]. However, the landscape of actionable T-cell antigens as a foundation for antigen-specific immunotherapy is largely unknown in meningiomas.

In this study, we have mapped the Human Leukocyte Antigen (HLA) peptidome using immunoaffinity purification and mass spectrometry (LC–MS/MS) to provide a comprehensive atlas of naturally presented T-cell antigens. Meningioma-exclusive antigens are functionally characterized in T-cell immunogenicity tests and provide a set of actionable targets for T-cell-based immunotherapy.

## Materials and methods

### Patient cohort

Primary tissues of 33 patients with histologically confirmed meningioma (22 WHO grade I, 9 WHO grade II, and 2 WHO grade III) were collected and snap-frozen during surgery in the Department of Neurosurgery of the University Hospital Zurich. Frozen specimens were stored at − 80 °C until further use. Three samples were obtained during surgery at disease recurrence. Written informed consent was obtained for all patients in accordance with the Declaration of Helsinki and the local review board (Kantonale Ethikkommission Zürich; KEK-ZH-Nr. 2015-0163). Autologous tumor-free dura supplementing the in-house benign HLA peptidome dataset [26] was available for nine patients.

### Immunohistochemistry

Immunohistochemistry was carried out on paraformaldehyde-fixed paraffin-embedded meningioma sections. Sections were stained using the murine monoclonal primary antibody against HLA-A, -B, -C (1:50) (BD Pharmingen) or HLA class II (1:50) (Abcam). Anti-mouse with ImmPACT DAB kit was used as the secondary antibody (Vector, Burlingame, CA, USA). Images were acquired using a Zeiss Scope.A1 microscope (Carl Zeiss Microscopy, Jena, Germany).

### HLA typing

HLA class I and II allotypes of MNG499, MNG 501, MNG623, MNG624, MNG628, MNG641, MNG642, MNG646, MNG661, MNG666, MNG673, MNG679, MNG682, MNG700, MNG702, MNG734, MNG814, MNG819, and MNG833 were determined at a 4-digit level by next-generation sequencing of tumor tissue at HistoGenetics (New York, NY, USA). For the remaining patients, 4-digit HLA class I typing was obtained from whole-genome sequencing using the Polysolver algorithm [41]. A detailed description of the method used for whole-genome sequencing of meningioma and matched blood as the control can be retrieved from Paramasivam et al. [31].

### HLA ligand immunopurification

HLA-I and HLA-II molecules were isolated from snap-frozen tissue using standard immunoaffinity chromatography. CNBr-activated sepharose [GE Healthcare, Little Chalfont (UK)] was cross-linked with either pan-HLA-I-specific antibody W6/32 [4], HLA-DR-specific antibody L243 [39], or Tü39 [29] specific for HLA-DP, -DQ, -DR (all produced in-house) at a ratio of 40 mg per 1 mg of antibody with 0.5 M NaCl and 0.1 M NaHCO_3_ at pH 8.3. To precipitate HLA-peptide complexes, at least 1 mg antibody (W6/32 for HLA-I or Tü39 and L243 mixed 1:1 for HLA-II) was employed, which was increased to 1 mg per 1 g of tissue for samples > 1 g. Tissue homogenization was performed in a lysis buffer consisting of CHAPS (PanReac AppliChem, Darmstadt, Germany) and one cOmplete^™^ protease inhibitor cocktail tablet (Roche, Basel, Switzerland) in PBS. After mechanical disruption using a scalpel and a Potter–Elvehjem tissue homogenizer, samples were incubated for 60 min on a shaker at 4 °C. To further disrupt cell membranes, samples underwent five cycles of pulsed sonification, each lasting 20 s, followed by another 1 h incubation period on the shaker at 4 °C. Lysate was cleared by centrifugation for 1 h at 4000 rpm and 4 °C for 1 h (repeated once for the supernatant of tissues ≥ 1 g) and by sterile filtration employing 5.0 μm low protein-binding filter units (Merck Millipore). For each sample, two columns were mounted, one above the other, so that the flowthrough of the first, containing HLA class I-specific antibodies, dropped directly into the second column equipped with HLA class II-specific antibodies. The lysates were passed through the columns cyclically overnight at 4 °C. After washing the affinity columns for 30 min with PBS and for 1 h with water, peptide elution was performed by incubating four times successively with 100 µl 0.2% trifluoroacetic acid (TFA) per 1 mg antibody on a shaker. Peptides were separated from the HLA molecules by ultrafiltration using 3 kDa and 10 kDa Amicon filter units (Merck Millipore) for HLA-I and HLA-II, respectively. By lyophilization, the eluate volume was subsequently reduced to approximately 30 µl.

Finally, peptide solutions were desalted by reversed-phase liquid chromatography using ZipTip_c18_^®^ Pipette tips (Merck Millipore, 0.6 µl bed volume) and using 32.5% acetonitrile (ACN) with 0.2% TFA as eluent. The hydrophobic solvent content was removed by vacuum centrifugation, and 1% ACN with 0.05% TFA was added to a final volume of 25 µl. Samples were stored at -80 °C until LC–MS/MS analysis.

### LC–MS/MS and data analysis

The purified peptides were analyzed by nanoflow high-performance liquid chromatography (Dionex Ultimate^™^ 3000 Series liquid chromatography system) and tandem mass spectrometry. For each tissue and HLA class, a minimum of three technical replicates consuming 5 µl sample and lasting 130 min each were acquired. Peptide solutions were loaded with 0.1% formic acid (FA) and 1% ACN (LTQ Orbitrap XL, Thermo Fisher Scientific) or 1% ACN with 0.05% TFA (Orbitrap Fusion Lumos, Thermo Fisher Scientific) on a 75 µm × 2 cm Acclaim^®^ PepMap RSLC column (Thermo Fisher Scientific) at a flow rate of 4 µl/min for 10 min at 50 °C. Subsequently, peptides were gradually eluted from the separation column (Acclaim^®^ C18 PepMap RSLC column 50 µm × 25 cm, Thermo Fisher Scientific) with a linear gradient from 3 to 40% solvent B (0.15% FA with 80% ACN) at a flow rate of 175 nl/min (LTQ Orbitrap XL) or 300 nl/min (Orbitrap Fusion Lumos) over a course of 90 min. Between minutes 101 and 106, the column was washed with solvent B which mounted up to 95%.

Pre-fractionated peptides eluting from the separation column were introduced into an online-coupled tandem mass spectrometer equipped with a nanospray ion source and operated in data-dependent acquisition (DDA) mode.

Fragment spectra were searched against the Swiss-Prot release from September 27th 2013 (20,279 reviewed protein sequences) using the SEQUEST [10] search algorithm embedded in Proteome Discoverer 1.4.1.14 (Thermo Fisher Scientific) with methionine oxidation as the only dynamic modification. The false discovery rate estimated by the Percolator algorithm (2.04) was set to ≤ 5%. All peptide eluates were analyzed on an LTQ Orbitrap XL, and residual sample volume was measured on an Orbitrap Fusion Lumos. For those samples acquired on both devices, peptide and protein lists were merged manually with peptide-specific scores reported for every LC–MS/MS system. HLA class I datasets were deconvoluted using a stand-alone version of NetMHCpan-4.0 (percentile rank score ≤ 2%) [16, 19] and an enhanced version of SYFPEITHI (SYFPEITHI score ≥ 60%) [34].

### Peptide synthesis

Peptides were synthesized by INTAVIS Peptide services (Tübingen, Germany) using standard solid-phase Fmoc synthesis in order to be used in immunogenicity analyses. Quality control was performed using MALDI-MS and RP-HPLC.

### Gene ontology enrichment analysis

A list of manually curated genes (188 identifiers) was used to study enriched gene ontology (GO) terms with Metascape [54]. Metascape identifies all significantly enriched GO terms (GO/KEGG terms, canonical pathways, hallmark gene sets, etc.) through accumulative hypergeometric *p* values and enrichment factors used for filtering. The filtered terms were hierarchically clustered into a tree based on Kappa-statistical similarities among their gene membership. The threshold to cast the tree into term clusters was set as 0.3 kappa score.

### Unsupervised hierarchical clustering

Unsupervised hierarchical clustering was used to analyze the qualitative source protein representation of the HLA ligandomics datasets. The InstantClue v.0.11.0 software (www.instantclue.uni-koeln.de) was employed for this purpose and default settings were adopted. For the HLA class I source protein representation, only binders were considered. For both HLA classes, only peptides mapping into multiple proteins were excluded. Similarly, for HLA class II, only single mappers were selected for analysis. In addition, input datasets included patient metadata, such as patient sex, WHO grade, and tumor location, allowing the investigation of potential clustering of patient characteristics.

### T-cell mapping

Formalin-fixed, paraffin-embedded (FFPE) tissue microarrays (TMA) composed of meningioma tumor probes were cut in 2 µm sections and stained using the BOND Fully Automated IHC Staining System or Ventana Roche BenchMark ULTRA Fully Automated IHC Staining System. The sections were incubated with primary antibodies against CD4 (Ventana-Roche, Order No. 790-4423/05552737001, prediluted) and CD8 (DAKO A/S, Order No. M71030, dilution: 1:100). For the detection of T regulatory cells, antibodies were first applied against FOXP3 (Abcam Limited, Order No. ab2003, dilution 1:150) and then against CD3 (Thermo Scientific, Order no. MA1-90,582, dilution: 1:100). Visualization of the antibodies was performed with the VENTANA BenchMark ULTRA, the DF AP or DAB kit. All sections were counterstained with hematoxylin.

### T cell (CD8^+^), T helper cell (CD4^+^), and regulatory T cell (CD3^+^FoxP3^+^) quantification

Image processing of the scanned slides was performed using QuPath v0.4.3, an open-source quantitative pathology and bioimaging software [3]. Each slide was dearrayed and the grid manually adjusted for optimal analysis. Setup parameters for each stain, to determine positively stained cells, were adjusted to a high sensitivity while minimizing background. Single positive staining (CD4 and CD8) was defined by thresholding red staining, while double-positive (CD3 FoxP3) cells were manually included. For each core, the percent of positive cells was assessed to quantify protein expression. Percentages from each core were curated into a table and organized into their respective cohort (WHO Grade I, II, and III).

### CD8^+^ T-cell priming using aAPC

Priming of CD8^+^ T cells was performed using artificial antigen-presenting cells (aAPC) following a standard protocol [49] and using empty loadable MHC monomers (Tetramer Shop ApS, Denmark). In brief, human peripheral blood mononuclear cells (PBMCs) from fresh buffy coats of healthy donors (HLA-A*02 restricted) were isolated via density gradient centrifugation on day 1. Some peptides were tested using PBMC deriving from different healthy donors. At day 2, CD8^+^ T cells were MACS enriched by positive selection (Miltenyi Biotec, Bergisch Gladbach, Germany). In vitro stimulations were initiated on day 3 using 10^6^ CD8^+^ T cells with 8 × 10^5^ beads and 5 ng/ml IL-12 p70 (R&D Systems) in a 96-well plate in 200 µl IMDM containing 25 mM HEPES (Thermo Fisher Scientific) with 10% heat-inactivated human AB plasma (Sigma-Aldrich), 2 mM l-glutamine, 50 U/ml penicillin, and 50 ug/ml streptomycin. At least 20 wells, but most commonly 30 wells were seeded for each candidate peptide that was tested. After 3 days of co-incubation at 37 °C, fresh medium supplemented with 40 U/ml human IL-2 was added, and cells were incubated for 4 days. This stimulation cycle was repeated three times and tetramer staining was performed on day 28.

### Tetramer staining

The frequency of peptide-specific CD8^+^ T cells was assessed by staining with PerCP anti-human CD8a antibody (Biolegend, RPA-T8) and loading the empty loadable PE-conjugated tetramers (Tetramer Shop ApS, Denmark) with the candidate peptide [27]. Samples were analyzed on a BD FACSverse (BD Biosciences). Cultures were considered positive if > 1% of tetramer-positive cells among the CD8^+^ cells were detected and if the double-positive (PerCP-PE) population resulted in a threefold increase compared with the negative control which was a peptide that responder cells were not primed for before. Flow cytometric data analysis was performed using FlowJo (v.10.0.8) (Tree Star Inc., Ashland, OR, USA).

## Results

This study is based on a three-phase antigen discovery strategy including (1) HLA peptidome profiling, (2) candidate peptide selection, and (3) functional immunogenicity testing. Based on this approach, we generated a T-cell antigen atlas for meningioma including a set of meningioma-exclusive antigens naturally presented on HLA class I or II molecules and assessed their immunogenicity in vitro.

### HLA-IPs yield a broad set of high-purity HLA class I and II ligands

**Fig. 1:**
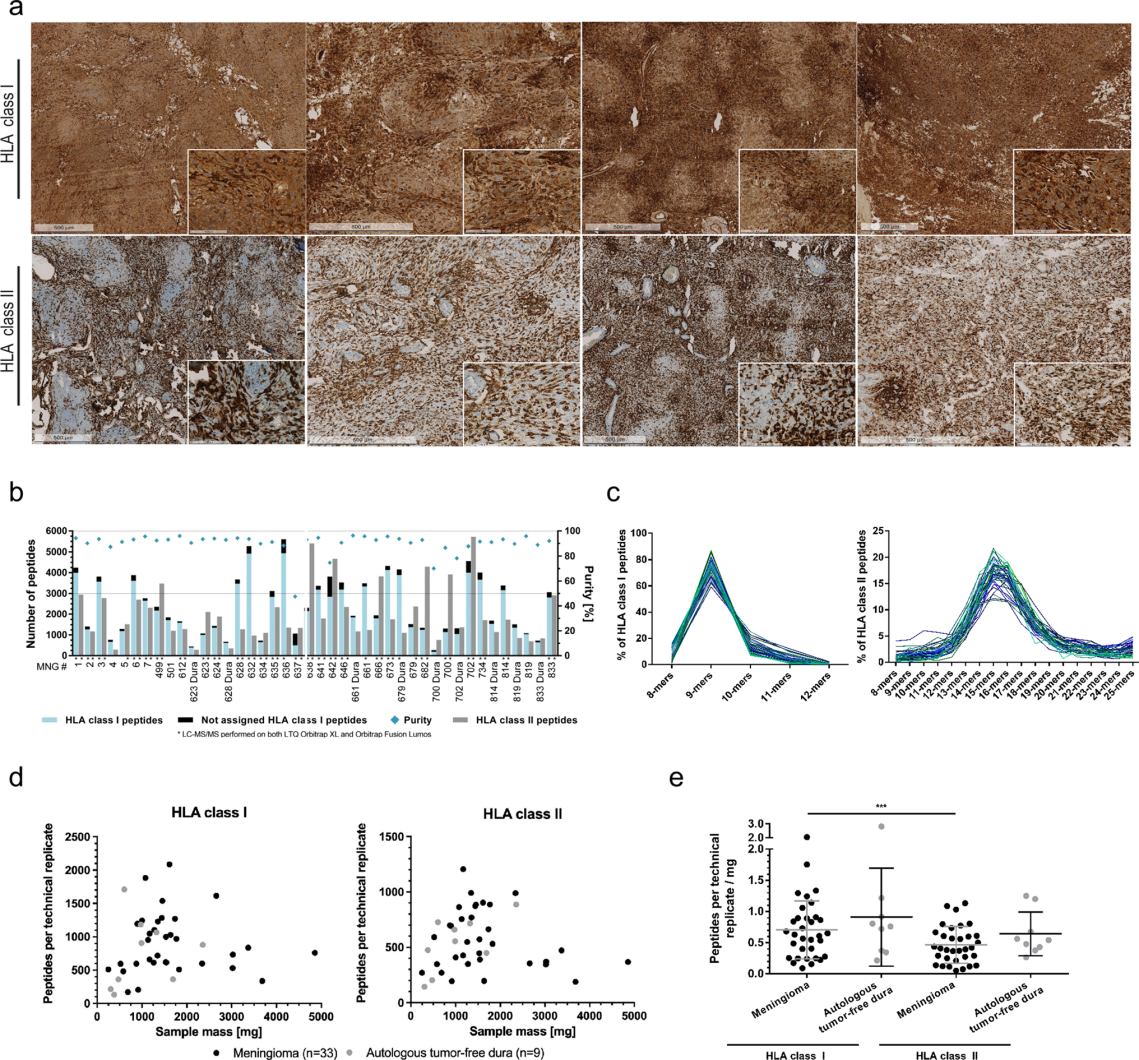
HLA ligandome profiling. **a** HLA class I (upper panel) and II (bottom panel) proteins were assessed on four patients by immunohistochemistry. **b** HLA class I and II peptide yield of meningioma and autologous tumor-free dura tissue. Calculated purities refer to the proportion of HLA class I peptides annotated to an HLA allotype of the respective patient. **c** Length distribution of HLA class I (on the left) and II (on the right) peptides. Each blue line represents data from one tumor sample, whereas the green line corresponds to tumor-free dura sample. **d** Correlation analysis between unique peptides per sample and technical replicate versus amount of tissue subjected to HLA-IP (two-tailed *p* values = 0.8875 (meningioma HLA class I)/0.6039 (dura HLA class I)/0.2862 (meningioma HLA class II)/0.0542 (dura HLA class II). **e** Peptide yields per technical replicate normalized to a tissue mass of 1 mg used as analysis input for both HLA class I and HLA class II peptides

The patient cohort comprised 33 individuals with a female-to-male ratio of 2:1 and a median age at diagnosis of 59 years (34–83) (Supplementary Table 1, Supplementary Table 2 online resource 1). HLA class I and II expressions were assessed via immunohistochemistry as a prerequisite for HLA peptidome profiling. A robust membrane staining was detected for both HLA class I and II molecules (Fig. 1a). A semiquantitative immunohistochemistry analysis of a meningioma tissue microarray, that also included the majority of tumor samples of this cohort, was performed to investigate the immune cell infiltration. Moderate infiltration of CD4^+^ and CD8^+^ T cells and almost absent regulatory T cells were observed (Supplementary Table 3. Online source 1). Except for a trend toward higher T helper cell (CD4^+^) infiltration in WHO grade 3 meningiomas, no correlation with WHO grading was detected.

HLA class I- and II-presented peptides were isolated from 33 primary tumor and nine autologous tumor-free dura specimens and analyzed by HLA immunopurification (HLA-IP) and LC–MS/MS. The HLA typing analysis revealed 58 distinct HLA class I allotypes covering 99.98% of the world population, whereby 94.6% of all individuals worldwide are expected to be positive for at least three allotypes (Supplementary Fig. 1, online resource 2). The most frequent allotype among meningioma patients was HLA-A*02:01 with 20% representation (Supplementary Table 4, online resource 1).

HLA class I peptide eluates had at least 70% purity (percentage of peptides that have a solid predicted binding motif matching the patient’s HLA typing, except MNG637) and none of the samples were excluded for low yield or low purity. A median of 2649 (505–4939) and 1038 (185–3891) HLA class I ligands were identified from meningioma and dura samples, respectively (Fig. 1b). For HLA class II peptides, a median of 1,882 (301–5,720) and 852 (284–1371) peptides were isolated from meningioma or dura tissue, respectively. The length distribution of eluted peptides was in accordance with typical HLA binders, with HLA class I ligands revealing a clear peak at nine amino acids (AA), while HLA class II ligands were found to be mostly 13- to 18-mers (Fig. 1c).

No correlation was found between tissue mass subjected to HLA-IP and the number of HLA class I or II peptide identifications per technical replicate (Fig. 1d). However, when the number of identified unique peptides per technical replicate and mg of tumor tissue input was considered, an increased yield of HLA class I as compared with HLA class II peptides was observed. No significant difference was observed when the yields of HLA class I and HLA class II peptides of autologous dura were compared (Fig. 1e).

### Tumor association is defined based on the immunological pivotal level of the natural HLA peptidome

Although the abundance and turnover of source proteins have been shown to correlate partially with HLA class I-restricted peptide presentation [5], there is still a high degree of variability in sampling for HLA-restricted presentation for individual proteins. Importantly, the overall correlation of precursor abundance (RNA and protein) with HLA ligand abundance for individual genes/proteins is poor [11, 38, 51]. This limits the value of in silico predictions based on RNA sequencing data and warrants direct analysis of HLA peptidomes for antigen discovery. Therefore, we strictly relied on HLA peptidome analyses to identify naturally presented targets by comparative HLA peptidome profiling of patient samples concerning a comprehensive database of various normal human tissues, including the brain. To define meningioma-associated antigens and peptides, an in-house benign database was constructed using 30 distinct primary human organs (*n* = 418 HLA class I and *n* = 364 HLA class II peptidome datasets) supplemented with nine autologous tumor-free dura samples. The rationale for including tumor-free dura was to exclude dura-associated antigens and to reduce the likelihood of T-cell-mediated meningitis during immunotherapy.

### Mapping the antigenic landscape of naturally presented HLA class I ligands reveals meningioma-exclusive antigens

**Fig. 2:**
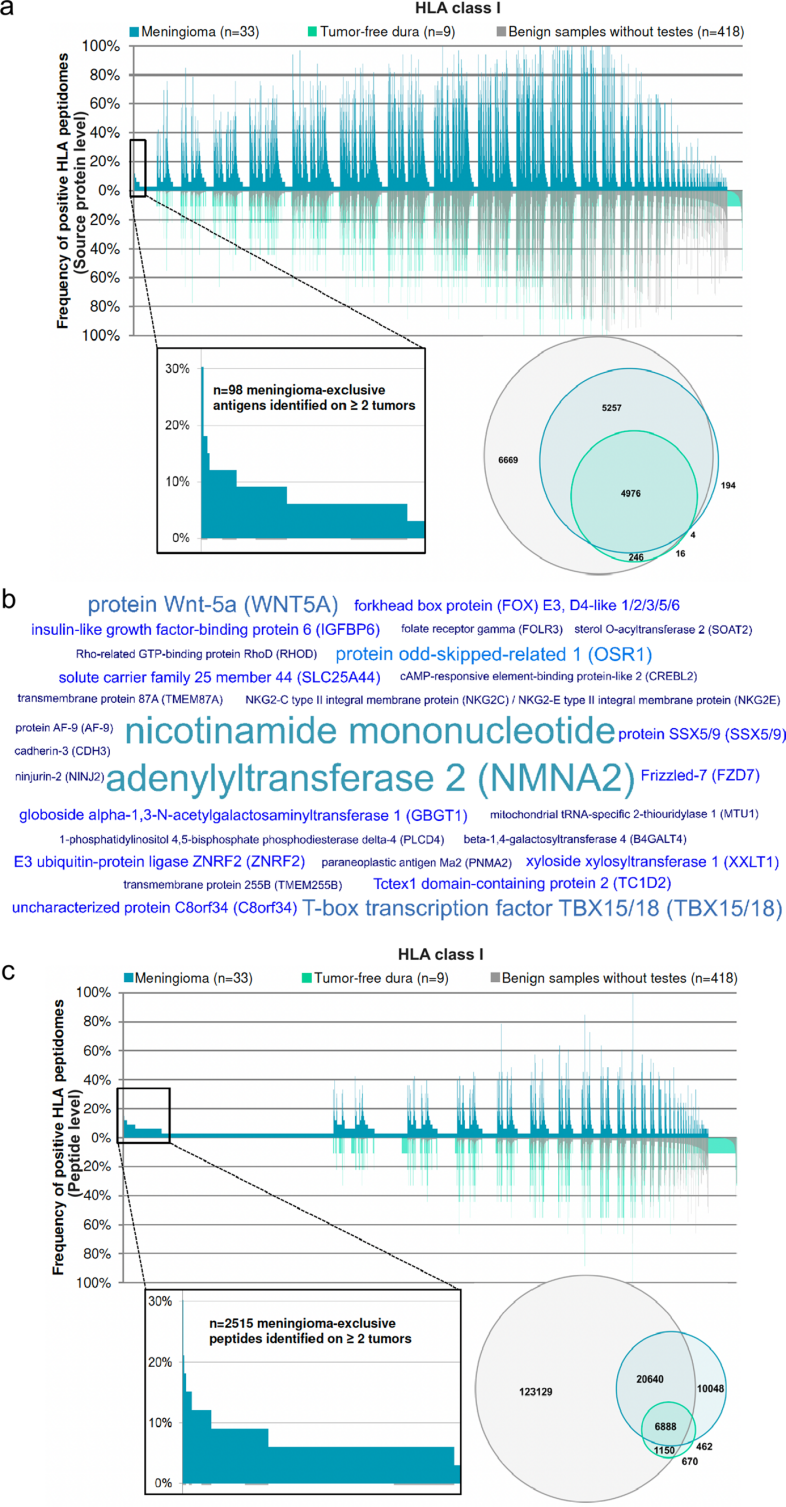
Definition of meningioma-associated antigens and peptides based on class I immunopeptidome analyses. **a** Comparative profiling of the HLA class I antigens of meningioma versus the in-house benign database complemented by tumor-free dura. Each bar in the waterfall plot (x-axis) represents a single source protein. In contrast, the y-axis depicts the frequency of positive HLA peptidomes, separately for meningioma (*n* = 33), tumor-free dura (*n* = 9), and benign samples without testes (*n* = 418 covering 29 different human tissues). Source proteins detected on a maximum of one non-CNS-related tissue were considered as meningioma-exclusive, whereby *n* = 98 was identified on at least two meningeal tumors (enlarged view on the left). The Venn diagram on the right shows the number of all the source proteins detected. **b** The word cloud shows a set of 28 meningioma-associated antigens naturally presented on 9–30% of meningeal tumors based on comparative profiling and subsequent quality control of underlying peptides. The font size in the word cloud is proportional to the frequency of positive meningiomas. **c** Comparative analysis of the HLA class I peptides presented on meningioma versus the in-house benign database supplemented with tumor-free dura. Peptides were designated as meningioma-exclusive when detected on a maximum of one non-CNS-related tissue, whereby *n* = 2515 were identified on at least two meningeal tumors (enlarged view on the left). The number of distinct HLA class I ligands per group is illustrated by the Venn diagram on the right

HLA class I peptidome profiling of meningioma (*n* = 33) and autologous tumor-free dura (*n* = 9) identified 10,431 and 5242 distinct source proteins on neoplastic and tumor-free meningeal tissue, respectively (Fig. 2a). These yields represent 82% (dura) and 93% (tumor) of the estimated maximum attainable source protein coverage (Supplementary Fig. 2, online resource 2).

Subtraction of the in-house benign database supplemented by tumor-free dura samples revealed 98 antigens (source proteins) exclusively presented by at least two meningeal tumors (Fig. 2a). Comparative profiling and subsequent quality control of the underlying peptides for HLA motifs as well as eliminating peptides that multi-mapped to several source proteins yielded a set of 28 meningioma-associated source proteins and corresponding peptides naturally presented on 9–30% of tumors. This implies that all the peptides deriving from these 28 meningioma-associated antigens are meningioma-associated. Among these, NMNA2, WNT5A, TBX15/18, and OSR1 were the most frequent (Fig. 2b). Interestingly, seven of the meningioma-associated antigens were shared between all WHO grades (NMA2, TBX15/18, XXLT1, SLC25A44, RHOD, CREBL2, and PLCD4). WHO grade I and II meningiomas had 16 antigens in common with three antigens exclusively presented by WHO grade I meningiomas (TC1D2, B4GALT4, and MTU1) (Supplementary Table 5, online resource 1)**.**

The investigation of meningioma-associated HLA class I-presented peptides resulted in 38,038 and 9170 distinct HLA class I ligands derived from meningeal neoplasms and tumor-free dura, respectively. The number of ligands eluted from meningioma samples and dura corresponded to 77% and 38% of the predicted maximum attainable coverage (Supplementary Fig. 3, online resource 2). Despite the significant overlap of the meningioma HLA class I peptidome and the in-house benign database, 2515 meningioma-exclusive peptides were identified in at least 2 tumors (Fig. 2c). Focusing on the peptides found in at least 5 patients, a panel of 74 peptides derived from 68 antigens was identified in 15–30% of tumors (Supplementary Table 6, online source 1). While these peptides are meningioma-associated, the antigens from which they derive may be presented on both healthy and tumor tissues. Among these, HLA ligands derived from OGN, FOXC2, and IFI44L were the most abundant. Overall, 31 peptides were commonly presented across all WHO grades, 36 peptides were shared by WHO grade I and II meningiomas, and two peptides were identified in both WHO grade I and III meningiomas. One peptide was exclusively presented in WHO grade III meningiomas, whereas four were specific for WHO grade I meningiomas.

Our meningioma-associated HLA class I peptides cover 99.57% of the world population regarding HLA allotype distribution (Supplementary Fig. 4, online resource 2), with an average of 21 peptides expected to match per patient. The population coverage on a per-country basis is shown in Supplementary Fig. 5, online resource 2.

### The HLA class II peptidome includes meningioma-associated antigens that can be used to elicit a synergistic CD4^+^ T-cell response

**Fig. 3:**
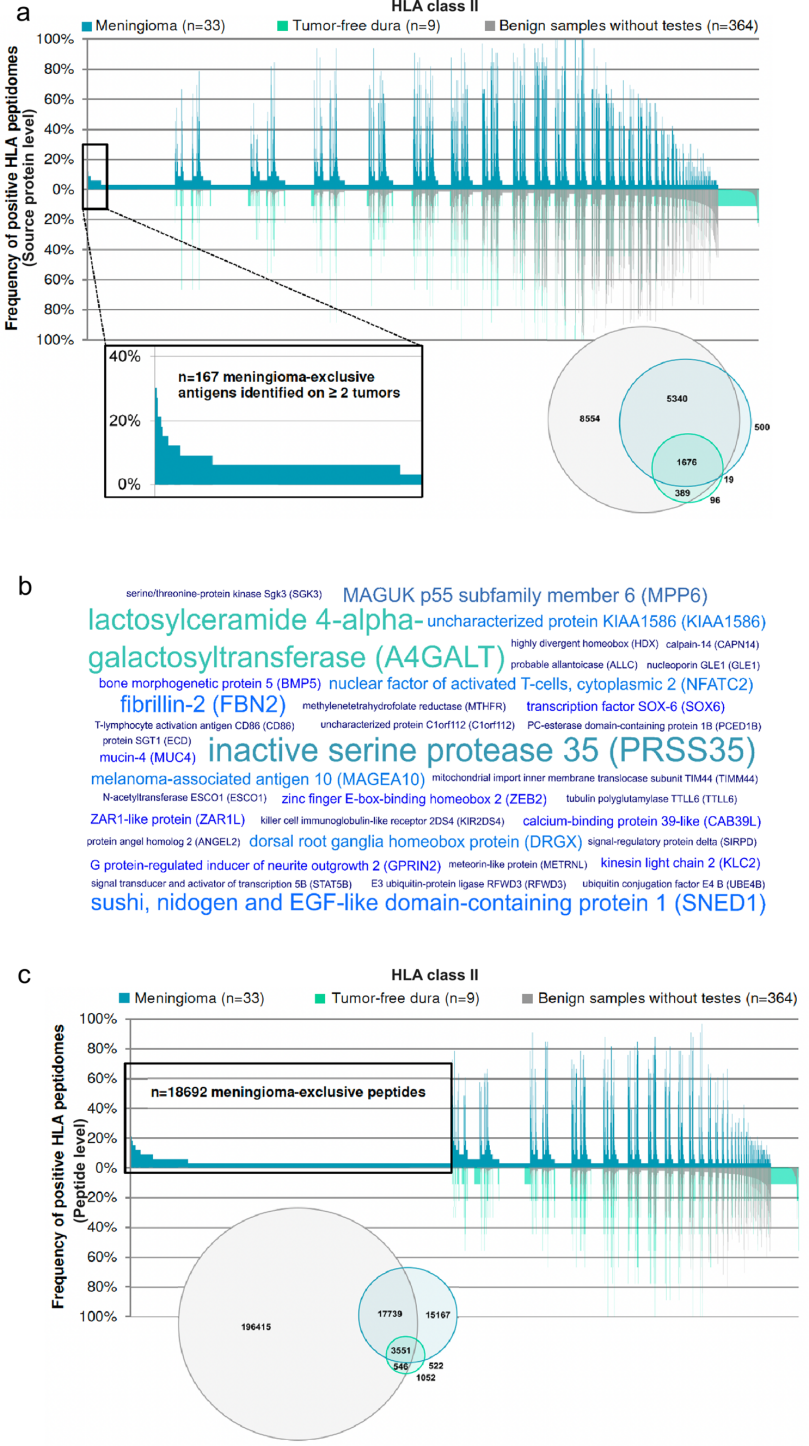
Definition of meningioma-associated antigens and peptides based on class II immunopeptidome analyses. **a** The comparative profiling of the HLA class II antigens of meningioma versus the in-house benign database supplemented with tumor-free dura revealed meningioma-exclusive source proteins (*n* = 167) identified on at least two meningeal tumors and a maximum of one non-CNS-related tissue (enlarged view on the left). Each bar in the waterfall plot (x-axis) represents a single source protein, whereas the y-axis depicts the frequency of positive HLA peptidomes, separately for meningioma (*n* = 33), tumor-free dura (*n* = 9), and benign samples without testes (*n* = 364 covering 30 different human tissues). The Venn diagram on the right depicts the number of distinct HLA class II-presented antigens per group. **b** The world cloud shows a set of 37 meningioma-associated antigens naturally presented on 9–30% of meningeal tumors based on comparative profiling and subsequent quality control of underlying peptides. The font size in the word cloud is proportional to the frequency of positive meningiomas. **c** The comparative analysis of HLA class II peptides presented on meningioma versus the in-house benign database supplemented with tumor-free dura identified *n* = 18,692 meningioma-exclusive peptides detected on a maximum of one non-CNS-related tissue. Corresponding source proteins were subjected to hotspot analysis. The total number of HLA class II-restricted peptides per group is illustrated by the Venn diagram on the left

HLA class II peptidome analysis identified 7535 and 2180 distinct source proteins for meningiomas and autologous tumor-free dura samples, respectively. These represent 72% (meningiomas) and 70% (tumor-free dura) of the estimated maximum attainable source protein coverage (Supplementary Fig. 6, online resource 2). An extensive comparison revealed 167 antigens exclusively presented by at least two meningeal tumors (Fig. 3a). Upon manual curation of the underlying peptides according to peptide length and the presence of length variants as well as multi-mapping to several source proteins, 37 meningioma-associated antigens and corresponding peptides naturally were identified on 9–30% of tumors (Supplementary Table 5, online source 1).

Among these, PRSS35, A4GALT, FBN2, and SNED1 were most frequent (Fig. 3b). Investigation of antigen distribution across all WHO grades yielded six common meningioma-associated antigens (A4GALT, FBN2, SNED1, MPP6, PCED1B, and ANGEL2). When only WHO grade I and II tumors were considered, 24 shared antigens were detected. Six antigens were exclusively found in the immunopeptidome of WHO grade I meningiomas (CAPN14, KIR2DS4, TMM44, ECD, CD86, and RFWD3), whereas WHO grade I and III meningiomas showed only one antigen in common (KLC2).

On the peptide level, 36,979 and 9179 distinct HLA class II-presented peptides were eluted from meningiomas and tumor-free dura, accounting for 54% and 50% of the estimated maximum attainable coverage (Supplementary Fig. 7, online resource 2). 18,692 peptides detected on a maximum of one non-CNS-related entity were designated as meningioma-exclusive (Fig. 3c). Comparative profiling is not capable of reflecting length variants and common core sequences which is why we aimed at grouping peptides sharing a core sequence. Meningioma-exclusive HLA class II-presented peptides deriving from comparative profiling were subjected to a ‘hotspot analysis’ according to which peptides were grouped by a common core sequence. Tumor-associated HLA-presentation hotspots were defined as having a minimum length of eight AA and being covered by peptides identified in at least five patients while not having matching sequences in benign samples. This generated a set of 44 antigens harboring regions exclusively presented on tumor tissue with peptide-specific frequencies reaching up to 45% of positive HLA class II peptidomes. Tumors of all WHO grades shared 15 proteins, whereas presentation hotspots within 26 proteins were found in WHO grade I and II tumors. One meningioma-associated HLA class II presentation hotspot was exclusively detected in WHO grade I meningiomas, whereas WHO grade I and III tumors had two hotspot targets in common (Supplementary Table 7, online resource 1).

A comparative analysis between meningioma-associated HLA class I- and II-presented antigens as well as meningioma-associated HLA class I- and II-restricted peptides revealed a unique antigenic repertoire of the two HLA classes. STAB1 was the only shared antigen yielding both meningioma-associated HLA class I and II ligands. This suggests that mapping both HLA classes should be considered complementary, not only on the functional level of distinct sets of T-cell specificities (CD8^+^ vs. CD4^+^) but also on the level of the individual antigens.

### Meningioma-associated antigens show a high identification rate of established CTA and TAA but overall low presentation frequencies

**Fig. 4:**
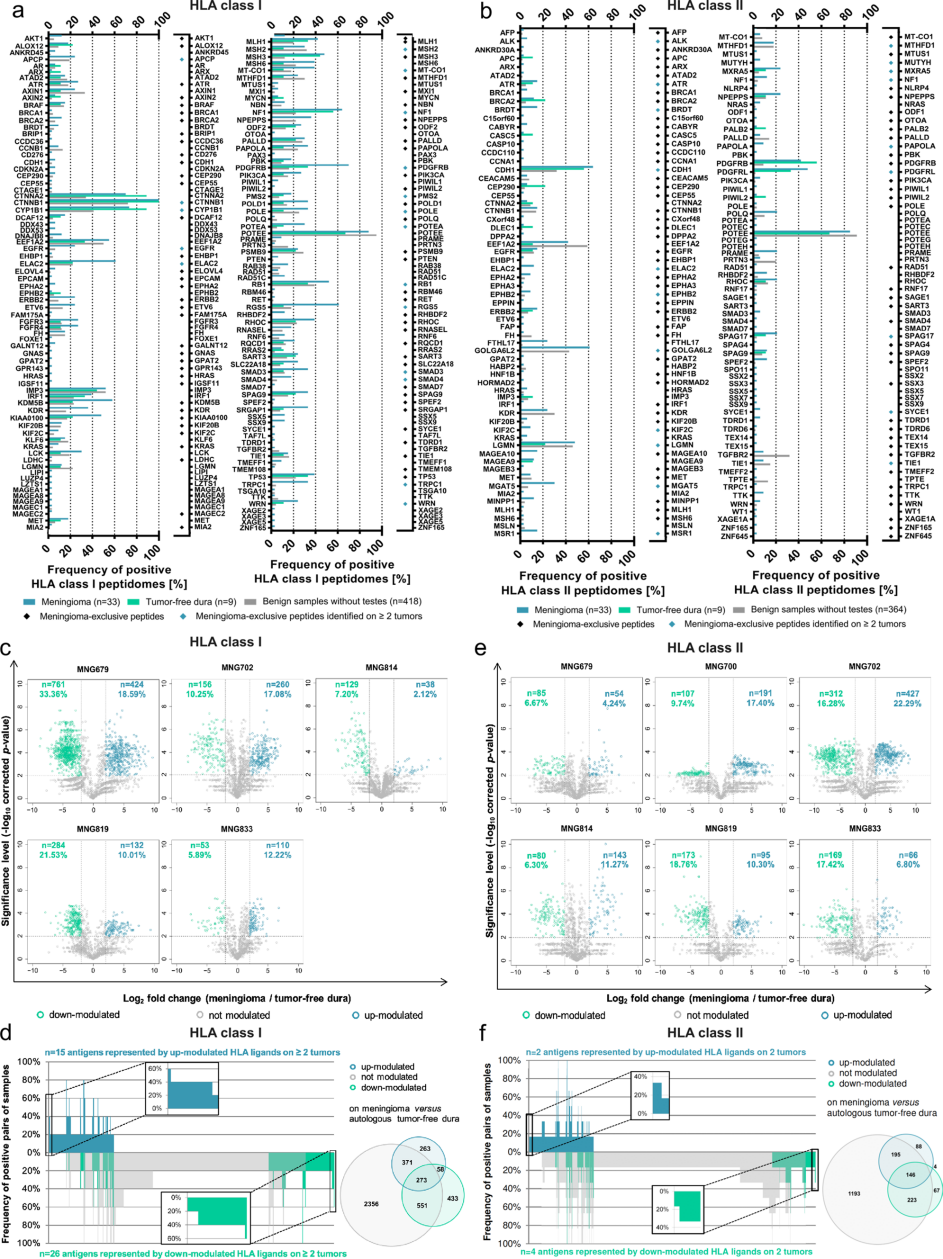
Identification of established TAA, CTA, and meningioma-associated antigens across the present HLA peptidome dataset. While peptides mapping to multiple source proteins were considered to calculate the frequency of positive HLA peptidomes, these were excluded from reporting the representation by meningioma-exclusive peptides. CTA and TAA exclusively identified on benign samples were not listed. **a** The present immunopeptidomic dataset includes HLA class I ligands derived from 145 TAA and CTA, of which 18 are represented by meningioma-exclusive peptides on at least two tumors. The frequency of positive HLA peptidomes was assessed based on HLA class I ligands for meningioma and dura samples, whereby benign hits were reported independent of HLA binding probabilities of the underlying peptide identifications. **b** TAA and CTA are represented in the HLA class II peptidomes of human meningioma and/or tumor-free dura tissue. Among 126 naturally presented CTA and TAA, 20 were represented by meningioma-exclusive peptides on at least two tumors. Patterns of modulated HLA class I (**c**) and class II (**e**) presentation in meningioma across five and six patients, respectively. The volcano plots represent the relative HLA ligand abundances on meningioma versus autologous tumor-free dura. By LFQ-MS, relative abundances of HLA class I and II ligands, each represented by a dot, were compared. The x-axis indicates changes of abundance as log_2_ fold change and corresponding significance levels (after BH correction for multiple testing) are associated with the y-axis. Significant modulation was defined by a corrected *p* value ≤ 0.01 and a fold change of mean AUC in (meningioma/dura) ≥ 4 or ≤ 0.25 regarding up- (highlighted in blue) or down-modulated (highlighted in green) peptides, respectively. The total number of up- and down-modulated peptides as well as their proportion in the patient’s HLA class I and II peptidome are indicated in quadrants of each Volcano plot. Comparative profiling of HLA class I (**d**) and II (**f**) antigens corresponding to peptides displayed in volcano plots. Each bar in the waterfall plot represents a single protein, whereas the frequency of positive pairs of samples is shown on the y-axis. Comparing the source proteins of peptides underlying significant up- or down-modulation and of those being modulated allowed the identification of exclusively and recurrently over- or under-represented antigens. The comparative profiling of HLA class I revealed *n* = 15 up-modulated proteins and *n* = 26 down-modulated antigens across five LFQ datasets acquired from meningioma and autologous tumor-free dura. When HLA class II was considered instead, *n* = 2 up- and *n* = 4 down-modulated antigens were identified across six patients

The present HLA peptidome dataset was screened for previously established tumor antigens. Considering a total number of 366 published cancer-testis antigens (CTA) and tumor-associated antigens (TAA) as well as 15 antigens reported to be associated with meningeal neoplasias [1, 8, 14, 48], 145 were represented by HLA class I ligands and 126 by HLA class II-presented peptides in our dataset. Despite these high identification rates, presentation frequencies of CTA and TAA were generally low, especially of those exclusively identified on meningeal tumors. Among 23 HLA class I-presented TAA and CTA, SSX5, SSX9, DDX43, and DDX53 were the most abundant (6–12% positive tumors). In addition, 18 TAA and CTA were represented by meningioma-exclusive HLA class I ligands on two to four meningiomas (Fig. 4a, Supplementary Table 8, online resource 1). Regarding HLA class II, 26 antigens were exclusively identified in the peptidome of meningiomas, with MAGEA10, MUTYH, and CABYR being the most frequent (6–15% positive tumors). Furthermore, 20 CTA and TAA were represented by meningioma-exclusive HLA class II ligands on two to five tumors (Fig. 4b, Supplementary Table 8, online resource 1).

### Label-free quantification in meningioma and autologous tumor-free dura peptidomes reveals significantly up- and down-modulated ligands

To obtain a deeper insight into general patterns of modulated HLA presentation on meningioma versus non-neoplastic dura, significantly modulated peptides were assigned to the source proteins. These protein lists were combined for all patients and subjected to comparative profiling unveiling HLA class I and II antigens recurrently represented by up- or down-modulated peptides.

Significant modulation was defined by a corrected *p* value ≤ 0.01 and a fold change of mean AUC in (meningioma/tumor-free dura) ≥ 4 or ≤ 0.25 regarding up- or down-modulated peptides, respectively. The analysis was performed on five patients for which both tumor tissue and tumor-free dura were available. On average, 12.00 ± 5.85% and 15.65 ± 10.43% of the patients HLA class I peptidomes (1563 ± 462 HLA class I ligands evaluated) showed significant up- or down-modulation, respectively (Fig. 4c). A maximum of two HLA-A, -B, or -C allotypes were shared between these five patients. Therefore, comparative profiling was performed on the source protein level. In total, 263 and 433 antigens were up- or down-modulated (Fig. 4d).

Upon manual curation of the underlying peptides for HLA motifs as well as multi-mapping to several source proteins, 40–60% of patients presented a set of 13 antigens that were up-modulated in meningioma versus autologous tumor-free dura. Among these, RLA2 was the most frequent one. Conversely, 18 proteins were under-represented when comparing meningioma and tumor-free dura HLA ligandome profiles. PRC2C was represented by down-modulated HLA-A*23:01 and -A*24:02 ligands on three out of five meningiomas (Supplementary Table 9, online resource 1).

For HLA class II ligands, modulation analysis was performed on six patients for which both meningioma and tumor-free dura were available. Of 1,242 ± 330 HLA class II-presented peptides evaluated, 12.05 ± 6.13% and 12.53 ± 5.13% of the patients’ HLA class II peptidomes were subject to significant up- and down-modulation (Fig. 4e). Overall, 88 and 67 antigens were exclusively represented by up- and down-modulated peptides (Fig. 4f).

No common signature of modulated HLA class II presentation was identified across the tumors from six patients, with only two (VQA1 and QCR8) and four (CATF, ACH10, BROMI, and NSF) antigens being represented by up- or down-modulated peptides in two cases (Supplementary Table 9, online resource 1).

### Functional annotation clustering of up- and down-modulated proteins and GO term enrichment analysis revealed significant correlations with cellular and molecular programs

**Fig. 5:**
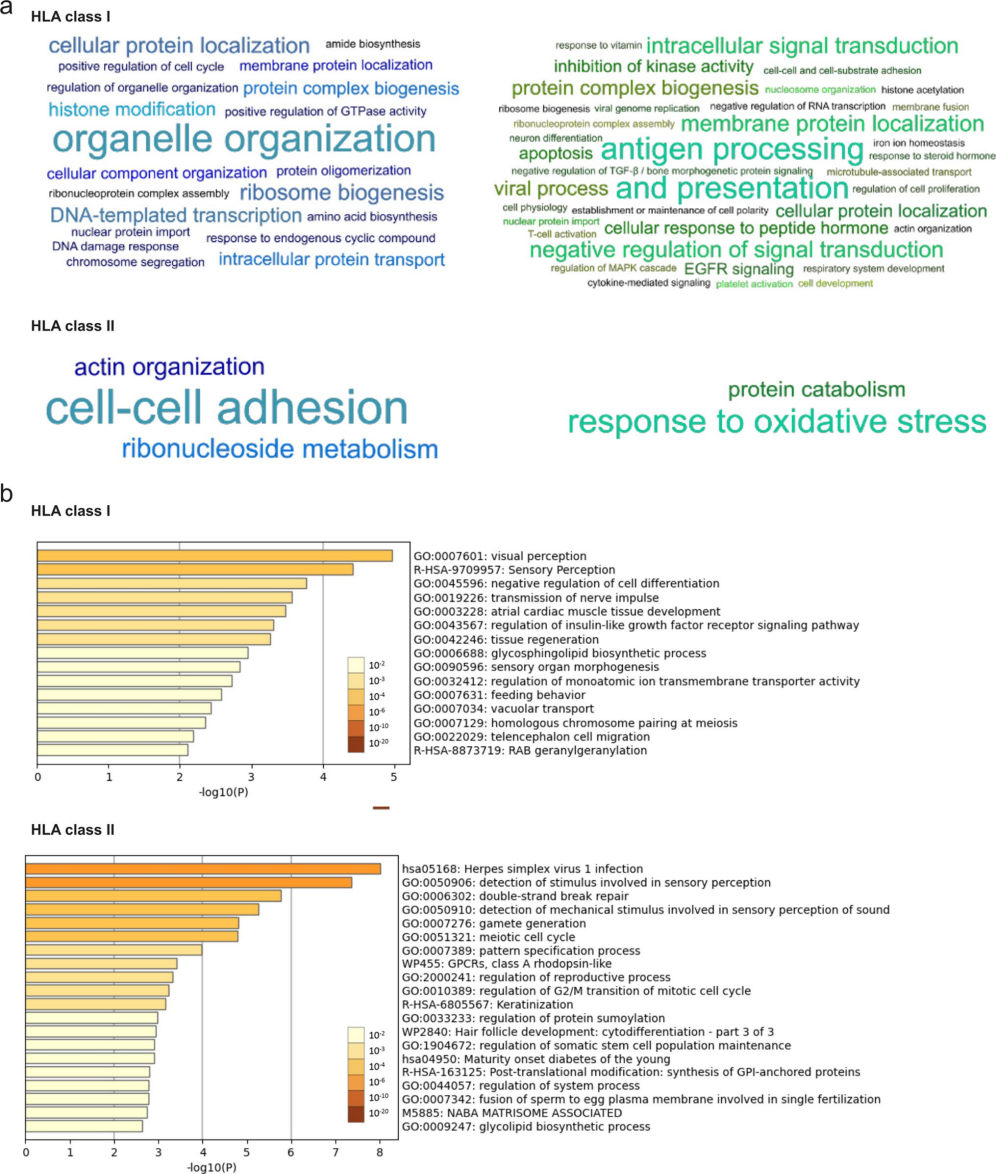
Biological annotation clustering and GO terms enrichment analysis. **a** HLA class I functional annotation of antigens exclusively represented by up- or down-modulated peptides (upper panel). Modulation-exclusive source proteins (*n* = 263 over-represented antigens shown in the left and *n* = 433 under-represented antigens shown in the right panel) were clustered for functional annotation with enrichment scores proportional to the font size in the word clouds. Also, for HLA class II, modulation-exclusive source proteins (*n* = 88 over-represented antigens shown in the left and *n* = 67 under-represented antigens shown in the right panel) were clustered for functional annotation in the word clouds (bottom panel). **b** Metascape bar graphs depicting and listing the top significant non-redundant enrichment clusters for HLA class I (upper panel) and HLA class II (bottom panel). The discrete color scale represents the degree of statistical significance

HLA class I protein functional annotation clustering showed that up-modulated proteins were mainly associated with organelle organization, cellular protein localization, ribosome biogenesis, DNA templated transcription, and histone modification. For down-modulated antigens, a correlation was found with antigen processing and presentation, intracellular signal transduction, negative regulation of signal transduction, and membrane protein localization (Fig. 5a). Investigation of biological annotation clustering for HLA class II proteins revealed only three up-regulated clusters (cell–cell adhesion, ribonucleoside metabolism, and actin organization) and two down-modulated clusters (response oxidative stress, protein catabolism) (Fig. 5a). To account for the high peptide-level heterogeneity, unsupervised hierarchical clustering based on HLA class I and II source proteins was also performed. No significant clustering was observed when considering tumor locations, WHO grades, and sex (Supplementary Fig. 8, online source 2).

GO ontology terms analysis unveiled significant top-enriched non-redundant clusters for both HLA class I and II gene sets (Fig. 5b). The three top-enriched GO terms for HLA class I were visual perception (GO: 0007601, *p* = 1.06*10^–5^), sensory perception (R-HAS-9709957, *p* = 3.82*10^–5^), and negative regulation of cell differentiation (GO: 0045596, *p* = 1.71*10^–4^). The three HLA class II top-enriched GO terms were herpes simplex virus 1 infection (hsa05168, *p* = 9.68*10^–9^), detection of stimulus involved in sensory perception (GO: 0050906, *p* = 4.30*10^–9^), and double-strand break repair (GO:0006302, *p* = 1.72*10^–6^) (Fig. 5b).

### Functional immunogenicity assays of meningioma-exclusive candidate peptides display antigen-specific immune recognition by CD8^+^ T cells

**Fig. 6:**
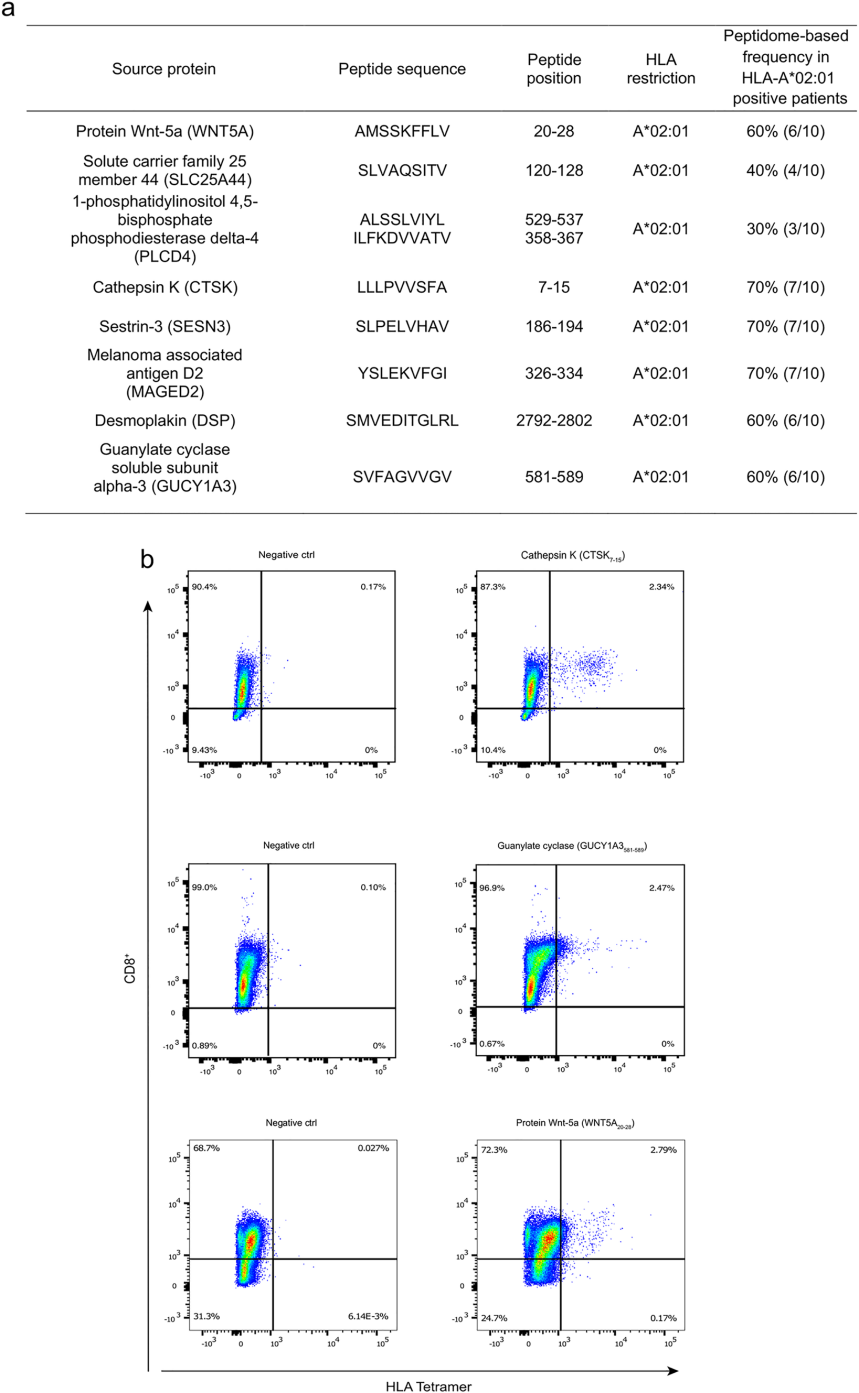
T-cell priming of top-ranking HLA-A*02:01-restricted candidates. Immunogenicity was assessed by priming CD8^+^ T cells from healthy donors with aAPC. These aAPCs consisting of polystyrene beads were coated with peptide-loaded HLA monomer and anti-CD28 antibodies. T-cell responses were defined via tetramer staining and were considered positive when a threefold increase compared to the negative control and > 1% of tetramer-positive cells among CD8^+^ T cells were observed. **a** List of the nine top-ranking HLA-A*02:01-restricted peptides showing protein source, peptide sequence, peptide position, HLA allotype, and peptide frequency in meningiomas. **b** Tetramer staining of CTSK_7-15_, GUCY1A3_581-589_, and WNT5A_20-28_ compared to a negative control (a peptide from the top-ranking pool for which CD8^+^ T cells were not primed prior functional testing)

Nine frequently detected HLA-A*02:01-restricted meningioma-associated peptides were synthesized and analyzed for immunogenicity. These peptides were presented on 30–70% of the analyzed HLA-A*02:01-restricted tumors (Fig. 6a). Peptide-specific immunogenicity was assessed via priming of naïve CD8^+^ T cells from healthy donors followed by HLA multimer staining (Fig. 6b). Priming with CTSK_7-15_, GUCY1A3_581-589_, and WNT5A_20-28_ showed a significant antigen-specific response (Fig. 6b), while the remaining 6 peptides did not display significant antigen-specific T-cell recognition (Supplementary Table 10, online resource 1). A negative T-cell priming result does not debunk a candidate, since a positive priming result depends on the presence of an antigen-specific naïve T-cell clone in the PBMC fraction that is sampled from healthy donors. However, the positive T-cell priming responses of the top-ranking candidates mentioned above functionally validate these antigens as prime targets for further translational investigations.

## Discussion

We mapped the T-cell antigen landscape of meningioma and defined meningioma-associated antigens and peptides based on an in-depth HLA peptidome analysis of 33 primary tumors. Comparative profiling between an in-house benign HLA peptidome database [26, 28] complemented with nine autologous tumor-free dura samples, and 33 meningioma specimens, identified new targets found exclusively in this tumor entity. Functional testing of a subset of these novel candidate antigens demonstrated the induction of antigen-specific T-cell responses pointing out their potential for T-cell-based immunotherapy (Fig. 6b).

Meningioma proved to be suitable for HLA peptidomics analyses, yielding considerable numbers of HLA class I- and II-presented peptides. 28 HLA class I and 37 HLA class II frequently presented meningioma-associated antigens were identified. Among these, NMNA2 (30%), PRSS35 (30%), A4GALT (27%), FBN2 (21%), SNED1 (21%) WNT5A (18%), TBX15/18 (18%), and OSR1 (15%) were most frequent (Fig. 2b, Fig. 3b, Supplementary Table 5, online source 1). Most newly discovered candidate antigens do not have an established biological role in meningioma or other tumor entities. A few exceptions are NMA2 or WNT5A. NMNA2 is known to promote cancer cell survival, whereas WNT5A is a member of the oncogenic WNT protein family and is expressed in meningioma and many other tumors [6, 7, 17, 18, 33, 42]. WNT5A has been found to induce tumor suppression and function as an oncogene depending on the specific cancer type [17].

The main level of comparison for defining tumor association is the in-house HLA ligandome database. HLA ligands from meningioma were also compared to tumor-free dura in this project. It is debatable whether arachnoid would be a good additional tissue to have as a control. However, it is surgically and ethically not feasible to harvest additional arachnoid tissue of patients that would be of sufficient quantity for HLA ligandome profiling (usually tissue amounts in the range of several hundred milligrams are necessary).

In addition, using dura as comparator to meningioma (most likely also based on the reasons mentioned above) is frequently used in the literature. For example, a recent single-cell RNA sequencing manuscript also compared meningioma to dura (in some cases also paired samples from the same patient) [50].

Besides meningioma-associated antigens, meningioma-associated peptides potentially arising from differential antigen processing in tumor cells were defined. The applied ranking criterion of “frequency” (number of positive samples for a given antigen in our cohort) for reported meningioma-exclusive HLA ligands is biased toward the most frequent HLA class I allotypes within the cohort (e.g., HLA-A*02). However, this increases the confidence of meningioma exclusivity by ensuring sufficient coverage by HLA peptidomes in the benign dataset. In addition, meningioma-exclusive targets from frequent allotypes have the advantage of being relevant to a large percentage of the patient population.

Searching acquired HLA class I and II peptidome data for meningioma-associated peptides unveiled a set of 74 HLA class I ligands derived from 68 antigens presented on 15–30% of tumors as well as 44 antigens harboring meningioma-associated HLA class II presentation hotspots yielding naturally presented peptides in at least five patients (Supplementary Tables 6, 7, online source 1). Remarkably, a comparison of meningioma-associated HLA class I- and II-presented antigens and meningioma-associated HLA class I- and II-restricted peptides revealed a unique antigenic repertoire inherent to HLA class I and II peptidomes. Thus, it is inevitable to consider both HLA classes on both antigen and peptide levels for comprehensive target discovery approaches. Notably, the potential of targeting HLA class II-restricted antigens was recently underscored by clinical data on the crucial role of HLA class II and CD4 + T cells in immune-mediated tumor rejection [22, 37, 43]. Furthermore, detailed knowledge of natural HLA class II antigen presentation patterns will allow for tailoring multi-epitope peptide vaccines containing both HLA class I- and HLA class II-restricted targets, which may induce and boost synergistic CD8^+^ and CD4^+^ anti-tumor responses.

Cancer testis antigens are frequently reactive genes in tumors whose expression is typically restricted to germ cells located in immunoprivileged sites. CTA are considered promising candidates for cancer immunotherapy, and the identification of novel CTA and TAA is a prerequisite for developing cancer vaccines [53]. Established CTA, TAA, and meningioma-associated antigens showed a high identification rate but overall low presentation frequencies, especially those exclusively identified on meningeal tumors. The HLA class II dataset contained three meningioma-associated CTA (C1orf112, SIRPD, and TTLL6) which have so far not been listed in the CTDatabase (www.cta.lncc.br). In contrast, none of the HLA class I-presented meningioma-associated antigens exhibited a CTA-like RNA expression profile. Remaining meningioma-exclusive antigens and peptides derived from established TAA and CTA were characterized by infrequent HLA presentation refuting these as prime targets for cancer immunotherapies. This again emphasizes that the immunopeptidome represents an autonomous layer strongly influenced by antigen processing and cannot be expected to mirror the transcriptome or even the proteome [5, 12, 40, 51]. Thus, the target definition for immunotherapeutic approaches is not recommended to be solely based on immunohistochemistry and protein or RNA expression data. These methods are, however, the basis for the definition of CTA and TAA. Our data illustrate the need for direct mapping of the antigenic repertoire naturally presented on HLA molecules.

It should be taken into consideration that our approach is not designed to detect mutation-derived neoepitopes. Neoepitopes are, in theory, tumor-specific and immunogenic, and the recent studies have shown promising clinical results in malignancies other than meningioma [35, 43, 46]. Our approach is neither designed nor able to detect neoepitopes, because the peptide data were processed against a reference proteome from normal tissues. However, this approach enables us to sensitively detect non-mutated antigens derived from “self”, which are well established in tumor immunology [45, 46] and have been shown to be targets of anti-cancer immune responses in leukemia patients [21]. Even in tumors with a high mutational burden, peptides derived from mutated sequences are exceedingly rare. In general, the number of mutated tumor antigens that elicit frequent and effective anti-tumor immune responses appears to be relatively small even in the setting of immune checkpoint inhibition [23]. A reason for the low prevalence of neoepitopes even in tumors with high mutational burden might be a clonal selection of neoantigen loss variants by immunoediting [47].

### Translational aspects

Systemic therapy for meningioma as an additional option to the established modalities of surgery and radiotherapy remains experimental to date with only very limited benefit. Promising results with partial tumor response have been obtained in patients undergoing therapy with bevacizumab or multikinase inhibitors targeting vascular endothelial growth factor receptor. However, convincing data are still missing [13, 20, 32, 52].

This study is not only the first description of T-cell antigens in meningioma, but it also provides actionable targets that can be further used for translation into the clinical setting. These results have the potential to contribute to the development of new therapeutic approaches in patients with inoperable or recurrent meningiomas (Fig. 6a) [2, 44].

By elucidating the natural antigenic landscape of meningioma, we have unveiled tumor-associated antigens and peptides that could be used to boost or guide the specific anti-tumoral T-cell response, e.g*.*, by combining immune checkpoint blockade and peptide vaccination or adoptive cellular approaches. A combined therapeutic approach is also conceivable with ongoing clinical trials investigating different immune checkpoint inhibitors, such as nivolumab, avelumab, or pembrolizumab (NCT03016091, NCT03267836, NCT03279692, and NCT03173950), or the combination of nivolumab and ipilimumab (NCT02648997). These trials highlight the ongoing efforts evaluating a meaningful anti-tumor T-cell response as an attractive therapeutic option for patients with meningioma.

This atlas of meningioma T-cell antigens can be used to design vaccines using the antigen (mRNA, protein, and peptide), soluble bispecific constructs as T-cell engagers, or for cellular therapies such as peptide-pulsed dendritic cells or ex vivo expanded antigen-specific T cells for both patient-individualized and off-the-shelf approaches.

## Supplementary Information

Below is the link to the electronic supplementary material.

Supplementary file1 (DOCX 288 kb)

Supplementary file2 (DOCX 2970 kb)
