# Supplementary material for: A T-cell antigen atlas for meningioma: novel options for immunotherapy
Source: Acta Neuropathol. 2023 Jun 27;146(2):173–90. doi: 10.1007/s00401-023-02605-w (PMC10329067; doi:10.1007/s00401-023-02605-w)
Supplement: Supplementary file 1 — Supplementary file1 (DOCX 288 kb) [file 401_2023_2605_MOESM1_ESM.docx]

**Supplementary Table 1** Clinical metadata of the 33 meningioma patients included in the study. Besides the basic patient characteristics, histology, location and maximal tumor diameter are displayed

| **Sample ID** | **WHO grade** | **Histology** | **Sex** | **Age at surgery** | **Location** | **Maximal tumor diameter [cm]** |
| --- | --- | --- | --- | --- | --- | --- |
| 1 | II | atypical | female | 73 | falx | 4.0 |
| 2 | I | meningothelial | female | 57 | skull base | 4.3 |
| 3 | I | transitional or mixed | female | 46 | falx | 3.7 |
| 4 | I | transitional or mixed | female | 81 | skull base | 5.6 |
| 5 | I | meningothelial | female | 43 | skull base | 2.6 |
| 6 | I | fibrous or fibroblastic | female | 67 | skull base | 4.6 |
| 7 | I | meningothelial | female | 78 | skull base | 3.1 |
| 499 | I | microcystic | male | 69 | convexity | 4.3 |
| 501 | II | atypical | female | 72 | convexity | 3.5 |
| 612 | III | anaplastic | female | 46 | convexity | 2.4 |
| 623 | I | transitional or mixed | female | 64 | falx | 1.7 |
| 624 | I | fibrous or fibroblastic | female | 73 | convexity | 3.5 |
| 628 | II | atypical | male | 65 | convexity | 3.0 |
| 632 | I | microcystic | male | 42 | convexity | 7.0 |
| 634 | I | meningothelial | male | 56 | skull base | 5.1 |
| 635 | II | atypical | male | 59 | skull base | 8.0 |
| 636 | I | transitional or mixed | female | 59 | skull base | 3.7 |
| 637 | I | transitional or mixed | female | 37 | convexity | 3.1 |
| 638 | II | atypical | female | 58 | convexity | 6.0 |
| 641 | II | atypical | female | 59 | convexity | 4.5 |
| 642 | II | atypical | male | 56 | falx | 6.8 |
| 646 | I | meningothelial | female | 68 | intraosseous | 8.3 |
| 661 | I | transitional or mixed | male | 52 | convexity | 3.3 |
| 666 | I | transitional or mixed | female | 77 | skull base | 7.6 |
| 673 | I | transitional or mixed | female | 44 | convexity | 7.7 |
| 679 | I | meningothelial | female | 49 | skull base | 4.3 |
| 682 | I | not otherwise specified | female | 72 | convexity | 5 |
| 700 | I | meningothelial | male | 45 | convexity | 3.2 |
| 702 | I | meningothelial | male | 41 | convexity | 8.2 |
| 734 | III | anaplastic | male | 54 | convexity | 5.1 |
| 814 | II | atypical | male | 83 | convexity | 4 |
| 819 | I | meningothelial | female | 70 | convexity | 6.3 |
| 833 | II | atypical | male | 63 | convexity | 5.3 |

**Supplementary Table 2** Clinical and experimental metadata of the 33 meningioma patients included in the present study. Basic patient characteristics, WHO grade, HLA typing, and sample mass subjected to peptidome analysis. Nine meningiomas with available autologous tumor-free dura are marked in grey

| **Internal sample ID** | **Sex**  **Age of onset [years]** | **WHO grade** | **HLA typing** | **Sample mass HLA-IP [mg]** |
| --- | --- | --- | --- | --- |
| MNG1  Tissue from 1^st^ recurrence at 4.7 years after initial diagnosis | ♀  68 | II | A*24:02;A*29:02;B*18:01;B*44:03;C*16:01;C*07:01 | 1075 |
| MNG2 | ♀  57 | I | A*30:01;A*03:01;B*13:02;B*07:02;C*07:02;C*06:02 | 684 |
| MNG3 | ♀  46 | I | A*01:01;A*68:01;B*40:01;B*57:01;C*03:04;C*06:02 | 1002 |
| MNG4 | ♀  81 | I | A*01:01;A*03:01;B*07:02;B*51:08;C*16:02;C*07:02 | 3683 |
| MNG5 | ♀  43 | I | A*01:01;A*24:02;B*08:01;B*13:02;C*06:02;C*07:01 | 1275 |
| MNG6 | ♀  67 | I | A*02:01;A*68:01;B*18:01;B*35:03;C*04:01;C*07:01 | 1536 |
| MNG7 | ♀  78 | I | A*30:02;A*01:01;B*08:01;B*07:02;C*07:01;C*07:01 | 1530 |
| MNG499 | ♂  69 | I | A*11:01;A*68:01;B*51:01;B*51:02;C*15:02;C*15:02  DRB1*04:04;DRB1*04:04;DRB4*01:01;DRB4*01:01;  DQB1*03:02;DQB1*03:02;DQA1*03:01;DQA1*03:01 | 1827 |
| MNG501 | ♀  72 | II | A*02:01;A*02:01;B*15:01;B*51:01;C*03:03;C*15:02  DRB1*13:01;DRB1*15:01;DRB3*02:02;DRB5*01:01;  DQB1*06:02;DQB1*06:03;DQA1*01:03;DQA1*01:02 | 1355 |
| MNG612  Tissue from 2^nd^ recurrence at 10.9 years after initial diagnosis | ♀  34 | III | A*32:01;A*02:01;B*51:01;B*27:05;C*14:02;C*01:02 | 4858 |
| MNG623  MNG623 Dura | ♀  64 | I | A*29:02;A*31:01;B*18:01;B*44:03;C*07:01;C*16:01  DRB1*07:01;DRB1*11:04;DRB3*02:02;DRB4*01:01;  DQB1*02:01;DQB1*03:01;DQA1*02:01;DQA1*05:01 | 522  300 |
| MNG624 | ♀  74 | I | A*01:01;A*01:01;B*08:01;B*35:01;C*04:01;C*07:01  DRB1*01:01;DRB1*03:01;DRB3*01:01;DQB1*02:01;  DQB1*05:01;DQA1*01:01;DQA1*05:01 | 581 |
| MNG628  MNG628 Dura | ♂  65 | II | A*02:01;A*24:02;B*18:01;B*35:01;C*04:01;C*12:03  DRB1*11:04;DRB1*14:01;DRB3*02:01;DRB3*02:02;  DQB1*03:01;DQB1*05:03;DQA1*01:01;DQA1*05:01 | 1167  473 |
| MNG632 | ♂  42 | I | A*30:02;A*68:01;B*35:01;B*35:01;C*04:01;C*04:01 | 1637 |
| MNG634  Tissue from 1^st^ recurrence at 7.3 years after initial diagnosis | ♂  49 | I | A*24:02;A*31:01;B*13:02;B*55:01;C*06:02;C*01:02 | 915 |
| MNG635 | ♀  59 | II | A*24:02;A*31:01;B*13:02;B*55:01;C*06:02;C*01:02 | 249 |
| MNG636 | ♀  59 | I | A*03:01;A*33:01;B*38:01;B*44:03;C*02:02;C*12:03 | 1613 |
| MNG637 | ♀  37 | I | A*02:01;A*02:01;B*18:01;B*55:01;C*03:03;C*12:03 | 3021 |
| MNG638 | ♀  57 | II | A*24:02;A*02:01;B*18:01;B*14:02;C*08:02;C*07:01 | 1171 |
| MNG641 | ♀  59 | II | A*02:01;A*03:01;B*07:02;B*41:01;C*07:02;C*17:01  DRB1*11:04;DRB1*16:01;DRB3*02:02;DRB5*02:02;  DQB1*03:01;DQB1*05:02;DQA1*01:02;DQA1*05:01 | 3372 |
| MNG642 | ♂  56 | II | A*11:01;A*34:01;B*15:35;B*51:01;C*04:01;C*07:02  DRB1*04:05;DRB1*15:02;DRB4*01:01;DRB5*01:01;  DQB1*04:01;DQB1*05:02;DQA1*01:02;DQA1*03:01 | 1769 |
| MNG646 | ♀  63 | I | A*01:01;A*03:01;B*08:01;B*40:02;C*02:02;C*07:01  DRB1*03:01;DRB1*04:01;DRB3*01:01;DRB4*01:01;  DQB1*02:01;DQB1*03:02;DQA1*03:01;DQA1*05:01 | 3025 |
| MNG661  MNG661 Dura | ♂  42 | I | A*24:02;A*30:02;B*18:01;B*49:01;C*03:03;C*12:03  DRB1*04:05;DRB1*11:04;DRB3*02:02;DRB4*01:01; DQB1*03:01;DQB1*03:02;DQA1*03:01;DQA1*05:01 | 1454  974 |
| MNG666 | ♀  67 | I | A*02:01;A*03:01;B*07:02;B*39:01;C*07:02;C*12:03  DRB1*11:01;DRB1*13:01;DRB3*02:02;DRB3*02:02;  DQB1*03:01;DQB1*06:03;DQA1*01:03;DQA1*05:01 | 2339 |
| MNG673 | ♀  44 | I | A*02:01;A*29:02;B*44:03;B*57:01;C*03:04;C*16:01  DRB1*07:01;DRB1*13:01;DRB3*02:02;DRB4*01:01;  DQB1*02:01;DQB1*06:03;DQA1*01:03;DQA1*02:01 | 2658 |
| MNG679  MNG679 Dura | ♀  49 | I | A*23:01;A*29:02;B*44:03;B*44:03;C*04:01;C*16:01  DRB1*07:01;DRB1*07:01;DRB4*01:01;DRB4*01:01;  DQB1*02:01;DQB1*02:01;DQA1*02:01;DQA1*02:01 | 1135  605 |
| MNG682 | ♀  72 | I | A*26:01;A*29:02;B*15:01;B*35:01;C*04:01;C*04:01  DRB1*01:01;DRB1*14:01;DRB3*02:02;DQB1*05:03; DQB1*05:01;DQA1*01:01;DQA1*01:01 | 1346 |
| MNG700  MNG700 Dura | ♂  44 | I | A*02:01;A*32:01;B*27:05;B*44:02;C*02:02;C*05:01  DRB1*04:01;DRB1*09:01;DRB4*01:01;DRB4*01:01;  DQB1*03:01;DQB1*03:03;DQA1*03:01;DQA1*03:01 | 874  380 |
| MNG702  MNG702 Dura | ♂  41 | I | A*01:01;A*02:01;B*37:01;B*51:01;C*02:02;C*06:02  DRB1*11:01;DRB1*13:01;DRB3*01:01;DRB3*02:02;  DQB1*03:01;DQB1*06:03;DQA1*01:03;DQA1*05:01 | 1441  2355 |
| MNG734 | ♂  54 | III | A*02:01;A*66:01;B*39:31;B*40:02;C*02:02;C*12:03  DRB1*12:01;DRB1*16:02;DRB3*02:02;DRB5*02:02;  DQB1*03:01;DQB1*05:02;DQA1*01:02;DQA1*05:01 | 1259 |
| MNG814  MNG814 Dura | ♂  83 | II | A*03:01;A*68:01;B*07:02;B*07:02;C*07:02;C*07:02  DRB1*14:01;DRB1*04:04;DRB3*02:02;DRB4*01:01;  DQB1*03:02;DQB1*05:03;DQA1*01:01;DQA1*03:01 | 896  1324 |
| MNG819  MNG819 Dura | ♀  70 | I | A*02:01;A*23:01;B*15:01;B*50:01;C*03:03;C*06:02  DRB1*03:01;DRB1*04:01;DRB3*02:02;DRB4*01:01;  DQB1*02:01;DQB1*03:02;DQA1*03:01;DQA1*05:01 | 1543  988 |
| MNG833  MNG833 Dura | ♂  63 | II | A*24:02;A*24:02;B*35:01;B*51:01;C*01:02;C*04:01  DRB1*11:01;DRB1*13:01;DRB3*02:02;DRB3*02:02;  DQB1*03:01;DQB1*06:03;DQA1*01:03;DQA1*05:01 | 1735  1689 |

**Supplementary Table 3** Percentages of cytotoxic T cells (CD8^+^), T helper cells (CD4^+^) and regulatory T cells (CD3^+^FoxP3^+^) are shown for the three WHO grades in separate columns. Staining was measured quantitatively using QuPath v0.4.3. Each patient/sample is represented by two tissue pieces as replicates and the replicates of one patient are surrounded by a black box. Replicates of patients that are part of the HLA ligandome cohort are surrounded by a red box and labeled separately

|  |  |  |  |  |  |  |  |  |  |  |  |  |  |
| --- | --- | --- | --- | --- | --- | --- | --- | --- | --- | --- | --- | --- | --- |
| **WHO Grade I** | | |  |  | **WHO Grade II** | | |  |  | **WHO Grade III** | | |  |
| **CD4^+^** | **CD8^+^** | **CD3^+^FoxP3^+^** |  |  | **CD4^+^** | **CD8^+^** | **CD3^+^FoxP3^+^** |  |  | **CD4^+^** | **CD8^+^** | **CD3^+^FoxP3^+^** |  |
| 22.0 | 1.5 | 0.0 |  |  | 0.1 | 0.2 | 0.0 |  |  | 3.6 | 0.0 | 0.0 |  |
| 28.1 | 4.6 | 0.0 |  |  | 0.5 | 0.1 | 0.0 |  |  | 4.4 | 0.2 | 0.0 |  |
| 6.1 | 1.0 | 0.0 |  |  | 0.0 | 0.0 | 0.0 |  |  | 7.2 | 0.1 | 0.0 |  |
| 0.7 | 0.7 | 0.0 |  |  | 0.2 | 0.5 | 0.0 |  |  | 6.9 | 0.5 | 0.1 |  |
| 17.6 | 0.4 | 0.0 |  |  | 14.9 | 0.8 | 0.0 |  |  | 0.1 | 0.0 | 0.0 |  |
| 24.4 | 0.2 | 0.0 |  |  | 9.9 | 0.2 | 0.0 |  |  | 0.2 | 0.0 | 0.0 |  |
| 27.8 | 0.7 | 0.0 |  |  | 8.8 | 0.9 | 0.0 |  |  | NaN | 0.0 | NaN |  |
| 30.8 | NaN | NaN |  |  | 6.0 | 0.6 | 0.0 |  |  | 18.0 | 0.1 | 0.0 |  |
| 4.5 | 0.8 | 0.0 |  |  | 1.3 | 0.0 | 0.0 |  |  | 0.7 | 0.0 | 0.0 |  |
| 6.3 | 0.5 | 0.0 |  |  | 2.0 | 0.0 | 0.0 |  |  | 1.6 | 0.0 | 0.0 |  |
| 8.1 | 0.2 | 0.1 |  |  | 2.1 | 2.2 | 0.0 |  |  | 0.4 | 0.1 | 0.0 |  |
| 15.4 | 0.3 | 0.0 |  |  | 0.8 | 1.8 | 0.0 |  |  | 0.2 | 0.0 | 0.0 |  |
| 0.8 | 0.3 | 0.0 |  |  | 0.8 | 0.1 | 0.0 |  |  | 41.1 | 1.2 | 0.0 |  |
| 0.9 | 1.3 | 0.0 |  |  | 3.0 | 0.0 | 0.0 |  |  | 67.0 | 2.2 | 0.0 |  |
| 17.2 | 0.3 | 0.0 |  |  | 0.0 | 0.1 | 0.0 |  |  | 57.3 | 0.4 | 0.1 | 734 |
| 2.7 | 0.3 | 0.0 |  |  | 0.0 | 0.1 | 0.0 |  |  | 38.3 | 0.2 | 0.0 |  |
| 1.6 | 0.1 | 0.0 |  |  | 0.9 | 0.0 | 0.0 |  |  | 25.0 | 0.2 | 0.0 |  |
| 1.9 | 0.0 | 0.0 |  |  | 0.9 | 0.0 | 0.0 |  |  | 18.4 | 0.1 | 0.0 |  |
| 21.3 | 2.9 | 0.0 |  |  | 3.0 | 0.0 | 0.0 | 635 |  | 56.9 | 0.9 | 0.1 |  |
| 8.9 | 2.0 | 0.0 |  |  | 5.1 | 0.0 | 0.0 |  |  | 61.1 | 0.2 | NaN |  |
| 4.8 | 0.3 | 0.0 |  |  | 11.8 | 0.0 | 0.0 | 1 |  |  |  |  |  |
| 5.7 | 0.3 | 0.0 |  |  | 0.1 | 0.0 | 0.0 |  |  |  |  |  |  |
| 5.3 | 0.7 | 0.0 | 6 |  | 0.7 | 0.0 | 0.0 |  |  |  |  |  |  |
| 23.4 | 0.5 | 0.0 |  |  | 0.5 | 0.0 | 0.0 |  |  |  |  |  |  |
| 8.4 | 0.1 | 0.0 | 632 |  | 4.8 | 0.0 | 0.0 |  |  |  |  |  |  |
| 15.7 | 0.7 | 0.0 |  |  | 0.1 | 0.0 | 0.0 |  |  |  |  |  |  |
| 3.8 | NaN | 0.0 |  |  | 13.4 | 0.0 | 0.0 |  |  |  |  |  |  |
| NaN | NaN | NaN |  |  | 8.5 | 0.4 | 0.0 |  |  |  |  |  |  |
| NaN | NaN | NaN |  |  | 1.2 | 0.0 | 0.0 |  |  |  |  |  |  |
| 38.6 | 1.6 | 0.0 |  |  | 10.7 | 0.0 | 0.0 |  |  |  |  |  |  |
| 1.6 | 0.0 | 0.0 |  |  | 1.5 | 0.0 | 0.0 |  |  |  |  |  |  |
| 1.6 | 0.4 | 0.0 |  |  | 5.0 | 0.0 | 0.0 |  |  |  |  |  |  |
| 6.3 | 1.4 | 0.0 | 5 |  | 2.9 | 0.0 | 0.0 |  |  |  |  |  |  |
| 1.7 | 0.1 | 0.0 |  |  | 8.3 | 0.3 | 0.0 |  |  |  |  |  |  |
| 9.8 | 2.5 | 0.0 | 634 |  | 0.3 | 0.2 | 0.0 |  |  |  |  |  |  |
| 14.0 | 1.5 | 0.0 |  |  | 0.3 | 0.3 | 0.0 |  |  |  |  |  |  |
| 6.7 | 1.2 | 0.0 |  |  | 2.0 | 0.3 | 0.0 |  |  |  |  |  |  |
| 11.9 | 3.1 | 0.0 |  |  | 4.4 | 0.1 | 0.0 |  |  |  |  |  |  |
| 6.9 | 0.1 | 0.0 |  |  | 1.8 | 0.0 | 0.0 |  |  |  |  |  |  |
| 6.8 | 0.7 | 0.0 |  |  | 1.4 | 0.1 | 0.0 |  |  |  |  |  |  |
| 5.4 | 0.1 | 0.0 |  |  | 0.2 | 0.0 | 0.0 |  |  |  |  |  |  |
| 23.1 | 0.6 | 0.0 |  |  | 0.1 | 0.0 | 0.0 |  |  |  |  |  |  |
| 9.3 | 0.5 | 0.0 |  |  | 0.5 | 0.0 | 0.0 |  |  |  |  |  |  |
| 1.5 | 0.1 | 0.0 |  |  | 0.1 | 0.1 | 0.0 |  |  |  |  |  |  |
| 66.2 | 4.2 | 0.0 |  |  | 0.4 | 0.0 | 0.0 |  |  |  |  |  |  |
| 48.5 | 3.8 | 0.0 |  |  | 0.5 | 0.0 | 0.0 |  |  |  |  |  |  |
| 9.0 | 5.6 | 0.0 |  |  | 7.8 | 0.0 | 0.0 |  |  |  |  |  |  |
| 4.8 | 0.8 | 0.0 |  |  | 2.0 | 0.0 | 0.0 |  |  |  |  |  |  |
| 8.3 | 0.2 | 0.0 |  |  | 2.9 | 0.7 | 0.0 |  |  |  |  |  |  |
| 1.8 | 0.3 | 0.0 |  |  | 4.5 | 2.4 | 0.0 |  |  |  |  |  |  |
| 21.8 | 0.5 | 0.0 |  |  | 9.6 | 0.0 | 0.1 |  |  |  |  |  |  |
| 23.2 | 0.3 | 0.0 |  |  | 7.8 | 0.1 | 0.0 |  |  |  |  |  |  |
| 9.2 | 0.0 | 0.0 | 636 |  | 6.2 | 0.0 | 0.0 |  |  |  |  |  |  |
| 19.4 | 0.2 | 0.0 |  |  | 2.5 | 0.1 | 0.0 |  |  |  |  |  |  |
| 15.0 | 0.9 | 0.0 | 4 |  | 0.4 | 0.0 | 0.0 |  |  |  |  |  |  |
| 6.8 | 0.5 | 0.0 |  |  | 0.2 | 0.0 | 0.0 |  |  |  |  |  |  |
| 2.2 | 0.7 | 0.0 |  |  | 0.2 | 0.0 | 0.0 |  |  |  |  |  |  |
| 6.4 | 0.4 | 0.0 |  |  | 0.7 | 0.0 | 0.0 |  |  |  |  |  |  |
| NaN | NaN | NaN | 3 |  | 4.4 | 0.0 | 0.0 |  |  |  |  |  |  |
| 4.1 | 0.0 | 0.0 |  |  | 3.8 | 0.0 | 0.0 |  |  |  |  |  |  |
| 1.1 | 1.1 | 0.0 |  |  | 5.6 | 0.0 | 0.0 |  |  |  |  |  |  |
| 1.4 | 0.5 | 0.0 |  |  | 8.9 | 0.3 | 0.1 |  |  |  |  |  |  |
| 6.1 | 0.0 | 0.0 | 637 |  | 1.1 | 0.0 | 0.0 |  |  |  |  |  |  |
| 0.7 | 0.0 | 0.0 |  |  | 0.7 | 0.0 | 0.0 |  |  |  |  |  |  |
| 99.6 | 1.1 | 0.0 |  |  | 4.8 | 0.3 | 0.0 |  |  |  |  |  |  |
| 91.5 | 3.8 | 0.0 |  |  | 8.2 | 0.1 | 0.0 |  |  |  |  |  |  |
| 22.9 | 0.2 | 0.0 |  |  | 0.7 | 0.1 | 0.0 |  |  |  |  |  |  |
| 14.2 | 0.1 | 0.0 |  |  | 0.8 | 0.0 | 0.0 |  |  |  |  |  |  |
| 2.6 | 2.9 | 0.0 |  |  | 3.3 | 1.7 | 0.0 |  |  |  |  |  |  |
| 1.7 | 1.9 | 0.0 |  |  | 0.6 | 0.1 | 0.0 |  |  |  |  |  |  |
| 8.2 | 0.5 | 0.0 |  |  | 4.0 | 0.0 | 0.0 |  |  |  |  |  |  |
| 7.2 | 0.5 | 0.0 |  |  | 4.7 | 0.3 | 0.0 |  |  |  |  |  |  |
| 88.6 | 1.9 | NaN |  |  | 0.8 | 0.0 | 0.0 |  |  |  |  |  |  |
| 87.7 | 1.5 | 0.0 |  |  | 1.7 | 0.3 | 0.0 |  |  |  |  |  |  |
| 7.6 | 0.4 | 0.0 |  |  | 2.5 | 0.1 | 0.0 |  |  |  |  |  |  |
| 10.4 | 1.3 | 0.0 |  |  | 42.0 | 0.7 | 0.0 |  |  |  |  |  |  |
| 18.9 | 1.2 | 0.0 |  |  | 1.1 | 0.1 | 0.0 |  |  |  |  |  |  |
| 8.5 | 0.3 | 0.0 |  |  | 1.8 | 0.0 | 0.0 |  |  |  |  |  |  |
| 17.7 | 0.1 | 0.0 |  |  | 2.3 | 0.1 | 0.0 |  |  |  |  |  |  |
| 18.6 | 0.0 | 0.0 |  |  | 4.8 | 0.0 | 0.3 |  |  |  |  |  |  |
| 8.0 | 0.6 | 0.0 |  |  | 24.7 | 0.6 | 0.0 |  |  |  |  |  |  |
| 0.2 | 0.0 | 0.0 |  |  | 13.4 | 0.9 | 0.0 |  |  |  |  |  |  |
| 1.2 | 0.2 | 0.0 |  |  | 22.4 | 0.0 | 0.0 |  |  |  |  |  |  |
| 3.4 | 0.6 | 0.0 |  |  | NaN | 0.4 | NaN |  |  |  |  |  |  |
| 32.1 | 0.8 | 0.0 |  |  | 18.9 | 0.2 | 0.0 |  |  |  |  |  |  |
| 31.9 | 0.7 | 0.0 |  |  | 9.6 | 0.2 | 0.0 |  |  |  |  |  |  |
| 9.1 | 0.7 | 0.0 |  |  | 2.4 | 0.1 | 0.0 |  |  |  |  |  |  |
| 16.0 | 0.5 | 0.0 |  |  | 0.8 | 0.0 | 0.0 |  |  |  |  |  |  |
| 9.5 | 0.2 | 0.0 |  |  | 6.1 | 0.5 | 0.0 |  |  |  |  |  |  |
| 13.6 | 0.3 | 0.0 |  |  | 7.9 | 0.4 | 0.0 |  |  |  |  |  |  |
| 0.9 | 0.0 | 0.0 |  |  | 12.0 | 0.5 | 0.0 |  |  |  |  |  |  |
| 2.2 | 0.2 | 0.0 |  |  | 13.8 | 0.0 | 0.0 |  |  |  |  |  |  |
| 0.4 | 0.2 | 0.0 |  |  | 7.9 | 1.0 | 0.0 |  |  |  |  |  |  |
| 0.1 | 0.1 | 0.0 |  |  | 2.1 | 0.2 | 0.0 |  |  |  |  |  |  |
| 36.8 | 0.4 | 0.0 |  |  | 0.1 | 0.1 | 0.0 |  |  |  |  |  |  |
| 25.6 | 0.3 | 0.0 |  |  | 0.1 | 0.0 | 0.0 |  |  |  |  |  |  |
| 0.8 | 1.1 | 0.0 |  |  | 11.5 | 0.6 | 0.0 |  |  |  |  |  |  |
| 1.9 | 0.8 | 0.0 |  |  | 2.7 | 0.4 | 0.0 |  |  |  |  |  |  |
| 10.8 | 0.3 | 0.0 |  |  | 17.1 | 0.8 | 0.0 |  |  |  |  |  |  |
| 9.4 | 0.2 | 0.0 |  |  | 5.6 | 0.2 | 0.0 |  |  |  |  |  |  |
| 6.9 | 0.0 | 0.0 |  |  | 16.9 | 0.2 | 0.0 |  |  |  |  |  |  |
| 3.6 | 0.1 | 0.0 |  |  | 12.6 | 1.1 | 0.2 |  |  |  |  |  |  |
| 3.9 | 0.4 | 0.0 |  |  | 8.5 | 0.4 | 0.0 | 628 |  |  |  |  |  |
| 4.9 | 0.5 | 0.0 |  |  | 10.5 | 2.3 | 0.0 |  |  |  |  |  |  |
| 8.4 | 0.1 | 0.0 |  |  | 3.9 | 0.4 | 0.1 |  |  |  |  |  |  |
| 4.3 | 0.2 | 0.0 |  |  | 6.7 | 0.3 | 0.1 |  |  |  |  |  |  |
| 34.0 | 0.2 | 0.0 |  |  | 1.9 | 0.0 | 0.1 | 641 |  |  |  |  |  |
| 29.9 | 0.1 | 0.0 |  |  | 2.1 | 0.1 | 0.0 |  |  |  |  |  |  |
| 3.2 | 0.1 | 0.0 |  |  | 3.1 | 0.2 | 0.0 |  |  |  |  |  |  |
| 4.8 | 0.2 | 0.0 |  |  | 1.1 | 0.0 | 0.0 |  |  |  |  |  |  |
| 11.9 | 0.1 | 0.0 |  |  | 0.9 | 0.0 | 0.0 |  |  |  |  |  |  |
| 9.6 | 0.4 | 0.0 |  |  | 1.4 | 0.1 | 0.0 |  |  |  |  |  |  |
| 76.7 | 0.4 | 0.0 |  |  | 0.3 | 0.0 | 0.0 |  |  |  |  |  |  |
| 76.7 | 2.3 | 0.0 |  |  | 1.2 | 0.0 | 0.0 |  |  |  |  |  |  |
| 16.8 | 0.5 | 0.0 |  |  | 0.5 | 0.2 | 0.0 |  |  |  |  |  |  |
| 46.6 | 1.7 | 0.0 |  |  | 0.9 | 0.2 | 0.0 |  |  |  |  |  |  |
| 6.6 | 2.5 | 0.0 |  |  | 0.5 | 0.0 | 0.0 |  |  |  |  |  |  |
| 14.9 | 5.3 | 0.0 |  |  | 0.3 | 0.0 | 0.0 |  |  |  |  |  |  |
| 21.0 | 2.4 | 0.0 |  |  | 6.5 | 0.2 | 0.0 |  |  |  |  |  |  |
| 6.5 | 1.7 | 0.0 |  |  | 7.5 | 0.1 | 0.0 |  |  |  |  |  |  |
| 0.3 | 0.4 | 0.0 |  |  | 0.5 | 0.1 | 0.0 |  |  |  |  |  |  |
| 0.5 | 1.0 | 0.0 |  |  | 1.4 | 0.4 | 0.0 |  |  |  |  |  |  |
| NaN | 0.1 | 0.0 |  |  | 3.5 | 0.0 | 0.0 |  |  |  |  |  |  |
| NaN | 0.9 | 0.0 |  |  | 13.3 | 1.0 | 0.6 |  |  |  |  |  |  |
| 36.6 | NaN | 0.0 |  |  | 4.0 | 0.0 | 0.0 |  |  |  |  |  |  |
| 37.5 | 2.1 | 0.0 |  |  | 3.2 | 0.0 | 0.0 |  |  |  |  |  |  |
| 7.3 | 0.5 | 0.0 |  |  | 6.1 | 0.0 | 0.0 |  |  |  |  |  |  |
| 5.5 | 0.5 | 0.0 |  |  | 6.0 | 0.4 | 0.0 |  |  |  |  |  |  |
| 10.2 | 0.8 | 0.0 |  |  | 16.8 | 0.0 | 0.0 |  |  |  |  |  |  |
| 18.9 | 1.4 | 0.0 |  |  | 27.0 | 0.1 | 0.0 |  |  |  |  |  |  |
| 10.4 | 0.0 | 0.0 |  |  | 1.8 | 0.1 | 0.0 |  |  |  |  |  |  |
| 13.5 | 0.2 | 0.0 |  |  | 17.3 | 0.3 | 0.3 |  |  |  |  |  |  |
| 0.6 | 0.1 | 0.0 |  |  | 1.2 | 0.0 | 0.0 |  |  |  |  |  |  |
| 2.8 | 0.1 | 0.0 |  |  | 16.2 | 0.3 | 0.1 |  |  |  |  |  |  |
| 29.6 | 0.4 | 0.0 |  |  | 4.9 | 0.5 | 0.0 |  |  |  |  |  |  |
| 34.9 | 0.0 | 0.0 |  |  | 17.8 | 0.8 | 0.0 |  |  |  |  |  |  |
| 0.8 | 0.3 | 0.0 |  |  | 17.4 | 2.2 | 0.1 |  |  |  |  |  |  |
| 4.0 | 1.0 | 0.0 |  |  | 10.1 | 1.4 | 0.0 |  |  |  |  |  |  |
| 1.5 | 0.2 | 0.0 |  |  | 1.9 | 0.9 | 0.0 |  |  |  |  |  |  |
| 0.7 | 0.0 | 0.0 |  |  | 7.7 | 1.3 | 0.0 |  |  |  |  |  |  |
| 3.6 | 0.0 | 0.0 |  |  | 14.2 | 0.2 | 0.1 |  |  |  |  |  |  |
| 16.2 | 0.1 | 0.0 |  |  | 47.3 | 0.3 | 0.3 |  |  |  |  |  |  |
| 3.7 | 0.3 | 0.0 |  |  | 61.5 | 1.0 | 0.0 | 814 |  |  |  |  |  |
| 7.0 | 0.4 | 0.0 |  |  | 65.8 | 0.8 | 0.0 |  |  |  |  |  |  |
| 3.0 | 0.7 | 0.0 |  |  | 0.4 | 0.1 | 0.0 |  |  |  |  |  |  |
| 11.4 | 2.7 | 0.0 |  |  | 0.2 | 0.1 | 0.0 |  |  |  |  |  |  |
| 45.4 | 2.4 | 0.0 |  |  | 46.0 | 6.6 | 0.0 |  |  |  |  |  |  |
| 47.0 | NaN | 0.0 |  |  | 51.3 | 1.0 | 0.0 |  |  |  |  |  |  |
| 1.1 | 0.3 | 0.0 |  |  | 32.0 | 0.2 | 0.0 |  |  |  |  |  |  |
| 3.7 | 1.4 | 0.0 |  |  | 45.1 | 0.6 | 0.0 |  |  |  |  |  |  |
| 10.5 | 0.1 | 0.0 |  |  | 31.6 | 0.1 | 0.0 | 833 |  |  |  |  |  |
| 35.5 | 0.1 | 0.0 |  |  | 16.0 | 0.3 | 0.0 |  |  |  |  |  |  |
| 1.1 | 0.2 | 0.0 |  |  | 1.1 | 0.0 | 0.0 |  |  |  |  |  |  |
| 0.8 | 0.3 | 0.0 |  |  | 0.2 | 0.0 | 0.0 |  |  |  |  |  |  |
| 0.1 | 0.0 | 0.0 |  |  | 26.2 | 0.1 | 0.0 |  |  |  |  |  |  |
| 1.2 | 0.0 | 0.0 |  |  | 0.2 | 0.0 | 0.0 |  |  |  |  |  |  |
| NaN | 0.0 | 0.0 |  |  | 3.6 | 0.0 | 0.0 |  |  |  |  |  |  |
| 3.4 | 0.0 | 0.0 |  |  | 11.4 | 0.2 | 0.0 |  |  |  |  |  |  |
| 13.6 | 0.8 | 0.0 |  |  | 64.0 | 0.1 | 0.0 |  |  |  |  |  |  |
| 20.7 | 1.5 | 0.0 |  |  | 4.3 | 0.0 | 0.0 |  |  |  |  |  |  |
| 13.5 | 2.3 | 0.0 |  |  | 21.9 | 1.4 | 0.1 |  |  |  |  |  |  |
| 19.8 | 3.9 | 0.0 |  |  | 90.8 | 5.2 | 0.2 |  |  |  |  |  |  |
| 14.4 | 0.3 | 0.0 |  |  | 9.6 | 0.0 | 0.0 |  |  |  |  |  |  |
| 41.8 | 0.4 | 0.0 |  |  | 14.9 | 0.1 | 0.0 |  |  |  |  |  |  |
| 4.9 | 1.2 | 0.0 | 499 |  | 25.8 | 0.2 | 0.1 |  |  |  |  |  |  |
| 2.4 | 0.4 | 0.0 |  |  | 15.6 | 0.0 | 0.0 |  |  |  |  |  |  |
| 9.8 | 1.0 | 0.0 |  |  | 5.3 | 0.0 | 0.0 |  |  |  |  |  |  |
| 17.5 | 3.1 | 0.0 |  |  | 57.1 | 1.8 | 0.0 |  |  |  |  |  |  |
| 5.9 | 0.5 | 0.0 |  |  |  |  |  |  |  |  |  |  |  |
| 21.8 | 0.1 | 0.0 |  |  |  |  |  |  |  |  |  |  |  |
| 0.9 | 0.4 | 0.0 |  |  |  |  |  |  |  |  |  |  |  |
| 3.7 | 0.4 | 0.0 |  |  |  |  |  |  |  |  |  |  |  |
| 5.3 | 0.5 | 0.0 |  |  |  |  |  |  |  |  |  |  |  |
| 8.9 | 0.0 | 0.0 |  |  |  |  |  |  |  |  |  |  |  |
| 0.3 | 0.5 | 0.0 |  |  |  |  |  |  |  |  |  |  |  |
| 0.9 | 0.1 | 0.0 |  |  |  |  |  |  |  |  |  |  |  |
| NaN | 5.4 | 0.0 |  |  |  |  |  |  |  |  |  |  |  |
| 22.7 | 3.9 | 0.0 |  |  |  |  |  |  |  |  |  |  |  |
| 70.8 | 0.3 | 0.0 |  |  |  |  |  |  |  |  |  |  |  |
| 77.4 | 0.4 | 0.0 |  |  |  |  |  |  |  |  |  |  |  |
| 0.3 | 0.2 | 0.0 |  |  |  |  |  |  |  |  |  |  |  |
| 3.7 | 0.6 | 0.0 |  |  |  |  |  |  |  |  |  |  |  |
| 5.1 | 0.6 | 0.0 |  |  |  |  |  |  |  |  |  |  |  |
| 2.1 | 0.6 | 0.0 |  |  |  |  |  |  |  |  |  |  |  |
| 4.4 | 0.2 | 0.0 |  |  |  |  |  |  |  |  |  |  |  |
| 18.6 | 0.7 | 0.0 |  |  |  |  |  |  |  |  |  |  |  |
| 0.8 | 0.2 | 0.0 |  |  |  |  |  |  |  |  |  |  |  |
| 2.0 | 0.2 | 0.0 |  |  |  |  |  |  |  |  |  |  |  |
| 11.5 | 6.1 | 0.0 |  |  |  |  |  |  |  |  |  |  |  |
| 2.9 | 2.3 | 0.0 |  |  |  |  |  |  |  |  |  |  |  |
| 47.0 | 1.8 | 0.0 |  |  |  |  |  |  |  |  |  |  |  |
| 38.5 | 0.3 | 0.0 |  |  |  |  |  |  |  |  |  |  |  |
| 1.1 | 0.4 | 0.0 |  |  |  |  |  |  |  |  |  |  |  |
| 4.5 | 1.1 | 0.0 |  |  |  |  |  |  |  |  |  |  |  |
| 38.5 | 0.2 | 0.0 |  |  |  |  |  |  |  |  |  |  |  |
| 54.3 | 0.5 | 0.0 |  |  |  |  |  |  |  |  |  |  |  |
| 10.6 | 0.0 | 0.0 |  |  |  |  |  |  |  |  |  |  |  |
| 10.9 | 0.1 | 0.0 |  |  |  |  |  |  |  |  |  |  |  |
| 23.2 | NaN | 0.0 |  |  |  |  |  |  |  |  |  |  |  |
| 10.5 | 4.0 | 0.0 |  |  |  |  |  |  |  |  |  |  |  |
| 1.2 | 0.5 | 0.0 |  |  |  |  |  |  |  |  |  |  |  |
| 2.4 | 0.6 | 0.0 |  |  |  |  |  |  |  |  |  |  |  |
| 0.2 | 0.1 | 0.0 |  |  |  |  |  |  |  |  |  |  |  |
| 0.3 | 0.0 | 0.0 |  |  |  |  |  |  |  |  |  |  |  |
| 2.3 | 0.6 | 0.0 |  |  |  |  |  |  |  |  |  |  |  |
| 4.7 | 0.9 | 0.0 |  |  |  |  |  |  |  |  |  |  |  |
| 19.9 | 0.1 | 0.0 |  |  |  |  |  |  |  |  |  |  |  |
| 37.9 | 0.0 | 0.0 |  |  |  |  |  |  |  |  |  |  |  |
| 15.1 | 0.1 | 0.0 |  |  |  |  |  |  |  |  |  |  |  |
| 6.7 | 0.4 | 0.0 |  |  |  |  |  |  |  |  |  |  |  |
| 15.6 | 0.1 | 0.0 |  |  |  |  |  |  |  |  |  |  |  |
| 9.0 | 0.2 | 0.0 |  |  |  |  |  |  |  |  |  |  |  |
| 4.9 | 3.4 | 0.0 |  |  |  |  |  |  |  |  |  |  |  |
| 3.9 | 0.7 | 0.0 |  |  |  |  |  |  |  |  |  |  |  |
| 2.3 | 0.6 | 0.0 |  |  |  |  |  |  |  |  |  |  |  |
| 5.3 | 0.2 | 0.0 |  |  |  |  |  |  |  |  |  |  |  |
| 67.9 | 0.5 | 0.0 |  |  |  |  |  |  |  |  |  |  |  |
| 69.8 | 0.5 | 0.0 |  |  |  |  |  |  |  |  |  |  |  |
| 25.7 | 0.0 | 0.0 |  |  |  |  |  |  |  |  |  |  |  |
| 28.0 | 0.1 | 0.0 |  |  |  |  |  |  |  |  |  |  |  |
| 3.4 | 0.3 | 0.0 |  |  |  |  |  |  |  |  |  |  |  |
| 11.5 | 0.1 | 0.0 |  |  |  |  |  |  |  |  |  |  |  |
| 9.2 | 2.1 | 0.0 |  |  |  |  |  |  |  |  |  |  |  |
| 9.5 | 2.3 | 0.0 |  |  |  |  |  |  |  |  |  |  |  |
| 9.2 | 0.0 | 0.0 |  |  |  |  |  |  |  |  |  |  |  |
| 14.2 | 0.1 | 0.0 |  |  |  |  |  |  |  |  |  |  |  |
| 64.4 | 0.1 | 0.0 |  |  |  |  |  |  |  |  |  |  |  |
| 64.9 | 0.1 | 0.0 |  |  |  |  |  |  |  |  |  |  |  |
| 32.7 | 0.0 | 0.0 |  |  |  |  |  |  |  |  |  |  |  |
| 23.5 | 0.0 | 0.0 |  |  |  |  |  |  |  |  |  |  |  |
| 3.8 | 0.1 | 0.0 |  |  |  |  |  |  |  |  |  |  |  |
| 3.4 | 0.1 | 0.0 |  |  |  |  |  |  |  |  |  |  |  |
| 44.8 | 0.0 | 0.0 |  |  |  |  |  |  |  |  |  |  |  |
| 47.5 | 0.2 | 0.0 |  |  |  |  |  |  |  |  |  |  |  |
| 0.4 | 0.0 | 0.0 |  |  |  |  |  |  |  |  |  |  |  |
| 0.8 | 0.0 | 0.0 |  |  |  |  |  |  |  |  |  |  |  |
| 3.4 | 0.2 | 0.0 |  |  |  |  |  |  |  |  |  |  |  |
| 1.0 | 0.6 | 0.0 |  |  |  |  |  |  |  |  |  |  |  |
| 24.3 | 0.2 | 0.0 |  |  |  |  |  |  |  |  |  |  |  |
| 12.9 | 0.2 | 0.0 |  |  |  |  |  |  |  |  |  |  |  |
| 11.8 | 0.4 | 0.0 |  |  |  |  |  |  |  |  |  |  |  |
| 12.5 | 0.6 | 0.0 |  |  |  |  |  |  |  |  |  |  |  |
| 11.0 | 0.0 | 0.0 |  |  |  |  |  |  |  |  |  |  |  |
| 12.4 | 0.1 | 0.0 |  |  |  |  |  |  |  |  |  |  |  |
| 5.9 | 0.6 | 0.0 |  |  |  |  |  |  |  |  |  |  |  |
| 7.4 | 0.6 | 0.0 |  |  |  |  |  |  |  |  |  |  |  |
| 8.8 | 0.0 | 0.0 |  |  |  |  |  |  |  |  |  |  |  |
| 10.6 | 0.1 | 0.0 |  |  |  |  |  |  |  |  |  |  |  |
| 3.9 | 0.4 | 0.0 |  |  |  |  |  |  |  |  |  |  |  |
| 14.2 | 0.4 | 0.0 |  |  |  |  |  |  |  |  |  |  |  |
| 37.5 | 0.2 | 0.0 |  |  |  |  |  |  |  |  |  |  |  |
| 11.5 | 0.1 | 0.0 |  |  |  |  |  |  |  |  |  |  |  |
| 4.4 | 0.2 | 0.0 |  |  |  |  |  |  |  |  |  |  |  |
| 5.6 | 0.2 | 0.0 |  |  |  |  |  |  |  |  |  |  |  |
| 1.9 | 0.0 | 0.0 |  |  |  |  |  |  |  |  |  |  |  |
| 24.4 | 0.6 | 0.0 |  |  |  |  |  |  |  |  |  |  |  |
| 0.0 | 0.9 | 0.0 |  |  |  |  |  |  |  |  |  |  |  |
| 10.9 | 0.7 | 0.0 |  |  |  |  |  |  |  |  |  |  |  |
| 8.8 | 2.0 | 0.0 |  |  |  |  |  |  |  |  |  |  |  |
| 14.2 | 2.7 | 0.0 |  |  |  |  |  |  |  |  |  |  |  |
| 46.6 | 0.2 | 0.0 |  |  |  |  |  |  |  |  |  |  |  |
| 6.6 | 0.4 | 0.0 |  |  |  |  |  |  |  |  |  |  |  |
| 5.4 | 0.4 | 0.0 |  |  |  |  |  |  |  |  |  |  |  |
| 13.7 | 1.5 | 0.0 |  |  |  |  |  |  |  |  |  |  |  |
| 1.2 | 0.5 | 0.0 |  |  |  |  |  |  |  |  |  |  |  |
| 1.7 | 0.1 | 0.0 |  |  |  |  |  |  |  |  |  |  |  |
| 32.5 | 0.1 | 0.0 |  |  |  |  |  |  |  |  |  |  |  |
| 24.0 | 0.1 | 0.0 |  |  |  |  |  |  |  |  |  |  |  |
| 17.3 | 0.2 | 0.0 |  |  |  |  |  |  |  |  |  |  |  |
| 18.7 | 0.6 | 0.0 |  |  |  |  |  |  |  |  |  |  |  |
| 54.5 | 3.3 | 0.0 |  |  |  |  |  |  |  |  |  |  |  |
| 46.8 | 1.5 | 0.0 |  |  |  |  |  |  |  |  |  |  |  |
| 11.3 | 1.1 | 0.0 |  |  |  |  |  |  |  |  |  |  |  |
| 5.8 | 0.3 | 0.0 |  |  |  |  |  |  |  |  |  |  |  |
| 8.1 | 0.5 | 0.0 |  |  |  |  |  |  |  |  |  |  |  |
| 8.4 | 0.2 | 0.0 |  |  |  |  |  |  |  |  |  |  |  |
| 50.0 | 0.3 | 0.0 |  |  |  |  |  |  |  |  |  |  |  |
| 71.6 | 0.5 | 0.0 |  |  |  |  |  |  |  |  |  |  |  |
| 13.5 | 0.3 | 0.0 |  |  |  |  |  |  |  |  |  |  |  |
| 10.0 | 0.1 | 0.0 |  |  |  |  |  |  |  |  |  |  |  |
| 4.8 | 0.0 | 0.0 |  |  |  |  |  |  |  |  |  |  |  |
| 46.2 | 0.3 | 0.0 |  |  |  |  |  |  |  |  |  |  |  |
| 27.0 | 1.5 | 0.0 |  |  |  |  |  |  |  |  |  |  |  |
| 44.0 | 1.0 | 0.0 |  |  |  |  |  |  |  |  |  |  |  |
| 8.7 | 1.6 | 0.0 |  |  |  |  |  |  |  |  |  |  |  |
| 15.9 | 1.5 | 0.0 |  |  |  |  |  |  |  |  |  |  |  |
| 53.0 | 1.4 | 0.0 |  |  |  |  |  |  |  |  |  |  |  |
| 21.5 | 1.5 | 0.0 |  |  |  |  |  |  |  |  |  |  |  |
| 40.1 | 1.1 | 0.0 |  |  |  |  |  |  |  |  |  |  |  |
| 46.4 | 2.2 | 0.0 |  |  |  |  |  |  |  |  |  |  |  |
| 49.3 | 3.7 | 0.0 |  |  |  |  |  |  |  |  |  |  |  |
| 46.0 | 2.8 | 0.0 |  |  |  |  |  |  |  |  |  |  |  |
| 7.4 | 0.1 | 0.0 |  |  |  |  |  |  |  |  |  |  |  |
| 18.5 | 0.1 | 0.0 |  |  |  |  |  |  |  |  |  |  |  |
| 17.4 | 1.2 | 0.0 |  |  |  |  |  |  |  |  |  |  |  |
| 2.3 | 1.4 | 0.0 |  |  |  |  |  |  |  |  |  |  |  |
| 17.0 | 2.3 | 0.0 |  |  |  |  |  |  |  |  |  |  |  |
| 17.4 | 6.0 | 0.0 |  |  |  |  |  |  |  |  |  |  |  |
| 1.4 | 0.6 | 0.0 |  |  |  |  |  |  |  |  |  |  |  |
| 18.3 | 1.3 | 0.0 |  |  |  |  |  |  |  |  |  |  |  |
| 18.8 | 2.2 | 0.0 |  |  |  |  |  |  |  |  |  |  |  |
| 18.8 | 2.9 | 0.0 |  |  |  |  |  |  |  |  |  |  |  |
| 12.4 | 0.1 | 0.0 |  |  |  |  |  |  |  |  |  |  |  |
| 11.6 | 0.4 | 0.0 |  |  |  |  |  |  |  |  |  |  |  |
| 85.0 | 0.0 | 0.0 |  |  |  |  |  |  |  |  |  |  |  |
| 88.2 | 0.4 | 0.0 |  |  |  |  |  |  |  |  |  |  |  |
| 28.8 | 0.1 | 0.0 |  |  |  |  |  |  |  |  |  |  |  |
| 31.2 | 0.2 | 0.0 |  |  |  |  |  |  |  |  |  |  |  |
| 13.4 | 1.1 | 0.0 |  |  |  |  |  |  |  |  |  |  |  |
| 3.7 | 0.1 | 0.0 |  |  |  |  |  |  |  |  |  |  |  |
| 45.9 | 0.5 | 0.0 |  |  |  |  |  |  |  |  |  |  |  |
| 45.5 | 1.1 | 0.0 |  |  |  |  |  |  |  |  |  |  |  |
| 21.6 | 0.9 | 0.0 |  |  |  |  |  |  |  |  |  |  |  |
| 20.0 | 0.8 | 0.0 |  |  |  |  |  |  |  |  |  |  |  |
| 8.3 | 5.4 | 0.0 |  |  |  |  |  |  |  |  |  |  |  |
| 16.8 | 0.8 | 0.0 |  |  |  |  |  |  |  |  |  |  |  |
| 25.1 | 2.5 | 0.0 |  |  |  |  |  |  |  |  |  |  |  |
| 14.3 | 2.7 | 0.0 |  |  |  |  |  |  |  |  |  |  |  |
| 13.6 | 2.4 | 0.0 |  |  |  |  |  |  |  |  |  |  |  |
| 8.3 | 0.3 | 0.0 |  |  |  |  |  |  |  |  |  |  |  |
| 2.1 | 0.8 | 0.0 |  |  |  |  |  |  |  |  |  |  |  |
| 25.4 | 0.4 | 0.0 |  |  |  |  |  |  |  |  |  |  |  |
| 73.7 | 1.5 | 0.0 |  |  |  |  |  |  |  |  |  |  |  |
| 73.1 | 1.2 | 0.0 |  |  |  |  |  |  |  |  |  |  |  |
| 21.3 | 0.1 | 0.0 |  |  |  |  |  |  |  |  |  |  |  |
| 27.6 | 0.3 | 0.0 |  |  |  |  |  |  |  |  |  |  |  |
| 5.7 | 0.8 | 0.0 |  |  |  |  |  |  |  |  |  |  |  |
| 58.7 | 14.6 | 0.0 |  |  |  |  |  |  |  |  |  |  |  |
| 2.8 | 0.5 | 0.0 |  |  |  |  |  |  |  |  |  |  |  |
| 1.9 | 0.2 | 0.0 |  |  |  |  |  |  |  |  |  |  |  |
| 1.1 | 0.2 | 0.0 |  |  |  |  |  |  |  |  |  |  |  |
| 4.8 | 0.9 | 0.0 |  |  |  |  |  |  |  |  |  |  |  |
| 30.0 | 0.4 | 0.0 |  |  |  |  |  |  |  |  |  |  |  |
| 3.3 | 0.2 | 0.0 |  |  |  |  |  |  |  |  |  |  |  |
| 17.8 | 1.7 | 0.0 |  |  |  |  |  |  |  |  |  |  |  |
| 27.3 | 0.9 | 0.0 |  |  |  |  |  |  |  |  |  |  |  |
| 26.5 | 2.7 | 0.0 |  |  |  |  |  |  |  |  |  |  |  |
| 12.8 | 1.1 | 0.0 |  |  |  |  |  |  |  |  |  |  |  |
| NaN | NaN | 0.0 |  |  |  |  |  |  |  |  |  |  |  |
| 80.0 | 1.2 | 0.0 |  |  |  |  |  |  |  |  |  |  |  |
| 36.6 | 0.0 | 0.0 |  |  |  |  |  |  |  |  |  |  |  |
| 23.4 | 0.1 | 0.0 |  |  |  |  |  |  |  |  |  |  |  |
| 20.1 | 1.7 | 0.0 |  |  |  |  |  |  |  |  |  |  |  |
| 8.6 | 1.0 | 0.0 |  |  |  |  |  |  |  |  |  |  |  |
| 61.9 | 0.2 | 0.0 |  |  |  |  |  |  |  |  |  |  |  |
| 26.2 | 0.5 | 0.0 |  |  |  |  |  |  |  |  |  |  |  |
| 39.2 | 0.1 | 0.0 |  |  |  |  |  |  |  |  |  |  |  |
| 76.2 | 0.2 | 0.0 |  |  |  |  |  |  |  |  |  |  |  |
| 4.6 | 0.2 | 0.0 |  |  |  |  |  |  |  |  |  |  |  |
| 4.3 | 0.0 | 0.0 |  |  |  |  |  |  |  |  |  |  |  |
| 57.3 | 0.7 | 0.0 |  |  |  |  |  |  |  |  |  |  |  |
| 53.3 | 2.8 | 0.0 |  |  |  |  |  |  |  |  |  |  |  |
| 23.1 | 0.1 | 0.0 |  |  |  |  |  |  |  |  |  |  |  |
| 23.2 | 0.1 | 0.0 |  |  |  |  |  |  |  |  |  |  |  |
| 45.2 | 1.2 | 0.0 |  |  |  |  |  |  |  |  |  |  |  |
| 45.0 | 0.4 | 0.0 |  |  |  |  |  |  |  |  |  |  |  |
| 91.6 | 0.8 | 0.0 |  |  |  |  |  |  |  |  |  |  |  |
| 60.6 | 1.7 | 0.0 |  |  |  |  |  |  |  |  |  |  |  |
| 87.5 | 2.0 | 0.0 |  |  |  |  |  |  |  |  |  |  |  |
| 87.3 | 2.9 | 0.0 |  |  |  |  |  |  |  |  |  |  |  |
| 35.7 | 0.6 | 0.0 |  |  |  |  |  |  |  |  |  |  |  |
| 47.8 | 0.0 | 0.0 |  |  |  |  |  |  |  |  |  |  |  |
| 8.0 | 3.1 | 0.0 |  |  |  |  |  |  |  |  |  |  |  |
| 6.3 | 2.9 | 0.0 |  |  |  |  |  |  |  |  |  |  |  |
| 18.6 | NaN | 0.0 |  |  |  |  |  |  |  |  |  |  |  |
| 14.6 | NaN | 0.0 |  |  |  |  |  |  |  |  |  |  |  |
| 1.9 | 0.0 | 0.0 |  |  |  |  |  |  |  |  |  |  |  |
| 9.8 | 0.1 | 0.0 |  |  |  |  |  |  |  |  |  |  |  |
| 46.8 | 0.5 | 0.0 |  |  |  |  |  |  |  |  |  |  |  |
| 27.2 | 1.3 | 0.0 |  |  |  |  |  |  |  |  |  |  |  |
| 26.7 | 0.1 | 0.0 |  |  |  |  |  |  |  |  |  |  |  |
| 17.5 | 0.6 | 0.0 |  |  |  |  |  |  |  |  |  |  |  |
| 5.4 | 0.0 | 0.0 |  |  |  |  |  |  |  |  |  |  |  |
| 8.0 | 0.2 | 0.0 |  |  |  |  |  |  |  |  |  |  |  |
| 11.7 | 0.9 | 0.0 |  |  |  |  |  |  |  |  |  |  |  |
| 6.8 | 1.2 | 0.0 |  |  |  |  |  |  |  |  |  |  |  |
| 64.3 | 0.0 | 0.0 |  |  |  |  |  |  |  |  |  |  |  |
| 45.3 | 0.0 | 0.0 |  |  |  |  |  |  |  |  |  |  |  |
| 7.6 | 0.0 | 0.0 |  |  |  |  |  |  |  |  |  |  |  |
| 5.1 | 0.0 | 0.0 |  |  |  |  |  |  |  |  |  |  |  |
| 36.1 | 1.4 | 0.0 |  |  |  |  |  |  |  |  |  |  |  |
| 29.2 | 1.0 | 0.0 |  |  |  |  |  |  |  |  |  |  |  |
| 0.7 | 2.1 | 0.0 | 624 |  |  |  |  |  |  |  |  |  |  |
| 1.5 | 0.7 | 0.0 |  |  |  |  |  |  |  |  |  |  |  |
| 6.7 | 0.1 | 0.0 |  |  |  |  |  |  |  |  |  |  |  |
| 4.4 | 0.0 | 0.0 |  |  |  |  |  |  |  |  |  |  |  |
| 0.5 | 0.0 | 0.0 |  |  |  |  |  |  |  |  |  |  |  |
| 2.0 | 0.0 | 0.0 |  |  |  |  |  |  |  |  |  |  |  |
| 0.1 | 0.0 | 0.0 |  |  |  |  |  |  |  |  |  |  |  |
| 0.5 | 0.0 | 0.0 |  |  |  |  |  |  |  |  |  |  |  |
| 1.4 | 0.0 | 0.0 |  |  |  |  |  |  |  |  |  |  |  |
| 2.9 | 0.1 | 0.0 |  |  |  |  |  |  |  |  |  |  |  |
| NaN | 0.0 | 0.0 |  |  |  |  |  |  |  |  |  |  |  |
| 4.7 | 0.1 | 0.0 |  |  |  |  |  |  |  |  |  |  |  |
| 0.9 | 0.1 | 0.0 |  |  |  |  |  |  |  |  |  |  |  |
| 1.5 | 0.1 | 0.0 |  |  |  |  |  |  |  |  |  |  |  |
| 0.3 | 0.0 | 0.0 | 646 |  |  |  |  |  |  |  |  |  |  |
| 0.6 | 0.0 | 0.0 |  |  |  |  |  |  |  |  |  |  |  |
| 0.0 | 0.1 | 0.0 |  |  |  |  |  |  |  |  |  |  |  |
| 0.4 | 0.0 | 0.0 |  |  |  |  |  |  |  |  |  |  |  |
| 26.6 | 1.6 | 0.0 |  |  |  |  |  |  |  |  |  |  |  |
| 5.4 | 0.2 | 0.0 |  |  |  |  |  |  |  |  |  |  |  |
| 1.0 | 0.0 | 0.0 |  |  |  |  |  |  |  |  |  |  |  |
| 0.6 | 0.0 | 0.0 |  |  |  |  |  |  |  |  |  |  |  |
| 0.7 | 0.0 | 0.0 |  |  |  |  |  |  |  |  |  |  |  |
| 1.9 | 0.2 | 0.0 |  |  |  |  |  |  |  |  |  |  |  |
| 3.6 | 0.4 | 0.0 | 661 |  |  |  |  |  |  |  |  |  |  |
| 3.7 | 0.4 | 0.0 |  |  |  |  |  |  |  |  |  |  |  |
| 1.4 | 0.1 | 0.0 |  |  |  |  |  |  |  |  |  |  |  |
| NaN | 0.2 | NaN |  |  |  |  |  |  |  |  |  |  |  |
| 29.6 | 2.1 | 0.0 | 666 |  |  |  |  |  |  |  |  |  |  |
| 21.4 | 4.1 | 0.0 |  |  |  |  |  |  |  |  |  |  |  |
| 1.0 | 0.0 | 0.0 |  |  |  |  |  |  |  |  |  |  |  |
| 1.0 | 0.2 | 0.0 |  |  |  |  |  |  |  |  |  |  |  |
| 5.2 | 0.0 | 0.0 |  |  |  |  |  |  |  |  |  |  |  |
| 16.1 | 0.0 | 0.0 |  |  |  |  |  |  |  |  |  |  |  |
| 5.1 | 0.1 | 0.0 | 673 |  |  |  |  |  |  |  |  |  |  |
| 4.1 | 0.1 | 0.0 |  |  |  |  |  |  |  |  |  |  |  |
| 0.4 | 0.0 | 0.0 |  |  |  |  |  |  |  |  |  |  |  |
| 2.1 | 0.0 | 0.0 |  |  |  |  |  |  |  |  |  |  |  |
| 11.7 | 0.1 | 0.0 | 682 |  |  |  |  |  |  |  |  |  |  |
| 18.3 | 0.1 | 0.0 |  |  |  |  |  |  |  |  |  |  |  |
| 9.4 | 0.2 | 0.0 |  |  |  |  |  |  |  |  |  |  |  |
| 12.6 | 0.1 | 0.0 |  |  |  |  |  |  |  |  |  |  |  |
| 3.0 | 0.0 | 0.0 |  |  |  |  |  |  |  |  |  |  |  |
| 0.6 | 0.0 | 0.0 |  |  |  |  |  |  |  |  |  |  |  |
| 6.4 | 0.0 | 0.0 |  |  |  |  |  |  |  |  |  |  |  |
| 7.4 | 0.5 | 0.0 |  |  |  |  |  |  |  |  |  |  |  |
| 7.3 | 2.9 | 0.0 |  |  |  |  |  |  |  |  |  |  |  |
| 6.4 | 1.3 | 0.0 |  |  |  |  |  |  |  |  |  |  |  |
| 4.9 | 0.2 | 0.0 |  |  |  |  |  |  |  |  |  |  |  |
| 1.5 | 0.0 | 0.0 |  |  |  |  |  |  |  |  |  |  |  |
| 0.7 | 0.0 | 0.0 |  |  |  |  |  |  |  |  |  |  |  |
| 0.1 | 0.0 | 0.0 |  |  |  |  |  |  |  |  |  |  |  |
| 6.1 | 0.0 | 0.0 |  |  |  |  |  |  |  |  |  |  |  |
| 4.3 | 0.0 | 0.0 |  |  |  |  |  |  |  |  |  |  |  |
| 3.1 | 0.0 | 0.0 | 700 |  |  |  |  |  |  |  |  |  |  |
| 2.8 | 0.0 | 0.0 |  |  |  |  |  |  |  |  |  |  |  |
| 40.0 | 0.4 | 0.0 | 702 |  |  |  |  |  |  |  |  |  |  |
| 41.0 | 0.4 | 0.0 |  |  |  |  |  |  |  |  |  |  |  |
| 3.3 | 0.0 | 0.0 |  |  |  |  |  |  |  |  |  |  |  |
| 0.9 | 0.0 | 0.0 |  |  |  |  |  |  |  |  |  |  |  |
| 6.1 | 0.3 | 0.0 |  |  |  |  |  |  |  |  |  |  |  |
| 22.6 | 0.0 | 0.0 |  |  |  |  |  |  |  |  |  |  |  |
| 3.0 | 0.6 | 0.0 |  |  |  |  |  |  |  |  |  |  |  |
| 2.8 | 0.2 | 0.0 |  |  |  |  |  |  |  |  |  |  |  |
| 1.5 | 0.1 | 0.0 |  |  |  |  |  |  |  |  |  |  |  |
| 2.8 | 0.0 | 0.0 |  |  |  |  |  |  |  |  |  |  |  |
| 5.2 | 0.1 | 0.0 |  |  |  |  |  |  |  |  |  |  |  |
| 15.7 | 0.2 | 0.0 |  |  |  |  |  |  |  |  |  |  |  |
| 17.5 | 1.4 | 0.0 |  |  |  |  |  |  |  |  |  |  |  |
| 4.4 | 0.6 | 0.0 |  |  |  |  |  |  |  |  |  |  |  |
| 21.7 | 2.4 | 0.0 |  |  |  |  |  |  |  |  |  |  |  |
| 12.8 | 2.6 | 0.0 |  |  |  |  |  |  |  |  |  |  |  |
| 6.1 | 0.4 | 0.0 |  |  |  |  |  |  |  |  |  |  |  |
| 24.7 | 0.3 | 0.0 |  |  |  |  |  |  |  |  |  |  |  |
| 9.5 | 0.0 | 0.0 |  |  |  |  |  |  |  |  |  |  |  |
| 5.0 | 0.0 | 0.0 |  |  |  |  |  |  |  |  |  |  |  |
| 1.6 | 0.1 | 0.0 |  |  |  |  |  |  |  |  |  |  |  |
| 1.3 | 0.1 | 0.0 |  |  |  |  |  |  |  |  |  |  |  |
| 0.0 | 0.8 | 0.0 |  |  |  |  |  |  |  |  |  |  |  |
| 14.3 | 1.2 | 0.0 |  |  |  |  |  |  |  |  |  |  |  |
| 18.0 | 0.5 | 0.0 |  |  |  |  |  |  |  |  |  |  |  |
| 21.5 | 1.3 | 0.0 |  |  |  |  |  |  |  |  |  |  |  |
| 7.1 | 0.1 | 0.0 |  |  |  |  |  |  |  |  |  |  |  |
| 10.7 | 0.2 | 0.0 |  |  |  |  |  |  |  |  |  |  |  |
| 10.4 | 0.5 | 0.0 |  |  |  |  |  |  |  |  |  |  |  |
| 4.2 | 0.2 | 0.0 |  |  |  |  |  |  |  |  |  |  |  |
| 0.7 | 0.0 | 0.0 |  |  |  |  |  |  |  |  |  |  |  |
| 0.8 | 0.0 | 0.0 |  |  |  |  |  |  |  |  |  |  |  |
| 47.1 | 0.1 | 0.0 |  |  |  |  |  |  |  |  |  |  |  |
| 53.1 | 0.8 | 0.0 |  |  |  |  |  |  |  |  |  |  |  |
| 2.6 | 0.3 | 0.0 |  |  |  |  |  |  |  |  |  |  |  |
| 0.7 | 0.0 | 0.0 |  |  |  |  |  |  |  |  |  |  |  |
| 10.6 | 0.5 | 0.0 |  |  |  |  |  |  |  |  |  |  |  |
| 2.7 | 0.3 | 0.0 |  |  |  |  |  |  |  |  |  |  |  |
| 2.2 | 0.9 | 0.0 |  |  |  |  |  |  |  |  |  |  |  |
| 3.1 | 0.4 | 0.0 |  |  |  |  |  |  |  |  |  |  |  |
| 63.1 | 1.3 | 0.1 |  |  |  |  |  |  |  |  |  |  |  |
| 62.2 | 1.1 | 0.1 |  |  |  |  |  |  |  |  |  |  |  |
| 30.5 | 0.8 | 0.0 |  |  |  |  |  |  |  |  |  |  |  |
| 34.5 | 0.0 | 0.0 |  |  |  |  |  |  |  |  |  |  |  |
| 16.1 | 1.3 | 0.0 |  |  |  |  |  |  |  |  |  |  |  |
| 3.4 | 0.4 | 0.0 |  |  |  |  |  |  |  |  |  |  |  |
| 4.0 | 0.1 | 0.0 |  |  |  |  |  |  |  |  |  |  |  |
| 7.1 | 0.2 | 0.0 |  |  |  |  |  |  |  |  |  |  |  |
| 1.1 | 0.0 | 0.0 |  |  |  |  |  |  |  |  |  |  |  |
| 1.7 | 0.0 | 0.0 |  |  |  |  |  |  |  |  |  |  |  |
| 10.2 | 0.8 | 0.0 |  |  |  |  |  |  |  |  |  |  |  |
| 11.2 | 0.5 | 0.0 |  |  |  |  |  |  |  |  |  |  |  |
| 11.2 | 0.1 | 0.0 |  |  |  |  |  |  |  |  |  |  |  |
| 8.1 | 0.1 | 0.0 |  |  |  |  |  |  |  |  |  |  |  |
| 4.6 | 0.0 | 0.0 |  |  |  |  |  |  |  |  |  |  |  |
| 1.4 | 0.0 | 0.0 |  |  |  |  |  |  |  |  |  |  |  |
| 3.2 | 0.1 | 0.0 |  |  |  |  |  |  |  |  |  |  |  |
| 4.1 | 0.0 | 0.0 |  |  |  |  |  |  |  |  |  |  |  |
| 5.6 | 0.4 | 0.0 | 819 |  |  |  |  |  |  |  |  |  |  |
| 5.3 | 0.0 | 0.0 |  |  |  |  |  |  |  |  |  |  |  |
| 8.6 | 0.3 | 0.0 |  |  |  |  |  |  |  |  |  |  |  |
| 17.6 | 0.3 | 0.0 |  |  |  |  |  |  |  |  |  |  |  |
| 13.2 | 0.2 | 0.0 |  |  |  |  |  |  |  |  |  |  |  |
| 8.0 | 0.3 | 0.0 |  |  |  |  |  |  |  |  |  |  |  |
| 5.4 | 0.1 | 0.0 |  |  |  |  |  |  |  |  |  |  |  |
| 11.0 | 0.5 | 0.0 |  |  |  |  |  |  |  |  |  |  |  |
| 56.3 | 0.7 | 0.0 |  |  |  |  |  |  |  |  |  |  |  |
| 63.5 | 0.1 | 0.1 |  |  |  |  |  |  |  |  |  |  |  |
| 0.3 | 0.0 | 0.0 |  |  |  |  |  |  |  |  |  |  |  |
| 0.1 | 0.8 | 0.0 |  |  |  |  |  |  |  |  |  |  |  |
| 0.4 | 0.0 | 0.0 |  |  |  |  |  |  |  |  |  |  |  |
| 1.4 | 0.0 | 0.0 |  |  |  |  |  |  |  |  |  |  |  |
| 11.6 | 0.5 | 0.0 |  |  |  |  |  |  |  |  |  |  |  |
| 13.5 | 1.4 | 0.0 |  |  |  |  |  |  |  |  |  |  |  |
| 16.8 | 0.1 | 0.0 |  |  |  |  |  |  |  |  |  |  |  |
| 13.2 | 0.1 | 0.0 |  |  |  |  |  |  |  |  |  |  |  |
| 3.9 | 0.1 | 0.0 |  |  |  |  |  |  |  |  |  |  |  |
| 4.2 | 0.1 | 0.0 |  |  |  |  |  |  |  |  |  |  |  |
| 0.0 | 0.0 | 0.0 |  |  |  |  |  |  |  |  |  |  |  |
| 0.0 | 0.2 | 0.0 |  |  |  |  |  |  |  |  |  |  |  |
| 0.4 | 0.0 | 0.0 |  |  |  |  |  |  |  |  |  |  |  |
| 1.2 | 0.1 | 0.0 |  |  |  |  |  |  |  |  |  |  |  |
| 18.4 | 0.8 | 0.0 |  |  |  |  |  |  |  |  |  |  |  |
| 13.7 | 0.6 | 0.0 |  |  |  |  |  |  |  |  |  |  |  |
| 18.3 | 0.0 | 0.0 |  |  |  |  |  |  |  |  |  |  |  |
| 43.2 | 1.0 | 0.0 |  |  |  |  |  |  |  |  |  |  |  |
| 12.4 | 0.1 | 0.0 |  |  |  |  |  |  |  |  |  |  |  |
| 8.5 | 0.1 | 0.0 |  |  |  |  |  |  |  |  |  |  |  |
| NaN | 0.2 | 0.0 |  |  |  |  |  |  |  |  |  |  |  |
| 54.5 | 0.0 | 0.0 |  |  |  |  |  |  |  |  |  |  |  |
| 49.0 | 0.4 | 0.0 |  |  |  |  |  |  |  |  |  |  |  |
| 51.6 | 0.4 | 0.0 |  |  |  |  |  |  |  |  |  |  |  |
| 8.2 | 0.5 | 0.0 |  |  |  |  |  |  |  |  |  |  |  |
| 3.3 | 0.8 | 0.0 |  |  |  |  |  |  |  |  |  |  |  |
| 5.9 | 0.3 | 0.0 |  |  |  |  |  |  |  |  |  |  |  |
| 11.2 | 0.7 | 0.0 |  |  |  |  |  |  |  |  |  |  |  |
| 29.7 | 0.0 | 0.0 |  |  |  |  |  |  |  |  |  |  |  |
| 43.6 | 0.1 | 0.0 |  |  |  |  |  |  |  |  |  |  |  |
| 53.3 | 0.5 | 0.0 |  |  |  |  |  |  |  |  |  |  |  |
| NaN | 0.3 | NaN |  |  |  |  |  |  |  |  |  |  |  |
| 37.5 | 2.2 | 0.0 |  |  |  |  |  |  |  |  |  |  |  |
| 44.8 | 1.4 | 0.0 |  |  |  |  |  |  |  |  |  |  |  |
| 0.7 | 0.1 | 0.0 |  |  |  |  |  |  |  |  |  |  |  |
| 1.4 | 0.0 | 0.0 |  |  |  |  |  |  |  |  |  |  |  |
| 32.5 | 1.4 | 0.0 |  |  |  |  |  |  |  |  |  |  |  |
| 53.9 | 1.5 | 0.0 |  |  |  |  |  |  |  |  |  |  |  |
| 6.7 | 0.2 | 0.0 |  |  |  |  |  |  |  |  |  |  |  |
| 9.3 | 0.1 | 0.0 |  |  |  |  |  |  |  |  |  |  |  |
| 4.1 | 0.1 | 0.0 |  |  |  |  |  |  |  |  |  |  |  |
| 3.0 | 0.1 | 0.0 |  |  |  |  |  |  |  |  |  |  |  |
| 0.8 | 0.0 | 0.0 |  |  |  |  |  |  |  |  |  |  |  |
| 1.1 | 0.0 | 0.0 |  |  |  |  |  |  |  |  |  |  |  |
| 4.4 | 0.1 | 0.0 |  |  |  |  |  |  |  |  |  |  |  |
| 5.9 | 0.4 | 0.0 |  |  |  |  |  |  |  |  |  |  |  |
| 35.0 | 2.2 | 0.6 |  |  |  |  |  |  |  |  |  |  |  |
| 87.0 | 0.8 | 0.0 |  |  |  |  |  |  |  |  |  |  |  |
| 8.1 | 0.8 | 0.0 |  |  |  |  |  |  |  |  |  |  |  |
| 44.3 | 1.5 | 0.0 |  |  |  |  |  |  |  |  |  |  |  |

**Supplementary Table 4** HLA class I allotype and allele frequencies of the n=33 meningioma patients included in the study cohort. The HLA-A, -B, and -C allotypes with the top three highest frequencies within the collective are marked in bold

| **HLA allotype** | **Positive patients** | **Allele frequency** | **HLA allotype** | **Positive patients** | **Allele frequency** |
| --- | --- | --- | --- | --- | --- |
| **A*01:01**  **frequency of positive meningioma patients [%]**  0 10 20  | **11%** | 12% | B*39:31 | 2% | 2% |
| **A*02:01** | **20%** | 23% | B*40:01 | 2% | 2% |
| **A*03:01** | **12%** | 12% | B*40:02 | 3% | 3% |
| A*11:01 | 3% | 3% | B*41:01 | 2% | 2% |
| A*23:01 | 3% | 3% | B*44:02 | 2% | 2% |
| **A*24:02** | **11%** | 12% | B*44:03 | 8% | 9% |
| A*26:01 | 2% | 2% | B*49:01 | 2% | 2% |
| A*29:02 | 8% | 8% | B*50:01 | 2% | 2% |
| A*30:01 | 2% | 2% | **B*51:01** | **9%** | 9% |
| A*30:02 | 5% | 5% | B*51:02 | 2% | 2% |
| A*31:01 | 3% | 3% | B*51:08 | 2% | 2% |
| A*32:01 | 5% | 5% | B*55:01 | 3% | 3% |
| A*33:01 | 2% | 2% | B*57:01 | 5% | 5% |
| A*34:01 | 2% | 2% | C*01:02 | 5% | 5% |
| A*66:01 | 2% | 2% | C*02:02 | 8% | 8% |
| A*68:01 | 8% | 8% | C*03:03 | 6% | 6% |
| **B*07:02** | **9%** | 11% | C*03:04 | 3% | 3% |
| B*08:01 | 6% | 6% | **C*04:01** | **14%** | 17% |
| B*13:02 | 5% | 5% | C*05:01 | 2% | 2% |
| B*14:02 | 2% | 2% | **C*06:02** | **11%** | 11% |
| B*15:01 | 5% | 5% | **C*07:01** | **12%** | 14% |
| B*15:35 | 2% | 2% | C*07:02 | 9% | 11% |
| **B*18:01** | **11%** | 11% | C*08:02 | 2% | 2% |
| B*27:05 | 3% | 3% | C*12:03 | 9% | 9% |
| **B*35:01** | **9%** | 11% | C*14:02 | 2% | 2% |
| B*35:03 | 2% | 2% | C*15:02 | 3% | 5% |
| B*37:01 | 2% | 2% | C*16:01 | 6% | 6% |
| B*38:01 | 2% | 2% | C*16:02 | 2% | 2% |
| B*39:01 | 2% | 2% | C*17:01 | 2% | 2% |

**Supplementary Table 5** Meningioma-associated HLA class I- and II-presented antigens identified on at least three tumors. GTEx profiles were assessed from all available datasets excepting EBV-transformed lymphocytes and cultured fibroblasts. Color codes were defined as follows: ■ < 10 TPM in any tissue, ♦ > 10 TPM in testes and < 10 TPM in other tissues (CTA-like expression profile), ■ 10-20 TPM in any tissue, ■ 20-30 TPM in any tissue, ■ > 30 TPM in any tissue. HLA restrictions not passing manual assessment as quality control are indicated in italic and were excluded from downstream analyses as peptides matching per patient worldwide. HLA class II-presented proteins neither identified with peptides exceeding a length of twelve AA nor with different sequences across patients were not considered for this listing of candidate target antigens. Eight of the listed HLA class II-presented antigens (DRGX, KIR2DS4, SGK3, PCED1B, STAT5B, ESCO1, ANGEL2, RFWD3) were detected with only one peptide in each case

| **Antigen** | **Frequency of positive patients** | **Peptide sequence** | **HLA restriction** | **UniProt accession**  **GTEx profile** | **Positive non-menin-geal tumors** |
| --- | --- | --- | --- | --- | --- |
| **Meningioma-associated HLA class I antigens** | | | | | |
| Nicotinamide mononucleotide adenylyltransferase 2 (NMNA2) | 30% / WHO I-III  MNG1  MNG3  MNG6  MNG499  MNG501  MNG636  MNG642  MNG661  MNG734  MNG814 | EEIELRILL  ENANLGTVMR  ENANLGTVMR  NANLGTVMR  ENANLGTVMR  IVSPVHDSY  EEIELRILL  SVLEHHRDLMK  IVSPVHDSY  SVVSSTKSR  ENANLGTVMR | B*18:01  A*68:01  A*68:01  A*68:01  A*68:01  B*15:01  B*44:03  A*11:01  A*30:02  A*66:01  A*68:01 | Q9BZQ4 ■ | 8 |
| Protein Wnt-5a (WNT5A) | 18% / WHO I+II  MNG6  MNG628  MNG637  MNG638  MNG641  MNG702 | AMSSKFFLV  AMSSKFFLV  NPVQMSEVY  AMSSKFFLV  AMSSKFFLV  AMSSKFFLV  AMSSKFFLV | A*02:01  A*02:01  B*35:01  A*02:01  A*02:01  A*02:01  A*02:01 | P41221 ■ | 43 |
| T-box transcription factor TBX15/18 (TBX15/18)  2 peptides multi-map to non-MNG-exclusive TBX20 (Q9UMR3) or to MNG-exclusive TBX22 (Q9Y458) | 18% / WHO I-III  MNG7  MNG635  MNG636  MNG646  MNG682  MNG734 | GLDPHQQYY (TBX15/18)  SQMSVHMV (TBX15)  FHDIGTEMI (TBX15/22)  THQGSYNTF (TBX18)  DIVPVDNKRYR (TBX15/18/20)  THQGSYNTF (TBX18)  GLDPHQQYY(TBX15/18)  KTFNFPETVF (TBX15)  GLDPHQQYYI (TBX15/18)  YQNQQITRL (TBX15/18) | A*01:01;  A*30:02  B*13:02  B*38:01  B*38:01  A*33:01  B*38:01  A*01:01  B*15:01  A*02:01  C*02:02 | Q96SF7 ■  O95935 ■ | 15  9 |
| Protein odd-skipped-related 1 (OSR1) | 15% / WHO I+II  MNG7  MNG501  MNG628  MNG641  MNG814 | APVPIHPSL  FPWFPHVI  SLVDARFQL  SLVDARFQL  APVPIHPSL | B*07:02  B*51:01  A*02:01  A*02:01;  C*17:01  B*07:02 | Q8TAX0 ■ | 6 |
| Protein SSX5/9 (SSX5/9)  peptides multi-map to MNG-exclusive obsolete UniProt IDs of SSX10 (A6NEJ1) and SSX11 (A6NNU9); not in GTEx | 12% / WHO I+II  MNG1  MNG623  MNG673  MNG679 | SEKIIYVY  SEKIIYVY  SEKILYVY  SEKIIYVY | B*18:01  B*18:01  B*44:03  B*44:03 | O60225 ■ Q7RTT3 ■ | 13  12 |
| Frizzled-7 (FZD7)  3 peptides multi-map to non-MNG-exclusive FZD1 (Q9UP38) or FZD2 (Q14332) | 12% / WHO I+II  MNG635  MNG661  MNG673  MNG814 | HQFYPLVKV (FZD1/2/7)  TYLVDMRRF (FZD1/7)  FSDDGYRTV (FZD7)  VPAVKTITI (FZD2/7) | B*13:02  A*24:02  C*03:04;  C*16:01  B*07:02 | O75084 ■ | 7 |
| E3 ubiquitin-protein ligase ZNRF2 (ZNRF2) | 12% / WHO I+II  MNG1  MNG6  MNG628  MNG638 | DEMDLHLVM  DEMDLHLVM  DEMDLHLVM  DEMDLHLVM | B*18:01  B*18:01  B*18:01  B*18:01 | Q8NHG8 ■ | 8 |
| Tctex1 domain-containing protein 2 (TC1D2) | 12% / WHO I  MNG7  MNG632  MNG661  MNG702 | KLKEMGFDRY  KLKEMGFDRY  KLKEMGFDRY  LSENIKDKL | A*30:02  A*30:02  A*30:02  *C*06:02* | Q8WW35 ■ | 5 |
| Insulin-like growth factor-binding protein 6 (IGFBP6) | 12% / WHO I+II  MNG1  MNG628  MNG661  MNG666 | DEAPLRAL  DEAPLRAL  TPHRLLPPL  DEAPLRAL  TPHRLLPPL | B*18:01  B*18:01  B*35:01  B*18:01  B*07:02 | P24592 ■ | 2 |
| Uncharacterized protein C8orf34 (C8orf34) | 12% / WHO I+II  MNG499  MNG635  MNG642  MNG833 | YPAEPQAKV  YPAEPQAKV  YPAEPQAKV  YPAEPQAKV | B*51:01;  B*51:02  B*55:02 B*51:01  B*51:01 | Q49A92 ■ | 3 |
| Globoside alpha-1,3-N-acetylgalactosaminyltrans-ferase 1 (GBGT1) | 12% / WHO I+II  MNG6  MNG635  MNG814  MNG833 | ESAEEFFMR  AVFGGQVAR  ESAEEFFMR  RPTQLLTL  KYTHFIQSF | A*68:01  A*31:01  A*68:01  B*07:02  A*24:02 | Q8N5D6 ■ | 26 |
| Xyloside xylosyltransferase 1 (XXLT1) | 12% / WHO I-III  MNG3  MNG642  MNG734  MNG833 | ETFSSATKR  ETFSSATKR  ETFSSATKRL  YYSDSIFFL | A*68:01  A*34:01  A*66:01  A*24:02 | Q8NBI6 ■ | 13 |
| Solute carrier family 25 member 44 (SLC25A44) | 12% / WHO I-III  MNG7  MNG641  MNG702  MNG734 | ILQADGLRGFY  SLVAQSITV  SLVAQSITV  SLVAQSITV | A*01:01  A*02:01  A*02:01  A*02:01 | Q96H78 ■ | 10 |
| Forkhead box protein (FOX) E3, D4-like 1/2/3/5/6  peptides multi-map to FOXB2 (Q5VYV0) and to non-MNG-exclusive FOXD1 (Q16676), FOXD2 (O60548), FOXD3 (Q9UJU5), FOXD4 (Q12950), FOXB1 (Q99853), FOXC1 (Q12948), FOXC2 (Q99958), FOXE1 (O00358), FOXG1 (P55316), FOXI1 (Q12951), FOXI2 (Q6ZQN5), FOXI3 (A8MTJ6), FOXJ1 (Q92949), FOXK1 (P85037), FOXK2 (Q01167), FOXL1 (Q12952), FOXL2 (P58012), FOXS1 (O43638) | 12% / WHO I+II  MNG6  MNG7  MNG646  MNG814 | DMFDNGSFLR  QNSLRHNL  QNSLRHNL  DMFDNGSFLR | A*68:01  B*08:01  B*08:01  A*68:01 | Q13461 ■  Q9NU39 ■  Q6VB85 ■  Q6VB84 ■  Q5VV16 ■  Q3SYB3 ■ | 6  6  5  4  5  4 |
| Sterol O-acyltransferase 2 (SOAT2) 2 peptides multi-map to non-MNG-exclusive SOAT1 (P35610) | 9% / WHO I+II  MNG501  MNG624  MNG833 | YPVMLILFL  NAFAEMLRF  NAFAEMLRF | B*51:01  B*35:01  B*35:01 | O75908 ■ | 7 |
| Cadherin-3 (CDH3) | 9% / WHO I+II  MNG641  MNG666  MNG814 | LPRGPLASLL  LPRGPLASLL  LPRGPLASLL | B*07:02  B*07:02  B*07:02 | P22223 ■ | 17 |
| Folate receptor gamma (FOLR3)  peptides multi-map to non-MNG-exclusive FOLR2 | 9% / WHO I+II  MNG635  MNG646  MNG661 | SYFPTPAAL  FESYFPTPAA  SYFPTPAAL | C*06:02  B*40:02  A*24:02 | P41439 ■ | 9 |
| Transmembrane protein 87A (TMEM87A) | 9% / WHO II+III  MNG642  MNG635  MNG734 | DAPYIFIV  HPSPLSFFSA  AAWLQVLPV | B*51:01  B*55:01  C*12:03 | Q8NBN3 ■ | 41 |
| Transmembrane protein 255B (TMEM255B) | 9% / WHO I+II  MNG632  MNG819  MNG833 | NPAQQILAY  YYPGIILGF  YYPGIILGF | B*35:01  A*23:01  A*24:02 | Q8WV15 ■ | 16 |
| Ninjurin-2 (NINJ2) | 9% / WHO I+III  MNG7  MNG632  MNG734 | EQGPSSHYY  EQGPSSHYY  NEVEKQWRL | A*30:02  A*30:02  B*40:02 | Q9NZG7 ■ | 13 |
| Rho-related GTP-binding protein RhoD (RHOD) | 9% / WHO I-III  MNG3  MNG642  MNG734 | EVALSSRGR  EVALSSRGR  EVALSSRGR | A*68:01  A*34:01  A*66:01 | O00212 ■ | 7 |
| Beta-1,4-galactosyltransferase 4 (B4GALT4) | 9% / WHO I  MNG673  MNG679  MNG682 | FNLTFHLSY  FNLTFHLSY  FNLTFHLSY | A*29:02  A*29:02  A*29:02 | O60513 ■ | 6 |
| cAMP-responsive element-binding protein-like 2 (CREBL2) | 9% / WHO I-III  MNG6  MNG638  MNG734 | IPSEIKALL  LRYQYLEEL  LRYQYLEEL | B*35:03  C*07:01  B*39:31 | O60519 ■ | 19 |
| Mitochondrial tRNA-specific 2-thiouridylase 1 (MTU1) | 9% / WHO I  MNG3  MNG673  MNG679 | EVFEQKHVKK  NFEHFLLQY  NFEHFLLQY | A*68:01  A*29:02  A*29:02 | O75648 ■ | 10 |
| NKG2-C type II integral membrane protein (NKG2C) / NKG2-E type II integral membrane protein (NKG2E) | 9% / WHO I+II  MNG499  MNG501  MNG642 | VTINGLAFK  LPSSWIGVF  VTINGLAFK | A*11:01  B*51:01  A*11:01 | P26717 ■  Q07444 ■ | 1  1 |
| Protein AF-9 (AF-9) | 9% / WHO I+II  MNG6  MNG628  MNG661 | SEALFKSF  SEALFKSF  SEALFKSF | B*18:01  B*18:01  B*18:01 | P42568 ■ | 3 |
| 1-phosphatidylinositol 4,5-bisphosphate phosphodiesterase delta-4 (PLCD4) | 9% / WHO I-III  MNG1  MNG673  MNG734 | EELPLEQGF  ALSSLVIYL  ILFKDVVATV | B*18:01  A*02:01  A*02:01 | Q9BRC7 ■ | 2 |
| Paraneoplastic antigen Ma2 (PNMA2) | 9% / WHO I+II  MNG1  MNG3  MNG673 | AYVLRLETL  SEVQGKGGVW  AEIQEVLQETL  SEVQGKGGVW | A*24:02  B*44:03  B*40:01  B*44:03 | Q9UL42 ■ | 32 |

| **Meningioma-associated HLA class II antigens** | | | | |
| --- | --- | --- | --- | --- |
| Inactive serine protease 35 (PRSS35) | 30% / WHO I+II MNG6 MNG7 MNG501 MNG634    MNG638       MNG641   MNG673        MNG702 MNG814  MNG833 | SRFSILDKRFLTNFPFS LPTPSLSELEDY LPTPSLSELEDYL LPTPSLSELEDYL LPTPSLSELEDYLSYE SRFSILDKRFLTNFPFS SRFSILDKRFLTNFPFS KVPRIVSERTFHLTSP KVPRIVSERTFHLTSPA LPTPSLSELED LPTPSLSELEDY LPTPSLSELEDYL LPTPSLSELEDYLSY LPTPSLSELEDYLSYE GTDSRFSILDKRFLTNFPFS SRFSILDKRFLTNFPFS SRFSILDKRFLTNFPFST DSRFSILDKRFLTNFPFS DSRFSILDKRFLTNFPFST GTDSRFSILDKRFLTNFPF GTDSRFSILDKRFLTNFPFS GTDSRFSILDKRFLTNFPFST SRFSILDKRFLTNFPF SRFSILDKRFLTNFPFS SRFSILDKRFLTNFPFST SRFSILDKRFLTNFPFS RFSILDKRFLTNFPFS SRFSILDKRFLTNFPFS DSRFSILDKRFLTNFPFS GTDSRFSILDKRFLTNFPFS RFSILDKRFLTNFPFS SRFSILDKRFLTNFPF SRFSILDKRFLTNFPFS SRFSILDKRFLTNFPFST | Q8N3Z0 ■ | 3 |
| Lactosylceramide 4-alpha-galactosyltransferase (A4GALT) | 27% / WHO I-III MNG7 MNG623 MNG661 MNG666   MNG673  MNG682 MNG734 MNG819 MNG833 | KPPDLLLRLLRGAP TQSRYVLNGAFLAFERR QSRYVLNGAFLAFER QSRYVLNGAFLAFER TQSRYVLNGAFLAFER TQSRYVLNGAFLAFERR GTQSRYVLNGAFLAFERRH TQSRYVLNGAFLAFER TQSRYVLNGAFLAFER QSRYVLNGAFLAFERR TQSRYVLNGAFLAFER QSRYVLNGAFLAFER QSRYVLNGAFLAFERR TQSRYVLNGAFLAFER TQSRYVLNGAFLAFERR TQSRYVLNGAFLAFERRH | Q9NPC4 ■ | 0 |
| Fibrillin-2 (FBN2) | 21% / WHO I-III MNG1     MNG3 MNG612 MNG641 MNG700 MNG702     MNG734 | DDSVFRIHQRNGLSY DDSVFRIHQRNGLSYL GNDDSVFRIHQRNGLSYLH IQPLNNHIRYVISQG NDDSVFRIHQRNGLSY DDSVFRIHQRNGLSY LRPAIQPLNNHIRYV LRPAIQPLNNHIRYV KKDSRQKRSI DSVFRIHQRNGLSY GNDDSVFRIHQRNGLSY GNDDSVFRIHQRNGLSYLH IQPLNNHIRYVISQG NDDSVFRIHQRNGLSY LRPAIQPLNNHIRYV RPAIQPLNNHIRYV | P35556 ■ | 7 |
| Sushi, nidogen and EGF-like domain-containing protein 1 (SNED1) | 21% / WHO I-III MNG7 MNG638 MNG642   MNG666 MNG679 MNG702 MNG734 | QTVLITDGKLSFTIFN DRFTFRALLPGKRY DRFTFRALLPGKRYT EPAHLYIITSPRDG EPAHLYIITSPRDGAD TKSRYVPNGKLASYT TRLFSETKAFPVWE TKSRYVPNGKLASYT ASTISVQWALHRIR | Q8TER0 ■ | 1 |
| MAGUK p55 subfamily member 6 (MPP6) | 18% / WHO I-III MNG1 MNG2  MNG6 MNG623 MNG641     MNG734 | INNQLLPVDAIRILG INNQLLPVDAIRILG INNQLLPVDAIRILGIH DRHEIQIYEEVAKMPP INNQLLPVDAIRILG DRHEIQIYEEVAKMPP DRHEIQIYEEVAKMPPF DRHEIQIYEEVAKMPPFQ FDRHEIQIYEEVAKMPP FDRHEIQIYEEVAKMPPFQ DRHEIQIYEEVAKMPP DRHEIQIYEEVAKMPPF | Q9NZW5 ■ | 5 |
| Dorsal root ganglia homeobox protein (DRGX) | 15% / WHO I+II MNG3 MNG501 MNG666 MNG702 MNG833 | LAMKINLTEARVQVWF LAMKINLTEARVQVWF LAMKINLTEARVQVWF LAMKINLTEARVQVWF LAMKINLTEARVQVWF | A6NNA5 ■ | 6 |
| Melanoma-associated antigen 10 (MAGEA10) 1 peptide multi-maps to non-MNG-exclusive MAGEA9 (P43362) | 15% / WHO I+II MNG499 MNG624 MNG641 MNG682 MNG700 | KLLTQDWVQENYLEYRQVPGSDP KLLTQDWVQENYLEYRQVPGSDP KLLTQDWVQENYLEYRQVPGSDP ESLPRSEIDEKVTDLVQFLLFKYQM KLLTQDWVQENYLEYRQVPGSDP | P43363 ■ | 3 |
| Nuclear factor of activated T‑cells, cytoplasmic 2 (NFATC2) | 15% / WHO I+II MNG1  MNG6 MNG7 MNG624  MNG702 | DQTYLDDVNEIIR LDQTYLDDVNEIIR KDKSQPNMLFVEIPEYR DQTYLDDVNEIIR DQTYLDDVNEIIR LDQTYLDDVNEIIR DQTYLDDVNEIIR KPHAFYQVHRITGKT LDQTYLDDVNEIIR | Q13469 ■ | 20 |
| Uncharacterized protein KIAA1586 (KIAA1586) | 15% / WHO I+II MNG5 MNG501 MNG637 MNG642 MNG702 | EVDLNDFREFVNNNIK ELETEIIKIGRVMGPRW ELETEIIKIGRVMGPRW ELETEIIKIGRVMGPRW ELETEIIKIGRVMGPRW | Q9HCI6 ■ | 5 |
| ZAR1-like protein (ZAR1L) | 12% / WHO I+II MNG501 MNG638 MNG702 MNG833 | MGPPTFLARPGLLVPANAP SPRLCKPNTKEVGVQVSPRVDKAV MGPPTFLARPGLLVPANAP MGPPTFLARPGLLVPANAP | A6NP61 ■ | 1 |
| G protein-regulated inducer of neurite outgrowth 2 (GPRIN2) | 12% / WHO I+II MNG638 MNG679 MNG682 MNG702 | IQKHLEMQFEQLQRAPASEDS IQKHLEMQFEQLQRAPASEDSL IQKHLEMQFEQLQRAPASEDSL IQKHLEMQFEQLQRAPASEDSL | O60269 ■ | 2 |
| Bone morphogenetic protein 5 (BMP5) 1 peptide multi-maps to non-MNG-exclusive BMP6 (P18075) | 12% / WHO I+II MNG638 MNG642     MNG679  MNG702 | SNVILKKYRNMVVRS AAEFRIYKDRSNN AAEFRIYKDRSNNR AEFRIYKDRSNNR TAAEFRIYKDRSNN TAAEFRIYKDRSNNR RDADLFLLDTRK YTNRDADLFLLDTRK AFFKATEVHFR SSNVILKKYRNMVVR | P22003 ■ | 0 |
| Transcription factor SOX-6 (SOX6) | 12% / WHO I+II MNG4 MNG499 MNG642 MNG679 | ASMQVSPGAKMPS KTDGGSLAGNEMINGEDEMEMYDDY EMEMYDDYEDDP ITQLISLREQLLAAHDEQKKLAAS | P35712 ■ | 5 |
| Zinc finger E-box-binding homeobox 2 (ZEB2) | 12% / WHO I+II MNG7 MNG623 MNG624 MNG635 | SVNGRMRNNIKTGSSP EEYFAKRKLEERDGHAVSIEEYLQ EEYFAKRKLEERDGHAVSIEEYLQ EEYFAKRKLEERDGHAVSIEEYLQ | O60315 ■ | 1 |
| Mucin-4 (MUC4) | 12% / WHO I+II MNG499  MNG501 MNG628 MNG638 | GRRDLRFQPVSIGR GRRDLRFQPVSIGRWG DNGQIIFPESDYQIFSYPNPLPT SNILHASASLPP APIPILPERGVSLFPY APIPILPERGVSLFPYG | Q99102 ■ | 12 |
| Kinesin light chain 2 (KLC2) peptides multi-map to non-MNG-exclusive KLC1 (Q07866) | 12% / WHO I+III MNG6  MNG612 MNG636 MNG734 | HNLVIQYASQGRYE LHNLVIQYASQGRYE LHNLVIQYASQGRYE LHNLVIQYASQGRYE HNLVIQYASQGRYE | Q9H0B6 ■ | 6 |
| Calcium-binding protein 39‑like (CAB39L) | 12% / WHO I+II MNG638 MNG641 MNG666 MNG702 | GKKDVTQIFNNILRRQ DRHNFAIMTKYISKPE DRHNFAIMTKYISKPE DRHNFAIMTKYISKPE | Q9H9S4 ■ | 2 |
| Calpain-14 (CAPN14) | 9% / WHO I MNG7 MNG499 MNG673 | MLRVENMEDVFQN IMLSDDVCQLMLIRYGGPR LPPEFFQRNTPLSQPDRFLKEKE | A8MX76 ■ | 1 |
| Methylenetetrahydrofolate reductase (MTHFR) | 9% / WHO I+II MNG499 MNG638 MNG814 | IKDVIEPIKDNDAAIR KGENITNAPELQPNAVT IKDVIEPIKDNDAAIR | P42898 ■ | 9 |
| Killer cell immunoglobulin-like receptor 2DS4 (KIR2DS4) | 9% / WHO I MNG7 MNG623 MNG624 | MSLMVIIMACVGFFLLQGAW MSLMVIIMACVGFFLLQGAW MSLMVIIMACVGFFLLQGAW | P43632 ■ | 1 |
| Probable allantoicase (ALLC) | 9% / WHO I+II MNG6 MNG642 MNG673 | PSSICLLRPREKPM DFFAPAENLIKSDSPCF IPERGTRTGAAATPEEFEAIAELKS | Q8N6M5 ■ | 1 |
| Tubulin polyglutamylase TTLL6 (TTLL6) | 9% / WHO I+II MNG632 MNG702 MNG833 | QMKKKVEMQGE QMKKKVEMQGE SEEKGDSSKEDPKETVALAFV | Q8N841 ♦ | 2 |
| Serine/threonine-protein kinase Sgk3 (SGK3) | 9% / WHO I+II MNG682 MNG702 MNG833 | TFCGTPEYLAPEVIRKQPYDNT TFCGTPEYLAPEVIRKQPYDNT TFCGTPEYLAPEVIRKQPYDNT | Q96BR1 ■ | 4 |
| PC-esterase domain-containing protein 1B (PCED1B) | 9% / WHO I-III MNG634 MNG641 MNG734 | NFMVGPQLPMPFFPTPRYQ NFMVGPQLPMPFFPTPRYQ NFMVGPQLPMPFFPTPRYQ | Q96HM7 ■ | 4 |
| Mitochondrial import inner membrane translocase subunit TIM44 (TIMM44) | 9% / WHO I MNG4 MNG6 MNG682 | KRTEFAGDKFKEEKVFE KWYQQWKDFKENNVVFNRFFEMKMK KWYQQWKDFKENNVVFNRFFEMKMK | O43615 ■ | 1 |
| Ubiquitin conjugation factor E4 B (UBE4B) | 9% / WHO I+II MNG641 MNG666 MNG702 | KDLIGQILMEVLMMSTQTRDEN VDMFHILTKQVQKPF VDMFHILTKQVQKPF | O95155 ■ | 5 |
| Protein SGT1 (ECD) | 9% / WHO I MNG5 MNG6 MNG632 | DSDDLDDEDFECLDSDDDLDF LRDPIDLRACRVFKTFLPETRIMTS EKIQASLHRAHCFL | O95905 ■ | 4 |
| T-lymphocyte activation antigen CD86 (CD86) | 9% / WHO I MNG7  MNG623 MNG624 | DKTRLLSSPFSIELEDPQPPP TDKTRLLSSPFSIELEDPQPPP DKTRLLSSPFSIELEDPQPPP DKTRLLSSPFSIELEDPQPPP | P42081 ■ | 22 |
| Signal transducer and activator of transcription 5B (STAT5B) | 9% / WHO I+II MNG3 MNG702 MNG833 | DGVMEVLKKHLKPH DGVMEVLKKHLKPH DGVMEVLKKHLKPH | P51692 ■ | 13 |
| Nucleoporin GLE1 (GLE1) | 9% / WHO I+II MNG628 MNG634 MNG814 | ISGIIRASSESSYPTAE KEEGQIRLRALYALQEEML KQAEQERLRKEEGQI | Q53GS7 ■ | 3 |
| N-acetyltransferase ESCO1 (ESCO1) | 9% / WHO I+II MNG499 MNG635 MNG641 | KVLEVKSDSKEDENLVINEVINSPK KVLEVKSDSKEDENLVINEVINSPK KVLEVKSDSKEDENLVINEVINSPK | Q5FWF5 ■ | 14 |
| Protein angel homolog 2 (ANGEL2) | 9% / WHO I-III MNG634 MNG734 MNG833 | DHYGAEIRPSLESLGY DHYGAEIRPSLESLGY DHYGAEIRPSLESLGY | Q5VTE6 ■ | 0 |
| Meteorin-like protein (METRNL) | 9% / WHO I+II MNG666  MNG702  MNG833 | GFQYELVRRHRASD TGFQYELVRRHRASD GFQYELVRRHRASD TGFQYELVRRHRASDLH GFQYELVRRHRASD | Q641Q3 ■ | 3 |
| E3 ubiquitin-protein ligase RFWD3 (RFWD3) | 9% / WHO I MNG3 MNG666 MNG702 | TNFISGLQRLHGMLEFL TNFISGLQRLHGMLEFL TNFISGLQRLHGMLEFL | Q6PCD5 ■ | 2 |
| Highly divergent homeobox (HDX) | 9% / WHO I+II MNG634 MNG635 MNG666 | QRSYKPEHTGPALHNLC FIENELEIQKQKYFKLQ SEMTVPQKPSVCHRPCKIEP | Q7Z353 ■ | 2 |
| Signal-regulatory protein delta (SIRPD) | 9% / WHO I+II MNG1  MNG3 MNG673 | PLPSLLLYLLLELA PLPSLLLYLLLELAG PLPSLLLYLLLELA PLPSLLLYLLLELA | Q9H106 ♦ | 3 |
| Uncharacterized protein C1orf112 (C1orf112) | 9% / WHO I+II MNG3 MNG6 MNG642 | FVSSLGKLF FVSSLGKLF KAVFYSFEQCSGELSLP | Q9NSG2 ♦ | 1 |

**Supplementary Table 6** Meningioma-associated HLA class I ligands presented on at least five tumors. Peptide sequence, HLA restriction, frequency of positive patients, UniProt correspondence, protein frequency on meningiomas, on non-meningeal tumors and on dura/benign samples. Peptides already reported to derive from meningioma-associated antigens were excluded from this listing. The number of positive non-meningeal tumors was based on n=841 HLA class I peptidome datasets. HLA restrictions not passing manual assessment as quality control are indicated in italic and were excluded from downstream analyses such as calculation of peptides matching per patient worldwide

| **Peptide sequence** | **HLA restriction** | **Frequency of positive patients** | | **Antigen (UniProt accession)** | **Protein frequency on meningiomas** | **Peptide-positive non-meningeal tumors**  **Protein frequency on non-meningeal tumors** | **Protein frequency on dura (n=9) / benign samples (n=418)** |
| --- | --- | --- | --- | --- | --- | --- | --- |
| TLQSTLLLL | A*02:01 C*17:01 | 30% MNG501 MNG628 MNG641 MNG673 MNG702 | WHO I-III MNG612 MNG638 MNG666 MNG700 MNG734 | Mimecan (OGN; P20774) | 61% | 1 2% | 11% 5% |
| QTFPNVREM | B*57:01 C*03:04 C*06:02 C*12:03 | 30% MNG2 MNG5 MNG634 MNG636 MNG666 | WHO I-III MNG3 MNG628 MNG635 MNG661 MNG734 | Forkhead box protein C2 (FOXC2; Q99958) | 85% | 0 7% | 67% 8% |
| IPISNILMV | B*07:02 B*51:01 B*51:02 B*55:01 | 24% MNG499 MNG612MNG642 MNG814 | WHO I-III MNG501 MNG635 MNG702 MNG833 | Interferon-induced protein 44-like (IFI44L; Q53G44) | 30% | 37 12% | 11% 3% |
| LLLPVVSFA | A*02:01 | 21% MNG612 MNG641 MNG673 MNG734 | WHO I-III MNG628 MNG666 MNG702 | Cathepsin K (CTSK; P43235) | 21% | 29 7% | 0% 4% |
| SLPELVHAV | A*02:01 | 21% MNG6 MNG641 MNG673 MNG734 | WHO I-III MNG628 MNG666 MNG702 | Sestrin-3 (SESN3; P58005) | 11% | 23 11% | 33% 4% |
| YSLEKVFGI | A*02:01 C*02:02 *C*06:02* *C*07:01* C*12:03 C*17:01 | 21% MNG2 MNG612 MNG641 MNG734 | WHO I-III MNG5 MNG638 MNG666 | Melanoma-associated antigen D2 (MAGED2; Q9UNF1) | 94% | 24 52% | 56% 48% |
| EVYGTGVASTR | A*34:01 A*66:02 A*68:01 | 21% MNG3 MNG499 MNG642 MNG814 | WHO I-III MNG6 MNG632 MNG734 | Serine palmitoyl-transferase 3 (SPTLC3; Q9NUV7) | 76% | 2 20% | 33% 17% |
| KAIDYIRFL | C*03:04 C*12:03 C*16:01 | 21% MNG1 MNG628 MNG666 MNG734 | WHO I-III MNG4 MNG636 MNG673 | Sterol regulatory element-binding protein 1 (SREBF1; P36956) | 55% | 12 23% | 11% 13% |
| KLNNLTFLY | A*03:01 A*29:02 A*30:02 B*15:01 | 21% MNG1 MNG641 MNG673 MNG814 | WHO I+II MNG632 MNG646 MNG682 | Mimecan (OGN; P20774) | 61% | 2 2% | 11% 5% |
| SSFPTVVIY | C*02:02 C*12:03 | 21% MNG628 MNG637 MNG666 MNG734 | WHO I-III MNG636 MNG661 MNG702 | GPN-loop GTPase 1 (GPN1; Q9HCN4) | 49% | 32 25% | 22% 15% |
| YPSGIHLEL | B*07:02 B*35:01 | 21% MNG2 MNG632 MNG642 MNG814 | WHO I+II MNG7 MNG634 MNG666 | Sulfate transporter (SLC26A2; P50443) | 70% | 2 4% | 33% 3% |
| LPYNTSLVEM | B*07:02 B*35:01 | 18% MNG7 MNG632 MNG641 | WHO I+II MNG628 MNG634 MNG814 | Tropomodulin-1 (TMOD1; P28289) | 67% | 2 8% | 22% 11% |
| NYAGALMYF | A*24:02 | 18% MNG1 MNG635 MNG661 | WHO I+II MNG628 MNG638 MNG833 | Sodium leak channel non-selective protein (NALCN; Q8IZF0) | 24% | 2 2% | 0% 1% |
| SELLVVKM | B*18:01 B*40:02 | 18% MNG1 MNG628 MNG646 | WHO I+II MNG6 MNG638 MNG661 | Obscurin-like protein 1 (OBSL1; O75147) | 45% | 5 17% | 33% 15% |
| VYGLYTSFF | A*24:02 | 18% MNG5 MNG635 MNG661 | WHO I+II MNG628 MNG638 MNG833 | Sulfate transporter (SLC26A2; P50443) | 70% | 3 4% | 33% 3% |
| DGYIEVIGF | B*35:01 *B*51:01* | 18% MNG624 MNG632 MNG682 | WHO I+II MNG628 MNG634 MNG833 | Diacylglycerol kinase iota / zeta (DGKI / DGKZ; O75912 / Q13574) | 18% / 39% | 34 6% / 19% | 0% / 0% 3% / 16% |
| DVIDGPISQR | A*66:01 A*68:01 | 18% MNG3 MNG499 MNG734 | WHO I-III MNG6 MNG632 MNG814 | Signal-induced proliferation-associated 1-like protein 1 (SIPA1L1; O43166) | 49% | 7 20% | 33% 18% |
| EVQDRVMLTGR | A*33:01 A*68:01 | 18% MNG3 MNG499 MNG634 | WHO I MNG6 MNG632 MNG636 | Protein yippee-like 5 (YPEL5; P62699) | 70% | 28 47% | 56% 41% |
| SMVEDITGLRL | A*02:01 | 18% MNG501 MNG638 MNG666 | WHO I-III MNG612 MNG641 MNG673 | Desmoplakin (DSP; P15924) | 97% | 26 34% | 56% 32% |
| SVFAGVVGV | A*02:01 | 18% MNG6 MNG641 MNG673 | WHO I-III MNG612 MNG666 MNG702 | Guanylate cyclase soluble subunit alpha-3 (GUCY1A3; Q02108) | 64% | 42 17% | 56% 16% |
| VPYSRALIM | B*35:03 B*51:01 B*51:02 | 18% MNG6 MNG612 MNG702 | WHO I-III MNG499 MNG642 MNG833 | Lysoplasmaloge-nase-like protein TMEM86A (TMEM86A; Q8N2M4) | 24% | 27 5% | 11% 2% |
| YENLLKASF | B*18:01 B*15:35 | 18% MNG1 MNG628 MNG642 | WHO I+II MNG6 MNG638 MNG661 | Desmoplakin (DSP; P15924) | 97% | 12 34% | 56% 32% |
| AANGVFHVV | C*12:03 | 15% MNG628 MNG661 MNG734 | WHO I-III MNG636 MNG666 | Stabilin-1 (STAB1; Q9NY15) | 79% | 19 34% | 56% 31% |
| AEFPELAAF | B*18:01 B*40:02 B*44:03 | 15% MNG1 MNG636 MNG646 | WHO I+II MNG6 MNG638 | Uncharacterized protein KIAA1755 (KIAA1755; Q5JYT7) | 45% | 0 2% | 0% 2% |
| DEDRFMMQF | B*18:01 | 15% MNG1 MNG628 MNG661 | WHO I+II MNG6 MNG638 | Formin-like protein 2 (FMNL2; Q96PY5) | 48% | 3 18% | 11% 14% |
| DEKFHIAY | B*18:01 | 15% MNG1 MNG623 MNG661 | WHO I+II MNG6 MNG628 | Sestrin-1 (SESN1; Q9Y6P5) | 67% | 6 28% | 56% 34% |
| DMFATPQYR | A*33:01 A*68:01 | 15% MNG3 MNG499 MNG814 | WHO I MNG6 MNG636 | Adiponectin receptor protein 2 (ADIPOR2; Q86V24) | 24% | 18 8% | 0% 3% |
| DVFQHSQSR | A*33:01 A*34:01 A*66:01 A*68:01 | 15% MNG3 MNG636 MNG734 | WHO I-III MNG632 MNG642 | Constitutive coactivator of PPAR-gamma-like protein 1 (FAM120A; Q9NZB2) | 85% | 3 42% | 33% 36% |
| DYTIGFGKF | A*24:02 | 15% MNG1 MNG628 MNG833 | WHO I+II MNG5 MNG661 | Integrin beta-4 (ITGB4; P16144) | 76% | 7 21% | 22% 27% |
| EIVGNLPSAMR | A*68:01 | 15% MNG3 MNG499 MNG814 | WHO I+II MNG6 MNG632 | Kelch-like protein 23 (KLHL23; Q8NBE8) | 21% | 3 3% | 0% 2% |
| ETASVLVNYR | A*66:01 A*68:01 | 15% MNG3 MNG632 MNG814 | WHO I-III MNG6 MNG734 | Intersectin-2 (ITSN2; Q9NZM3) | 24% | 6 19% | 11% 13% |
| EVASEIQPFLR | A*68:01 | 15% MNG3 MNG499 MNG814 | WHO I+II MNG6 MNG632 | Desmoplakin (DSP; P15924) | 97% | 1 34% | 56% 23% |
| FEGNVFMY | B*18:01 | 15% MNG1 MNG628 MNG661 | WHO I+II MNG6 MNG638 | Cell cycle control protein 50A (TMEM30A; Q9NV96) | 64% | 7 30% | 44% 18% |
| GLFQKLENI | A*02:01 | 15% MNG501 MNG666 MNG700 | WHO I+II MNG641 MNG673 | Desmoplakin (DSP; P15924) | 97% | 1 34% | 56% 32% |
| HSIGAVGPTGR | A*68:01 | 15% MNG3 MNG499 MNG814 | WHO I+II MNG6 MNG632 | Serine palmitoyl-transferase 3 (SPTLC3; Q9NUV7) | 76% | 0 20% | 33% 17% |
| IYNGKVTSI | A*24:02 | 15% MNG1 MNG635 MNG833 | WHO I+II MNG628 MNG661 | ATP-dependent RNA helicase DHX8 (DHX8; Q14562) | 58% | 6 23% | 11% 18% |
| LAFPGEMLL | C*03:03 C*12:03 | 15% MNG628 MNG661 MNG734 | WHO I-III MNG636 MNG666 | Neutral amino acid transporter A (SLC1A4; P43007) | 18% | 23 9% | 0% 3% |
| LPIGIALMF | B*51:01 | 15% MNG499 MNG632 MNG833 | WHO I+II MNG501 MNG682 | Multidrug and toxin extrusion protein 1 (SLC47A1; Q96FL8) | 70% | 1 5% | 33% 5% |
| LPIIANMI | B*51:01 | 15% MNG499 MNG612 MNG833 | WHO I-III MNG501 MNG642 | Armadillo repeat-containing X-linked protein 2 (ARMCX2; Q7L311) | 45% | 11 12% | 33% 11% |
| MEYFPTTRF | B*18:01 | 15% MNG1 MNG623 MNG638 | WHO I+II MNG6 MNG628 | Phospholipase D4 (PLD4; Q96BZ4) | 39% | 1 5% | 33% 4% |
| PYFDKPLFI | A*24:02 | 15% MNG628 MNG638 MNG833 | WHO I+II MNG635 MNG661 | Sodium leak channel non-selective protein (HSBP1; Q8IZF0) | 24% | 1 2% | 0% 1% |
| QTMSDQIIGR | A*68:01 | 15% MNG3 MNG499 MNG814 | WHO I+II MNG6 MNG632 | Heat shock factor-binding protein 1 (O75506) | 15% | 8 3% | 0% 3% |
| STITPTSTR | A*66:01 A*68:01 | 15% MNG3 MNG499 MNG734 | WHO I+III MNG6 MNG632 | Prolow-density lipoprotein receptor-related protein 1 (LRP1; Q07954) | 88% | 7 32% | 67% 38% |
| TPHDFIEHF | B*35:01 | 15% MNG624 MNG632 MNG833 | WHO I+II MNG628 MNG682 | G1/S-specific cyclin-D1 (CCND1; P24385) | 79% | 13 36% | 67% 39% |
| VALPVYLLI | B*51:01 | 15% MNG499 MNG612 MNG833 | WHO I-III MNG501 MNG702 | Phosphatidylinositol N-acetylgluco-saminyltransferase subunit P (PIGP; P57054) | 30% | 27 9% | 11% 2% |
| VAVGFPMMI | C*12:03 | 15% MNG628 MNG661 MNG734 | WHO I-III MNG636 MNG666 | Putative sodium-coupled neutral amino acid transporter 10 (SLC38A10; Q9HBR0) | 48% | 19 24% | 33% 19% |
| VEVQLPELY | B*18:01 B*44:03 | 15% MNG1 MNG636 MNG661 | WHO I+II MNG6 MNG638 | Voltage-gated potassium channel subunit beta-1 (KCNAB1; Q14722) | 21% | 9 5% | 11% 4% |
| YGYSNPKIL | C*03:04 C*12:03 | 15% MNG3 MNG636 MNG734 | WHO I-III MNG628 MNG661 | Transcription factor AP-1 (JUN; P05412) | 33% | 27 24% | 11% 26% |
| YNFEYSVVF | B*38:01 C*12:03 | 15% MNG628 MNG637 MNG734 | WHO I-III MNG636 MNG666 | Renin receptor (RENR; O75787) | 70% | 16 42% | 33% 41% |
| YPNAKVELV | B*51:01 B*51:08 | 15% MNG4 MNG501 MNG702 | WHO I+II MNG499 MNG702 | PDZ and LIM domain protein 4 (PDLIM4; P50479) | 28% | 17 8% | 0% 4% |
| YYASAFSMM | A*24:02 | 15% MNG628 MNG638 MNG833 | WHO I+II MNG635 MNG661 | Dolichyl-diphospho-oligosaccharide-protein glycosyl-transferase 48 kDa subunit (DDOST; P39656) | 58% | 0 39% | 44% 41% |
| YYKSTSSAF | A*24:02 | 15% MNG1 MNG635 MNG833 | WHO II MNG628 MNG638 | Galactose-3-O-sulfotransferase 4 (GAL3ST4; Q96RP7) | 30% | 9 6% | 11% 1% |
| DAFDGHAAR | A*33:01 A*34:01 A*66:01 A*68:01 | 15% MNG3 MNG636 MNG734 | WHO I-III MNG632 MNG642 | CDP-diacylglycerol-inositol 3-phospha-tidyltransferase (CDIPT; O14735) | 28% | 6 9% | 0% 6% |
| DSERFFIRY | A*01:01 | 15% MNG4 MNG7 MNG646 | WHO I MNG5 MNG624 | Tubulin-specific chaperone cofactor E-like protein (TBCEL; Q5QJ74) | 18% | 30 7% | 11% 9% |
| EAENTLQSFR | A*68:01 | 15% MNG3 MNG499 MNG814 | WHO I+II MNG6 MNG632 | Vimentin (VIM; P08670) | 100% | 1 88% | 100% 96% |
| EAIFLEVKY | A*26:01 A*29:02 B*35:01 | 15% MNG624 MNG679 MNG833 | WHO I+II MNG634 MNG682 | Vacuolar protein sorting-associated protein 13D (VPS13D; Q5THJ4) | 61% | 1 30% | 33% 35% |
| EVFAGSGTSGQR | A*66:01 A*68:01 | 15% MNG3 MNG632 MNG814 | WHO I-III MNG6 MNG734 | Procollagen C-endopeptidase enhancer 1 (PCOLCE; Q15113) | 21% | 1 1% | 0% 1% |
| EVPSFTMGR | A*34:01 A*66:01 A*68:01 | 15% MNG3 MNG642 MNG814 | WHO I+II MNG6 MNG734 | PRKR-interacting protein 1 (PRKRIP1; Q9H875) | 15% | 5 1% | 0% 0.5% |
| FFNIPQIQY | A*29:02 | 15% MNG1 MNG673 MNG682 | WHO I+II MNG623 MNG679 | 28S ribosomal protein S25, mitochondrial (MRPS25; P82663) | 15% | 12 4% | 0% 5% |
| FLMEMGFRM | A*02:01 | 15% MNG6 MNG628 MNG673 | WHO I-III MNG612 MNG638 | Mediator of RNA polymerase II transcription subunit 18 (MED18; Q9BUE0) | 24% | 13 7% | 11% 4% |
| FVFGENMVER | A*68:01 | 15% MNG3 MNG499 MNG814 | WHO I+II MNG6 MNG632 | Ran-binding protein 3-like (RANBP3L; Q86VV4) | 24% | 1 1% | 0 1% |
| GAFGLPITV | C*02:02 C*12:03 | 15% MNG636 MNG666 MNG734 | WHO I+III MNG661 MNG702 | Glutathione S-transferase kappa 1 (GSTK1; Q9Y2Q3) | 24% | 10 27% | 11% |
| GLLPLLREA | A*02:01 | 15% MNG641 MNG673 MNG734 | WHO I-III MNG666 MNG702 | Stabilin-1 (STAB1; Q9NY15) | 79% | 34 34% | 56% |
| HTNDTIGSVR | A*66:01 A*68:01 | 15% MNG3 MNG632 MNG814 | WHO I-III MNG6 MNG734 | Probable ubiquitin carboxyl-terminal hydrolase FAF-X / FAF-Y (USP9X/Y; Q93008 / O00507) | 82% / 64% | 18 53% / 41% | 67% / 44% 57%/ 41% |
| NLMGKTSER | A*34:01 A*66:01 A*68:01 | 15% MNG3 MNG632 MNG734 | WHO I-III MNG6 NBG642 | Transcription factor 12 (TCF12; Q99081) | 61% | 3 43% | 33% 29% |
| RVYDIPPKF | A*30:02 A*32:01 B*15:35 C*12:03 | 15% MNG7 MNG634 MNG661 | WHO I+II MNG632 MNG642 | Cytochrome b-245 heavy chain (CYBB; P04839) | 88% | 4 34% | 56% 32% |
| SLSLENVLYY | A*29:02 B*15:01 | 15% MNG1 MNG673 MNG682 | WHO I+II MNG623 MNG679 | Sortilin-related receptor (SORL1; Q92673) | 45% | 14 23% | 11% 17% |
| SPNNFLSYY | B*35:01 | 15% MNG628 MNG634 MNG833 | WHO I+II MNG632 MNG682 | G1/S-specific cyclin-D1 (CCND1; P24385) | 79% | 8 36% | 67% 39% |
| SVDSNLLSDY | A*01:01 | 15% MNG3 MNG624 MNG702 | WHO I MNG7 MNG646 | Oxidative stress-induced growth inhibitor 2 (OSGIN2; Q9Y236) | 42% | 41 12% | 33% 7% |
| SVIEGVSRSR | A*66:01 A*68:01 | 15% MNG3 MNG632 MNG814 | WHO I-III MNG6 MNG734 | BTB/POZ domain-containing protein 9 (BTBD9; Q96Q07) | 27% | 13 12% | 0% 6% |
| SYFPTVPGVYI | A*24:02 | 15% MNG1 MNG635 MNG833 | WHO I+II MNG5 MNG638 | Filamin-B (FLNB; O75369) | 94% | 7 46% | 67% 60% |
| TEEPLKQSF | B*18:01 | 15% MNG1 MNG628 MNG661 | WHO I+II MNG6 MNG638 | GRAM domain-containing protein 3 (GRAMD3; Q96HH9) | 42% | 9 12% | 11% 13% |
| VELLMHNDY | B*18:01 | 15% MNG1 MNG628 MNG661 | WHO I+II MNG6 MNG638 | Importin subunit alpha-5/6/7 (KPNA1/5/6; P52294 / O15131 / O60684) | 73% / 58 % / 64% | 12 52% / 45% / 46% | 78% / 67% / 78% 53% / 45% / 46% |
| VGVDFALKV | C*12:03 | 15% MNG628 MNG661 MNG734 | WHO I-III MNG636 MNG666 | Ras-related protein Rab-7L1 (RAB7L1; O14966) | 27% | 12 4% | 11% / 2% |

**Supplementary Table 7** Meningioma-exclusive HLA class II-presented peptides derived from meningioma-associated HLA presentation hotspots. UniProt correspondence, protein frequency on meningiomas, peptide sequences, frequency of positive patients, protein frequency on non-meningeal tumors and on dura/benign samples. The number of positive non-meningeal tumors was based on n=593 HLA class II peptidome datasets

| **Antigen (UniProt accession)** | **Protein frequency on meningiomas** | **Peptide sequence** | **Frequency of positive patients** | | **Protein frequency on non-meningeal tumors**  **Peptide-positive non-meningeal tumors** | **Protein frequency on dura (n=9) / benign samples (n=364)** |
| --- | --- | --- | --- | --- | --- | --- |
| Unconventional myosin-Ic (MYI1C; O00159) | 49% | ENQLKYLTRLLSVE  ENQLKYLTRLLSVEG  NQLKYLTRLLSVEG  TENQLKYLTRLLSVE  TENQLKYLTRLLSVEG | WHO  12%  MNG623  MNG702  6%  MNG623  6%  MNG623  3%  12%  MNG623  MNG666 | I+II  MNG6  MNG666  MNG6  MNG6  MNG623  MNG6  MNG635 | 11%  1  5  0  0  5 | 0% / 17% |
| E3 ubiquitin-protein ligase MARCH6 (MARCH6; O60337) | 15% | DQTPLFYPWQDWALGVLH  TPLFYPWQDWALGVLH | WHO  15%  MNG642  MNG673  6%  MNG833 | I+II MNG637 MNG666  MNG833  MNG637 | 2% 0  0 | 0% / 2% |
| Slit homolog 2 protein (SLIT2; O94813) | 52% | DHIAVELYRGRVRAS  DHIAVELYRGRVRASYD  DKDHIAVELYRGRVRASYD  HIAVELYRGRVRAS  HIAVELYRGRVRASYD  KGDKDHIAVELYRGRVRASY | WHO  12%  MNG634  MNG734  6%  MNG641  6%  MNG641  3%  3%  3% | I-III  MNG6  MNG641  MNG6  MNG6  MNG636  MNG6  MNG636 | 7%  0  0  0  0  1  0 | 22% / 7% |
| EGF-containing fibulin-like extracellular matrix protein 2 (EFEMP2; O95967) | 45% | DVFQIQATSVYPG  ERSVPADVFQIQATSVYPG  ITSERSVPADVFQIQATSVYPGAYN  SVPADVFQIQATSVYPG  VPADVFQIQATSVYPG | WHO  9%  MNG638  3%  3%  6%  MNG638  24%  MNG6  MNG636  MNG641  MNG734 | I-III MNG6  MNG734  MNG636  MNG814  MNG636  MNG3  MNG624  MNG638  MNG682 | 8%  0  0  1  1  12 | 33% / 6% |
| Coagulation factor XIII A chain (F13A1; P00488) | 79% | LSANITFYTGVPKAEF  LSANITFYTGVPKAEFK  SANITFYTGVPKAEF | WHO  9%  MNG634  6%  MNG734  6% MNG734 | I+III  MNG6  MNG636  MNG636  MNG612 | 23%  1  0  0 | 44% / 32% |
| Antithrombin-III (SERPINC1; P01008) | 85% | SDQIHFFFAKLN | WHO  15%  MNG612  MNG641 | I-III  MNG6  MNG636  MNG734 | 50% 1 | 89% / 48% |
| Insulin-like growth factor II (IGF2; P01344) | 70% | PAHGGAPPEM  PAHGGAPPEMA  PAHGGAPPEMAS  PAHGGAPPEMASN | WHO  3%  3%  6%  MNG702  33%  MNG624  MNG638  MNG666  MNG679  MNG833 | I+II  MNG637  MNG637  MNG637  MNG3  MNG637  MNG641  MNG673  MNG702 | 16% 0 1 0  8 | 44% / 18% |
| Fibronectin (FN1; P02751) | 94% | LTPGVEYVYTIQVLRDGQERDAPI  TPGVEYVYTIQVLRDG  TPGVEYVYTIQVLRDGQE  TPGVEYVYTIQVLRDGQER  TPGVEYVYTIQVLRDGQERDAPI  YVYTIQVLRDGQERDAPI  VYTIQVLRDGQERDAPI  VGQQMIFEEHGFRRTTPPT  GQQMIFEEHGFRRTTPP  GQQMIFEEHGFRRTTPPT  QQMIFEEHGFRRTTPP  IFEEHGFRRTTPP | WHO  3%  6%  MNG679  9%  MNG638  3%  3%  3%  3%  6%  MNG638  12%  MNG635  MNG641  6%  MNG638  12%  MNG612  MNG734  3% | I-III  MNG632  MNG638  MNG1  MNG679  MNG638  MNG632  MNG642  MNG642  MNG635  MNG612  MNG638  MNG635  MNG6  MNG635  MNG734 | 54%  0  6  1  1  0  2  3  1  2  1  9  0 | 100% / 65% |
| Collagen alpha-2(V) chain (COL5A2; P05997) | 36% | GNVGKTVFEYRTQNVAR  VGKTVFEYRTQNVAR  VARLPIIDLAPVDVGGTD  ARLPIIDLAPVDVGGTD  RLPIIDLAPVDVGGTD  LPIIDLAPVDVGGT  LPIIDLAPVDVGGTD | WHO  6%  MNG638  12%  MNG636  MNG734  9%  MNG642  9%  MNG499  6%  MNG642  3%  9%  MNG499 | I-III  MNG636  MNG612  MNG638  MNG499  MNG700  MNG1  MNG642  MNG499  MNG499  MNG1  MNG642 | 11%  3  10  13  21  28  25  35 | 11% / 3% |
| Secretogranin-2 (SCG2; P13521) | 30% | LSDDVSKVIAYLKRLVNAAGSG  SDDVSKVIAYLKRLVNAAG  SDDVSKVIAYLKRLVNAAGSG  DDVSKVIAYLKRLVNAA  DDVSKVIAYLKRLVNAAG  DDVSKVIAYLKRLVNAAGSG  DVSKVIAYLKRLVNAA  DVSKVIAYLKRLVNAAG  VSKVIAYLKRL  VSKVIAYLKRLVNA  VSKVIAYLKRLVNAA  VSKVIAYLKRLVNAAG  SKVIAYLKRLVNAA  SGYPKTPGRAGTEALPDG  YPKTPGRAGTEALPDG  TPGRAGTEALPDG | WHO  3%  3%  3%  3%  9%  MNG6  6%  MNG702  6%  MNG6  9%  MNG6  3%  6%  MNG6  15%  MNG6  MNG641  15%  MNG6 MNG641  3%  3%  9%  MNG682  9%  MNG682 | I-III  MNG4  MNG4  MNG4  MNG4  MNG4  MNG702  MNG4  MNG4  MNG4  MNG702  MNG6  MNG4  MNG4  MNG635  MNG702  MNG4 MNG635  MNG702  MNG6  MNG682  MNG6  MNG734  MNG6  MNG734 | 9%  1  2  1  1  3  1  4  8  0  4  16  16  2  0  6  12 | 0% / 5% |
| Prolyl 4-hydroxylase subunit alpha-1 (P4HA1; P13674) | 24% | DKVSVLDYLSYA  DKVSVLDYLSYAVYQ  DKVSVLDYLSYAVYQQ  DKVSVLDYLSYAVYQQG | WHO  6%  MNG638  9%  MNG7  9%  MNG635  3% | I+II  MNG501  MNG3  MNG638  MNG501  MNG638  MNG3 | 7%  5  7  9  11 | 0% / 3% |
| Inter-alpha-trypsin inhibitor heavy chain H2 (ITIH2; P19823) | 73% | FEIPINGLSE  FEIPINGLSEF    FEIPINGLSEFVD | WHO 6% MNG702 18% MNG501 MNG702 MNG833 6% MNG501 | I+II MNG638  MNG6 MNG638 MNG814   MNG702 | 35%  3  1  0 | 33% / 32% |
| Macrophage scavenger receptor types I and II (MSR1; P21757) | 15% | EVFMEHMSNMEKRIQH  MEHMSNMEKRIQH  MEHMSNMEKRIQHILD  RIQHILDMEANLMDTE  IQHILDMEANLM  IQHILDMEANLMD  IQHILDMEANLMDT  IQHILDMEANLMDTE | WHO  3%  3%  3%  6%  MNG734  3%  3%  3%  3% | I-III  MNG642  MNG635  MNG6  MNG638  MNG734  MNG734  MNG638  MNG638 | 3%  3  2  0  1  0  0  0  1 | 0% / 1% |
| Cellular retinoic acid-binding protein 1 (CRABP1; P29762) | 27% | INFKVGEGFEEETVD  INFKVGEGFEEETVDG  KVGEGFEEETVD | WHO  15%  MNG623  MNG661  3%  3% | I+II  MNG7  MNG642  MNG679  MNG642  MNG7 | 2%  2  0  0 | 0% / 1% |
| Replication factor C subunit 4 (RFC4; P35249) | 18% | KDRGVAASAGSSGENKKAKPVP | WHO  18%  MNG499  MNG634  MNG682 | I+II  MNG7  MNG628  MNG638 | 1%  3 | 11% / 1% |
| Disintegrin and metalloproteinase domain-containing protein 17 (ADAM17; P78536) | 24% | HVETLLTFSALKRH  HVETLLTFSALKRHF  HVETLLTFSALKRHFK  TSTHVETLLTFSALKRHFK  VETLLTFSALKRH  VETLLTFSALKRHF  VETLLTFSALKRHFK | WHO  3%  3%  15%  MNG612  MNG641  3%  6%  MNG734  9%  MNG641  12%  MNG634  MNG734 | I-III  MNG734  MNG641  MNG6  MNG634  MNG734  MNG641  MNG641  MNG6  MNG734  MNG612  MNG641 | 6%  1  0  3  0  0  1  3 | 0% / 3% |
| Probable cation-transporting ATPase 13A5 (ATP13A5; Q4VNC0) | 18% | VDSCKFGTSVSNIIKP | WHO  18% MNG612  MNG641  MNG734 | I-III MNG6  MNG634  MNG642 | 0.3%  1 | 0% / 1% |
| Tetratricopeptide repeat protein 27 (TTC27; Q6P3X3) | 18% | EIAIILGICTNFQKN | WHO  15% MNG636  MNG682 | I-III  MNG624  MNG638  MNG734 | 2% 1 | 0% / 2% |
| Chondroitin sulfate proteoglycan 4 (CSPG4; Q6UVK1) | 24% | DFIYVDIFEGHLRA  DFIYVDIFEGHLRAVV  DFIYVDIFEGHLRAVVE  FIYVDIFEGHLRA  FIYVDIFEGHLRAV  FIYVDIFEGHLRAVV  FIYVDIFEGHLRAVVE  IYVDIFEGHLRA  IYVDIFEGHLRAVV  IYVDIFEGHLRAVVE | WHO  9%  MNG634  3%  6%  MNG734  15%  MNG612  MNG641  3%  6%  MNG734  3%  18%  MNG612  MNG636  MNG734  6%  MNG734  6%  MNG734 | I-III  MNG6  MNG734  MNG634  MNG612  MNG6  MNG636  MNG734  MNG734  MNG612  MNG734  MNG6  MNG634  MNG641  MNG634  MNG634 | 8%  2  0  1  5  1  2  0  4  2  0 | 0% / 6% |
| Protocadherin Fat 4 (FAT4; Q6V0I7) | 70% | GGNSQFTINPSTGQIIT  GNSQFTINPSTGQII  GNSQFTINPSTGQIIT  IGGNSQFTINPSTGQIIT | WHO  3%  3%  9%  MNG666  6%  MNG673 | I+II  MNG702  MNG702  MNG3  MNG702  MNG635 | 4%  0  0  0  0 | 11% / 20% |
| Interleukin-34 (IL34; Q6ZMJ4) | 18% | DKLQYRSRLQYMKHY  LRDKLQYRSRLQYMKHY  RDKLQYRSRLQYMKHY | WHO  3%  15%  MNG3  MNG673  6%  MNG833 | I+II  MNG666  MNG1  MNG666  MNG702  MNG666 | 0%  0  0  0 | 0% / 1% |
| Thrombospondin type-1 domain-containing protein 4 (THSD4; Q6ZMP0) | 82% | EMYKSNNYLALRSRSGRSIIN  KSNNYLALRSRSG  KSNNYLALRSRSGR  KSNNYLALRSRSGRS  KSNNYLALRSRSGRSI  KSNNYLALRSRSGRSIIN  KSNNYLALRSRSGRSIING  SNNYLALRSRSG  SNNYLALRSRSGR  SNNYLALRSRSGRS  SNNYLALRSRSGRSI  SNNYLALRSRSGRSIIN  YKSNNYLALRSRSGRS  YKSNNYLALRSRSGRSI  YKSNNYLALRSRSGRSIIN | WHO  3%  3%  3%  9%  MNG636  15%  MNG624  MNG702  3%  3%  3%  3%  3%  9%  MNG682  6%  MNG702  3%  3%  3% | I+III  MNG682  MNG682  MNG682  MNG624  MNG682  MNG6  MNG682  MNG734  MNG682  MNG682  MNG682  MNG682  MNG682  MNG624  MNG734  MNG3  MNG682  MNG682  MNG682 | 9%  0  1  4  5  6  0  1  2  2  1  4  1  1  0  0 | 56% / 12% |
| Transmembrane protease serine 9 (TMPRSS9; Q7Z410) | 21% | SDYHRTLTPTLEALLH | WHO  21%  MNG635 MNG637 MNG666 | I+II MNG499 MNG636 MNG646 MNG682 | 1% 1 | 0% / 1% |
| Epidermal growth factor-like protein 6 (EGFL6; Q8IUX8) | 27% | GKGKTGEIAVDGVLLVSG  GKTGEIAVDGVLLVSG  GKTGEIAVDGVLLVSGL  KSIIFEAERGKGKTG  KTGEIAVDGVLLVS  KTGEIAVDGVLLVSG  KTGEIAVDGVLLVSGL  TKSIIFEAERGKGKTG | WHO  3%  9%  MNG7  6%  MNG637  6%  MNG638  3%  12%  MNG7  MNG646  12%  MNG7  MNG637  3% | I+II  MNG7  MNG5  MNG624  MNG7  MNG6  MNG7  MNG5  MNG624  MNG5  MNG624  MNG6 | 1%  1  1  3  0  0  2  4  0 | 0% / 1% |
| E3 ubiquitin-protein ligase UBR1 (UBR1; Q8IWV7) | 21% | TKDQDLIKQYNTLIEEMLQV | WHO  18% MNG6 MNG641 MNG682 | I+II MNG3 MNG499 MNG702 | 1% 2 | 0% / 1% |
| Phosphatidyl-inositol 3,4,5-trisphosphate-dependent Rac exchanger 1 protein (PREX1; Q8TCU6) | 24% | LRNDFKLVENILAKR  LRNDFKLVENILAKRL | WHO  15%  MNG634  MNG637  3% | I+II  MNG6  MNG636  MNG641  MNG637 | 3%  5  2 | 0% / 3% |
| Protein KIAA1199 (KIAA1199; Q8WUJ3) | 70% | DPLKPREPAIIRHFIAYKNQD  DPLKPREPAIIRHFIAYKNQDH  DPLKPREPAIIRHFIAYKNQDHG  KPREPAIIRHFIAYKNQD  KPREPAIIRHFIAYKNQDH  KPREPAIIRHFIAYKNQDHG  EPAIIRHFIAYK  EPAIIRHFIAYKN  EPAIIRHFIAYKNQ  EPAIIRHFIAYKNQD  IIRHFIAYKNQDHG  IRHFIAYKNQDHG  IPDNSIVLMASKGRYV  IPDNSIVLMASKGRYVS  IPDNSIVLMASKGRYVSR  PDNSIVLMASKGRYVS | WHO  3%  3%  9%  MNG641  12%  MNG636  MNG734  6%  MNG734  3%  3%  6%  MNG734  6%  MNG734  3%  3%  3%  36%  MNG3  MNG7  MNG636  MNG641  MNG666  MNG734  45%  MNG3  MNG7  MNG634  MNG636  MNG641  MNG666  MNG833  27%  MNG6  MNG636  MNG641  MNG702  3% | I-III  MNG638  MNG638  MNG638  MNG734  MNG6  MNG641  MNG6  MNG734  MNG734  MNG641  MNG641  MNG734  MNG642  MNG642  MNG1  MNG6  MNG634  MNG638  MNG642  MNG702  MNG1  MNG6  MNG612  MNG635  MNG638  MNG642  MNG702  MNG833  MNG1  MNG634  MNG638  MNG666  MNG734  MNG702 | 10%  0  0  0  0  0  0  0  0  0  0  0  0  0  1  0  0 | 11% / 5% |
| Cortactin-binding protein 2 (CTNNBP2; Q8WZ74) | 18% | QKKLEMEKLQLQALEQEHKK | WHO  18 %  MNG636 MNG682 MNG702 | I MNG499  MNG679 MNG700 | 1% 1 | 0% / 1% |
| SLIT and NTRK-like protein 6 (SLIRK6; Q9H5Y7) | 18% | NDSRMSTKTTSILKLP | WHO  18%  MNG612  MNG641  MNG682 | I-III  MNG6  MNG634  MNG642 | 2% 8 | 0% / 2% |
| Stabilin-1 (STAB1; Q9NY15) | 85% | DELARIRAHRQLVFR  DELARIRAHRQLVFRYH  ELARIRAHRQLVFRYH  GLVPQIEAATAYTIFVPT  IEAATAYTIFVPT  LARIRAHRQLVFR  LARIRAHRQLVFRYH  LVPQIEAATAYTIF  LVPQIEAATAYTIFVP  LVPQIEAATAYTIFVPT  VPQIEAATAYTIF  VPQIEAATAYTIFV  VPQIEAATAYTIFVP  VPQIEAATAYTIFVPT | WHO  9%  MNG666  15%  MNG666  MNG702  15%  MNG3  MNG702  3%  3%  15%  MNG3  MNG702  15%  MNG3  MNG702  3%  3%  12%  MNG624  MNG700  3%  3%  6%  MNG638  9%  MNG636 | I+II  MNG1  MNG702  MNG3  MNG673  MNG833  MNG1  MNG666  MNG833  MNG638  MNG638  MNG1  MNG666  MNG833  MNG1  MNG666  MNG833  MNG638  MNG638  MNG623  MNG638  MNG638  MNG638  MNG636  MNG623  MNG638 | 32%  3  5  2  1  0  4  1  0  3  6  0  2  4  4 | 44% / 32% |
| Carboxypepti-dase A4 (CPA4; Q9UI42) | 45% | IVSDYQRDPAITS  SREWISQATAIWTARK  TARKIVSDYQRDPA  TARKIVSDYQRDPAI  TARKIVSDYQRDPAIT  WTARKIVSDYQRDPA | WHO  3%  3%  9%  MNG624  3%  3%  3% | I+II  MNG702  MNG638  MNG661  MNG642  MNG624  MNG624  MNG642 | 1%  0  0  1  0  0  0 | 22% / 0% |
| Carboxypepti-dase Q (CPQ; Q9Y646) | 48% | AGVPGASLLDDL | WHO  15%  MNG632  MNG666 | I+II  MNG499  MNG642  MNG673 | 10% 0 | 22% / 7% |
| Olfactomedin-like protein 2B (OLFM2B; Q68BL8) | 55% | FSQEVIVLSKLNAAD  FSQEVIVLSKLNAADL  GAFYYNRAFTRNII  GAFYYNRAFTRNIIK  GFSQEVIVLSKLNAAD  GFSQEVIVLSKLNAADL  NGAFYYNRAFTRNII  NGAFYYNRAFTRNIIK  SQEVIVLSKLNAAD  SQEVIVLSKLNAADL  VIVLSKLNAAD  VIVLSKLNAADL  VYNGAFYYNRAFTRNIIK  YNGAFYYNRAFTRNII  YNGAFYYNRAFTRNIIK  YYNRAFTRNII | WHO  12%  MNG635  MNG661  9%  MNG635  9%  MNG501  6%  MNG700  3%  6%  MNG641  6%  MNG642  6%  MNG642  9%  MNG641  15%  MNG635  MNG661  3%  3%  3%  12%  MNG501  MNG642  9%  MNG638  6%  MNG642 | I+II  MNG623  MNG641  MNG623  MNG641  MNG3  MNG642  MNG642  MNG623  MNG623  MNG638  MNG638  MNG635  MNG661  MNG623  MNG641  MNG702  MNG628  MNG702  MNG642  MNG3  MNG638  MNG501  MNG642  MNG638 | 11%  2  6  4  0  1  3  0  0  3  6  1  2  0  1  2  0 | 11% / 1% |
| Inter-alpha-trypsin inhibitor heavy chain H5 (ITIH5; Q86UX2) | 55% | GHKKQRTYLRTITILINKPE  KQRTYLRTITILINKPE | WHO  6%  MNG679  15%  MNG2  MNG623 | I+II  MNG623  MNG1  MNG5  MNG679 | 8%  0  1 | 22% / 12% |
| Immunoglobulin superfamily member 21 (IGSF21; Q96ID5) | 21% | APKGPKIVMTPSRARVGDT | WHO  15%  MNG3  MNG702 | I+II  MNG1  MNG666  MNG833 | 1% 1 | 0% / 1% |
| CUB and sushi domain-contain-ing protein 1 (CSMD1; Q96PZ7) | 18% | KSPVCKSKGVREVNETVTKTPVP | WHO  15%  MNG499  MNG641 | I+II  MNG7  MNG638  MNG666 | 1% 1 | 0% / 1% |
| Hermansky-Pudlak syndrome 3 protein (HPS3; Q969F9) | 21% | ERGLIFYINHSLYE  ERGLIFYINHSLYEN  KYERGLIFYINHSLY | WHO  3%  9%  MNG634  12%  MNG501  MNG814 | I+II  MNG7  MNG3  MNG638  MNG3  MNG702 | 2%  0  4  3 | 11% / 1% |
| Fibromodulin (FMOD; Q06828) | 94% | MPGPLPRSLRELHLDHNQI  MPGPLPRSLRELHLDHNQISR  MPGPLPRSLRELHLDHNQISRVPN  NLTRMPGPLPRSLRE  NLTRMPGPLPRSLRELH  NNLTRMPGPLPRSLRE  NNLTRMPGPLPRSLRELH | WHO  9%  MNG642  9%  MNG646  3%  6%  MNG646  3%  6%  MNG646  6%  MNG646 | I+II  MNG499  MNG700  MNG499  MNG700  MNG499  MNG635  MNG635  MNG635  MN635 | 26%  10  21  18  0  0  0  0 | 56% / 18% |
| EGF-containing fibulin-like extracellular matrix protein 1 (EFEMP1; Q12805) | 61% | GRNNFVIRRNPADPQR  GRNNFVIRRNPADPQRIP  GRNNFVIRRNPADPQRIPS  GRNNFVIRRNPADPQRIPSN  GRNNFVIRRNPADPQRIPSNP  GRNNFVIRRNPADPQRIPSNPS  NFVIRRNPADPQR  NFVIRRNPADPQRIPS  NNFVIRRNPAD  NNFVIRRNPADPQR  NNFVIRRNPADPQRIPS  NNFVIRRNPADPQRIPSNPS  PQRIPSNPS  TGRNNFVIRRNPADPQR  TGRNNFVIRRNPADPQRIPS  TGRNNFVIRRNPADPQRIPSN  TGRNNFVIRRNPADPQRIPSNPS  TGRNNFVIRRNPADPQRIPSNPSH  VIRRNPADPQR  VIRRNPADPQRIPS | WHO  9%  MNG702  3%  9%  MNG666  3%  3%  3%  9%  MNG666  12%  MNG682  MNG833  6%  MNG702  6%  MNG702  12%  MNG682  MNG833  3%  6%  MNG702  3%  3%  3%  3%  3%  3%  6% | I+II  MNG682  MNG833  MNG702  MNG682  MNG702  MNG702  MNG628  MNG702  MNG628  MNG702  MNG628  MNG682  MNG628  MNG702  MNG702  MNG628  MNG666  MNG666  MNG702  MNG702  MNG702  MNG628  MNG628  MNG702 | 11%  0  0  1  0  0  0  1  3  0  0  2  0  0  0  0  0  0  0  0  1 | 33% / 16% |
| Collagen alpha-3(IX) chain (COL9A3; Q14050) | 24% | EQIAQLAAHLRKPL  EQIAQLAAHLRKPLAPG  GGMISEQIAQLAAHLRKPLAPG  IAQLAAHLRKPLAPG  ISEQIAQLAAHLRKP  ISEQIAQLAAHLRKPL  ISEQIAQLAAHLRKPLA  ISEQIAQLAAHLRKPLAP  ISEQIAQLAAHLRKPLAPG  QIAQLAAHLRKP  QIAQLAAHLRKPL  SEQIAQLAAHLRKP  SEQIAQLAAHLRKPL  SEQIAQLAAHLRKPLA  SEQIAQLAAHLRKPLAP | WHO  6%  MNG734  3%  6%  MNG734  6%  MNG702  9%  MNG636  18%  MNG612  MNG638  MNG734  6%  MNG734  15%  MNG612  MNG641  21%  MNG501  MNG636  MNG641  3%  3%  6%  MNG734  15%  MNG612  MNG641  3%  3% | I-III  MNG636  MNG734  MNG641  MNG501  MNG612  MNG734  MNG6  MNG636  MNG641  MNG641  MNG6  MNG636  MNG734  MNG6  MNG612  MNG638  MNG734  MNG734  MNG734  MNG636  MNG6  MNG636  MNG734  MNG734  MNG734 | 2%  1  0  0  1  2  6  3  6  6  1  1  2  4  1  1 | 0% / 2% |
| Nidogen-2 (NID2; Q14112) | 73% | DTSPAVLGLAARYVRAGFPR  EDTSPAVLGLAARYVRAGFPR  LPSGELNTFQAVLASDG  LPSGELNTFQAVLASDGSDSYAL  SPAVLGLAARYVRAG  SPAVLGLAARYVRAGFP  SPAVLGLAARYVRAGFPR  TSPAVLGLAARYVRAG | WHO  3%  12%  MNG666  MNG833  15%  MNG6  MNG641  3%  6%  MNG638  9%  MNG3  9%  MNG702  3% | I-III  MNG1  MNG1  MNG673  MNG3  MNG638  MNG734  MNG6  MNG3  MNG1  MNG833  MNG1  MNG833  MNG638 | 14%  0  0  2  0  1  0  0  1 | 11% / 13% |
| Importin subunit beta-1 (KPNB1; Q14974) | 18% | KTTLVIMERLQQ  KTTLVIMERLQQVL  KTTLVIMERLQQVLQ | WHO  3%  3%  15%  MNG623  MNG666 | I+II  MNG6  MNG623  MNG6  MNG641  MNG702 | 11%  1  3  16 | 0% / 8% |
| Splicing factor 1 (SF1; Q15637) | 15% | GVAGMPPFGMPPAPPPPPPQN  MGMGVAGMPPFGMPPAPPPPPPQN | WHO  12%  MNG628  MNG702  6%  MNG702 | I+II  MNG6  MNG638  MNG666 | 6%  13  20 | 0% / 2% |
| Proteoglycan 4 (PRG4; Q92954) | 42% | GGLTGQIVAALSTAKYKNWPE  GLTGQIVAALSTAKYKN  GLTGQIVAALSTAKYKNWPE  GQIVAALSTAKYKN  TGQIVAALSTAKYK  TGQIVAALSTAKYKN | WHO  3%  6%  MNG638  6%  MNG666  3%  3%  9%  MNG638 | I+II  MNG6  MNG3  MNG6  MNG3  MNG642  MNG3  MNG642 | 3%  0  0  1  0  0  0 | 0% / 5% |

**Supplementary table 8** Established TAA and CTA identified as meningioma-exclusive antigens (represented by meningioma-exclusive peptides on ≥ 2 tumors). Peptide sequence, HLA restriction, frequency of positive patients, protein frequency on meningiomas, non-meningeal tumors, and on dura/benign samples. HLA restrictions not passing manual assessment as quality control are indicated in italic. The number of positive non-meningeal tumors was based on n=841 HLA class I and n=593 HLA class II peptidome datasets. The frequency of positive benign HLA peptidomes was calculated from n=9 tumor-free dura samples and n=418 (HLA class I) or n=364 (HLA class II) benign human specimens. Meningioma exclusivity of HLA class II-restricted peptides was evaluated for the exact sequence match with none of the peptides arising from a tumor-associated presentation hotspot

| **Peptide sequence** | **HLA restriction** | **Frequency of positive patients** | | | **Protein frequency on meningiomas** | **Protein frequency on non-meningeal tumors Peptide-positive non-meningeal tumors** | **Protein frequency on dura / benign samples** |
| --- | --- | --- | --- | --- | --- | --- | --- |
| **Meningioma-exclusive HLA class I-presented antigens derived from established TAA and CTA** | | | | | | | |
| Protein SSX5/SSX9 (SSX5/9; O60225 / Q7RTT3) | | | | |  |  |  |
| Supplementary Table 3 | | | | | | | |
| Probable ATP-dependent RNA helicase DDX43/54 (DDX43/53; Q9NXZ2 / Q86TM3) | | | | | 6% / 6% | 0.5% / 0.5% | 0% / 0% 0.5% / 0.5% |
| YLMPGFIHL | A*02:01; C*17:01 | 6% | MNG628 | MNG641 |  | 0 |  |
| **Meningioma-exclusive HLA class I ligands derived from established TAA and CTA** | | | | | | | |
| Neurofibromin (NF1; P21359) | | | | | 64% | 25% | 56% / 22% |
| LPYLFHVV | B*51:01 | 12% | MNG499 MNG642 | MNG612 MNG833 |  | 13 |  |
| NYPDEFTKL | A*24:02 | 12% | MNG1 MNG635 | MNG628 MNG661 |  | 8 |  |
| AETVLADRF | B*44:03 | 6% | MNG1 | MNG636 |  | 2 |  |
| DEFDQRILY | B*18:01 | 6% | MNG1 | MNG661 |  | 5 |  |
| AVIAFRSSY | A*30:02 | 6% | MNG7 | MNG632 |  | 3 |  |
| DYAELIVKF | A*24:02 | 6% | MNG628 | MNG833 |  | 5 |  |
| Catenin beta-1 (CTNNB1; P35222) | | | | | 100% | 75% | 100% / 72% |
| ATVGLIRNL | A*32:01; *B*13:02 C*02:02* | 9% | MNG634 MNG702 | MNG635 |  | 22 |  |
| KTNVKFLAI | C*15:02 | 6% | MNG499 | MNG501 |  | 7 |  |
| TYTYEKLLW | A*23:01; A*24:02 | 6% | MNG638 | MNG679 |  | 9 |  |
| Platelet-derived growth factor receptor beta (PDGFRB; P09619) | | | | | 70% | 21% | 33% / 16% |
| YMDLVGFSY | A*29:02 | 9% | MNG623 MNG682 | MNG673 |  | 1 |  |
| MPYHIRSI | B*51:01 | 9% | MNG499 | MNG833 |  | 7 |  |
| Regulator of G-protein signaling 5 (RGS5; O15539) | | | | | 61% | 29% | 11% / 18% |
| KAKQIYEEF | *B*15:35*; B*57:01 | 9% | MNG3 MNG673 | MNG642 |  | 11 |  |
| KIKSPAKMAEK | A*03:01 | 6% | MNG636 | MNG814 |  | 15 |  |
| Mothers against decapentaplegic homolog 3 (SMAD3; P84022) | | | | | 33% | 11% | 11% / 12% |
| HASQPSMTV | B*51:01; C*15:02 | 9% | MNG499 MNG833 | MNG702 |  | 11 |  |
| Werner syndrome ATP-dependent helicase (WRN; Q14191) | | | | | 24% | 4% | 11% / 4% |
| ILQDLQPFL | A*02:01 | 9% | MNG612 MNG673 | MNG638 |  | 5 |  |
| Cytochrome P450 1B1 (CYP1B1; Q16678) | | | | | 73% | 27% | 89% / 41% |
| NRNFSNFIL | B*39:01; B*39:31 | 6% | MNG666 | MNG734 |  | 0 |  |
| RVMIFSVGK | A*03:01 | 6% | MNG636 | MNG666 |  | 3 |  |
| SMMRNFFTR | A*31:01 | 6% | MNG623 | MNG635 |  | 2 |  |
| Zinc phosphodiesterase ELAC protein 2 (ELAC2; Q9BQ52) | | | | | 61% | 30% | 22% / 22% |
| EISSPAVER | A*68:01 | 6% | MNG3 | MNG632 |  | 1 |  |
| Retinoblastoma-associated protein (RB1; P06400) | | | | | 52% | 33% | 33% / 39% |
| DEVKNVYF | B*18:01 | 6% | MNG6 | MNG661 |  | 13 |  |
| TEINSALVL | B*40:01; B*40:02 | 6% | MNG3 | MNG646 |  | 5 |  |
| Cytochrome c oxidase subunit 1 (MT-CO1; P00395) | | | | | 39% | 21% | 11% / 18% |
| LPVLAAGITM | B*07:02; B*35:01 | 6% | MNG628 | MNG666 |  | 3 |  |
| DNA polymerase epsilon catalytic subunit A (POLE; Q07864) | | | | | 33% | 19% | 11% / 25% |
| FYVDTVRAF | A*24:02 | 6% | MNG661 | MNG833 |  | 18 |  |
| EPSSLTYVTR | A*68:01 | 6% | MNG3 | MNG814 |  | 2 |  |
| Short transient receptor potential channel 1 (TRPC1; P48995) | | | | | 33% | 5% | 0% / 4% |
| NEIRDLLGF | B*18:01 | 6% | MNG1 | MNG628 |  | 1 |  |
| DNA mismatch repair protein Msh2 (MSH2; P43246) | | | | | 30% | 34% | 11% / 32% |
| SAAEVGFVR | A*68:01 | 6% | MNG3 | MNG814 |  | 3 |  |
| Epidermal growth factor receptor (EGFR; P00533) | | | | | 24% | 13% | 0% / 6% |
| YQDPHSTAV | C*02:02; B*39:31 | 6% | MNG702 | MNG734 |  | 28 |  |
| Adenomatous polyposis coli protein (APCP; P25054) | | | | | 24% | 18% | 0% / 19% |
| GELDTPINY | B*18:01; B*44:03 | 6% | MNG1 | MNG636 |  | 1 |  |
| LPSSSSSRGSL | B*07:02 | 6% | MNG7 | MNG814 |  | 30 |  |
| Mismatch repair endonuclease PMS2 (PMS2; P54278) | | | | | 18% | 7% | 11% / 7% |
| DALHNLFYI | B*51:01 | 6% | MNG501 | MNG833 |  | 4 |  |
| Mothers against decapentaplegic homolog 4 (SMAD4; Q13485) | | | | | 18% | 11% | 0% / 5% |
| KGFPHVIYA | C*12:03 | 6% | MNG636 | MNG661 |  | 6 |  |
| HRQGGESETF | B*38:01 B*39:31 | 6% | MNG636 | MNG734 |  | 6 |  |
| POTE ankyrin domain family member A (POTEA; Q6S8J7) | | | | | 30% | 3% | 11% / 5% |
| EVPRADLIVM | B*35:01 | 6% | MNG628 | MNG833 |  | 2 |  |
| **Meningioma-exclusive HLA class II-presented antigens derived from established TAA and CTA** | | | | | | | |
| Melanoma-associated antigen 10 (MAGEA10; P43363) | | | | |  |  |  |
| Supplementary Table 3 | |  |  |  |  |  |  |
| A/G-specific adenine DNA glycosylase (MUTYH; Q9UIF7) | | | | | 6% | 1% | 0% / 0% |
| WRRRAEDEMDLDRRAYAVWVSEVM | | 6% | MNG1 | MNG679 |  | 2 |  |
| Calcium-binding tyrosine phosphorylation-regulated protein (CABYR; O75952) | | | | | 6% | 0.5% | 0% / 0% |
| DVLMVDVATSMPVVIKEVPS | | 3% | MNG666 |  |  | 0 |  |
| PVTEGVVYIEQLPEQIV | | 3% | MNG636 |  |  | 2 |  |
| **Meningioma-exclusive HLA class II-restricted peptides derived from established TAA and CTA** | | | | | | | |
| Platelet-derived growth factor receptor-like protein (PDGFRL; Q15198) | | | | | 48% | 5% | 33% / 2% |
| APKTQSIMMQVLDKGRFQKPA | | 15% | MNG499 MNG673 MNG700 | MNG623 MNG679 |  | 5 |  |
| TQSIMMQVLDKGRFQKPA | | 15% | MNG499 MNG637 MNG700 | MNG623 MNG679 |  | 5 |  |
| TQSIMMQVLDKGRFQKP | | 15% | MNG499 MNG637 MNG679 | MNG623 MNG642 |  | 5 |  |
| IQDTWRLIHRGLGHTT | | 9% | MNG666 MNG833 | MNG702 |  | 3 |  |
| QDTWRLIHRGLGHT | | 6% | MNG666 | MNG833 |  | 2 |  |
| Alpha-1,6-mannosylglycoprotein 6-beta-N-acetylglucosaminyl-transferase A (MGAT5; Q09328) | | | | | 30% | 15% | 0% / 7% |
| DNILQRIGKLESKVDN | | 12% | MNG6 MNG642 | MNG623 MNG666 |  | 15 |  |
| LDLSKRYIKALAEENR | | 9% | MNG642 MNG679 | MNG673 |  | 13 |  |
| Cadherin-1 (CDH1; P12830) | | | | | 64% | 22% | 56% / 32% |
| GTDGVITVKRPLRFHNPQ | | 9% | MNG1 MNG702 | MNG673 |  | 3 |  |
| TGRQRTAYFSLDTRF | | 6% | MNG7 | MNG623 |  | 2 |  |
| Serine/threonine-protein kinase ATR (ATR9; Q13535) | | | | | 15% | 4% | 11% / 5% |
| GLPLSIEGHVHYLIQEATDE | | 9% | MNG501 MNG702 | MNG700 |  | 0 |  |
| Neurofibromin (NF1; P21359) | | | | | 12% | 3% | 0% / 4% |
| GNPIFYYVARRFK | | 9% | MNG6 MNG734 | MNG641 |  | 0 |  |
| Legumain (LGMN; Q99538) | | | | | 49% | 31% | 22% / 46% |
| VPKDYTGEDVTPQNFLAVLR | | 6% | MNG1 | MNG833 |  | 21 |  |
| Matrix-remodeling-associated protein 5 (MXRA5; Q9NR99) | | | | | 24% | 16% | 11% / 5% |
| DPWPRILWRLPSK | | 6% | MNG666 | MNG702 |  | 10 |  |
| GDPWPRILWRLPSK | | 6% | MNG666 | MNG702 |  | 12 |  |
| DPWPRILWRLPSKR | | 6% | MNG666 | MNG702 |  | 12 |  |
| C-1-tetrahydrofolate synthase, cytoplasmic (MTHFD1; P11586) | | | | | 6% | 15% | 0% / 18% |
| GVSGALTVLMKDAIKPNLMQ | | 6% | MNG624 | MNG682 |  | 2 |  |
| Macrophage scavenger receptor types I and II (MSR1; P21757) | | | | | 15% | 3% | 0% / 1% |
| RIQHILDMEANLMDTE | | 6% | MNG638 | MNG734 |  | 1 |  |
| Zinc phosphodiesterase ELAC protein 2 (ELAC2; Q9BQ52) | | | | | 12% | 1% | 0% / 1% |
| LSGMILTLKETGLPKCVLSGPP | | 6% | MNG7 | MNG499 |  | 0 |  |
| IIFLGTGSAIPMKIRNVSATLVNI | | 6% | MNG638 | MNG702 |  | 0 |  |
| Poly(A) polymerase alpha (PAPOLA; P51003) | | | | | 9% | 2% | 0% / 1% |
| LSVDLTYDIQSFTD | | 6% | MNG1 | MNG3 |  | 6 |  |
| Ephrin type-B receptor 2 (EPHB2; P29323) | | | | | 9% | 3% | 0% / 4% |
| TPGMKIYIDPFTYED | | 6% | MNG3 | MNG638 |  | 1 |  |
| Tyrosine-protein kinase receptor Tie-1 (TIE1; P35590) | | | | | 9% | 2% | 0% / 15% |
| QTDVIWKSNGSYFYT | | 6% | MNG499 | MNG814 |  | 0 |  |
| A/G-specific adenine DNA glycosylase (MUTYH; Q9UIF7) | | | | | 6% | 1% | 0% / 0.5% |
| WRRRAEDEMDLDRRAYAVWVSEVM | | 6% | MNG1 | MNG679 |  | 0 |  |
| ALK tyrosine kinase receptor (ALK; Q9UM73) | | | | | 6% | 1% | 0% / 1% |
| PHNEAAREILLMPTPG | | 6% | MNG7 | MNG638 |  | 0 |  |

**Supplementary Table 9** Peptide sequences and level of modulation for antigens exclusively represented by up- or down-modulated HLA class I- and II-presented peptides. UniProt accession, antigens, frequency of patients with significant modulation, peptide sequence, HLA restrictions fold change meningioma/dura [Fc(M/D)], and corrected *p*-value. Oxidized (m) and reduced (M) methionine were not treated equally in LFQ-MS and are listed separately. HLA restrictions not passing manual assessment as quality control are indicated in italic (HLA class I ligands not matching the motif of any of the patient’s HLA allotypes; HLA class II-presented proteins neither identified with peptides exceeding a length of twelve AA nor with different sequences across patients)

| **UniProt accession** | **Antigen** | **Patients with significant modulation** | **Peptide sequence** | **HLA restric-tion** | **Fc (M/D)** | **Corrected *p*-value** |
| --- | --- | --- | --- | --- | --- | --- |
| **Antigens exclusively represented by HLA class I ligands up-modulated on meningioma** | | | | | | |
| P05387 | 60S acidic ribosomal protein P2 (RLA2) | 60%  MNG679  MNG702  MNG833 | MRYVASYLL  mRYVASYLL  MRYVASYLL  mRYVASYLL  MRYVASYL  mRYVASYL  mRYVASYLL | *C*04:01*  *C*04:01*  C*06:02  C*06:02  C*06:02  C*06:02  *C*04:01* | 67.32  20.86  22.02  60.94  68.11  7.47  18.48 | 0.00093  0.00011  0.00161  0.00014  0.00005  0.00014  0.00089 |
| P0DJ07 | Protein PET100 homolog, mitochondrial (PT100) | 40%  MNG819  MNG833 | IYLTFPVAmF  IYLTFPVAmF | A*23:01  A*24:02 | 5.82  10.58 | 0.00333  0.00208 |
| P28039 | Acyloxyacyl hydrolase (AOAH) | 40%  MNG679  MNG702 | KFTNFNLFY  SVIEQLAQV | A*29:02  A*02:01 | 23.01  18.33 | 0.00002  0.00112 |
| Q14126 | Desmoglein-2 (DSG2) | 40%  MNG679  MNG819 | RFLDDLGLKF  Manual HLA annotation:  RFLDDLGLKF | *C*04:01*  A*23:01  A*23:01 | 44.61  28.79 | 0.00002  0.00176 |
| Q15796 | Mothers against deca-pentaplegic homolog 2 (SMAD2)  1 peptide multi-maps to SMAD1/3/5/9 (Q15797 / P84022 / Q99717 / O15198) | 40%  MNG702  MNG679 | LTELPPLDDY  FVKGWGAEY | A*01:01  A*29:02 | 10.83  6.52 | 0.00052  0.00049 |
| Q2TAY7 | WD40 repeat-containing protein SMU1 (SMU1) | 40%  MNG679  MNG833 | DVIRLIMQY  DVIRLImQY  DVIRLImQY | A*29:02  A*29:02  B*35:01 | 856.09  209.76  5.15 | 0.00025  0.00256  0.00037 |
| Q68CP4 | Heparan-alpha-glucosaminide N-acetyltransferase (HGNAT) | 40%  MNG679  MNG819 | NYFPFQWKL  NYFPFQWKL | A*23:01 A*23:01 | 25.65  12.97 | 0.00473  0.00090 |
| Q6KC79 | Nipped-B-like protein (NIPBL) | 40%  MNG679  MNG814 | DVIERVIQY  EVVAVDPSILAR | A*29:01  A*68:01 | 93.70  4.53 | 0.00012  0.00840 |
| Q6PI26 | Protein SHQ1 homolog (SHQ1) | 40%  MNG679  MNG833 | EAIEQILKY  NVHDImVSF | A*29:02  B*35:01 | 28.28  6.39 | 0.00002  0.00072 |
| Q6PKG0 | La-related protein 1 (LARP1) | 40%  MNG679  MNG819 | YGLEKFWAF  YGLEKFWAF | A*23:01  A*23:01 | 45.79  11.98 | 0.00012  0.00169 |
| Q96T76 | MMS19 nucleotide excision repair protein homolog (MMS19) | 40%  MNG679  MNG702 | EVVHLILFY  VDTLVTKF | A*29:02  B*37:01 | 125.12  21.09 | 0.00043  0.000009 |
| Q9BS91 | Probable UDP-sugar transporter protein SLC35A5 (S35A5) | 40%  MNG679  MNG833 | KWSIPAFLY  IFIQNSKLYF | A*29:01  A*24:02 | 17.63  10.08 | 0.000005  0.00070 |
| Q9H799 | Ciliogenesis and planar polarity effector 1 (CPLN1) | 40%  MNG679  MNG819 | KFLDLFLSY  EYIKFLDLF | A*29:01  A*23:01 | 6.92  15.21 | 0.00727  0.00279 |
| **Antigens exclusively represented by HLA class I ligands down-modulated on meningioma** | | | | | | |
| Q9Y520 | Proline-rich and coiled-coil-containing protein 2C (PRC2C) | 60%  MNG679  MNG819  MNG833 | FYmDTSHLF  FYmDTSHLF  FYmDTSHLF | A*23:01  A*23:01  A*24:02 | 0.0491  0.0957  0.1108 | 0.0000002  0.00013  0.00030 |
| Q9UL54 | Serine/threonine-protein kinase TAO2 (TAOK2)  1 peptide multi-maps to TAOK3 (Q9H2K8) | 40%  MNG679  MNG702 | SEVVAIKKm  QTELGNQLEY | B*44:03  A*01:01 | 0.1283  0.0457 | 0.00119  0.00005 |
| Q9BXC9 | Bardet-Biedl syndrome 2 protein (BBS2) | 40%  MNG679  MNG819 | AEQDLIREL  IRSNNINTL | B*44:03  C*06:02 | 0.0303  0.0793 | 0.00047  0.00205 |
| Q9BRK3 | Matrix-remodeling-associated protein 8 (MXRA8) | 40%  MNG702  MNG814 | RSEDIQLDY  ALPSRILLWK | A*01:01  A*03:01 | 0.0470  0.0240 | 0.00002  0.000002 |
| Q99733 | Nucleosome assembly protein 1-like 4 (NP1L4)  peptides multi-map to NP1L1 (P55209) | 40%  MNG679  MNG833 | EEVHDLERKY  QPmSFVLEF | B*44:03  B*35:03 | 0.0603  0.1462 | 0.00027  0.00007 |
| Q8NG06 | Tripartite motif-containing protein 58 (TRI58) | 40%  MNG702  MNG819 | GLLEGVRGV  GLLEGVRGV | A*02:01  A*02:01 | 0.0168  0.0444 | 0.000006  0.00034 |
| Q8IUQ4 | E3 ubiquitin-protein ligase SIAH1 (SIAH1) | 40%  MNG679  MNG814 | VFDTSIAQL  ATALPTGTSK | C*04:01  A*03:01 | 0.0425  0.2459 | 0.00030  0.00541 |
| Q6N022 | Teneurin-4 (TEN4) | 40%  MNG679  MNG819 | AYSDGHFLF  AYSDGHFLF | A*23:01  A*23:01 | 0.0951  0.0517 | 0.00045  0.00163 |
| Q53FT3 | Protein Hikeshi | 40%  MNG679  MNG819 | KmLDNFYNF  KmLDNFYNF | A*23:01  A*23:01 | 0.0579  0.2422 | 0.00001  0.00099 |
| Q15746 | Myosin light chain kinase, smooth muscle (MYLK) | 40%  MNG702  MNG814 | DAFEEKANI  SPQQVDFRSVL | B*51:01  B*07:02 | 0.0115  0.1016 | 0.00001  0.00024 |
| Q15005 | Signal peptidase complex subunit 2 (SPCS2) | 40%  MNG679  MNG819 | AEFTKSIAKF  REAEFTKSIA | B*44:03  B*50:01 | 0.0469  0.0947 | 0.00114  0.00709 |
| Q01459 | Di-N-acetylchitobiase (DIAC) | 40%  MNG679  MNG819 | REIEGSQVTF  SQITTVATF | B*44:03  B*15:01 | 0.0492  0.1910 | 0.000003  0.00014 |
| P57740 | Nuclear pore complex protein Nup107 (NU107) | 40%  MNG679  MNG819 | AEDELFNRY  AYLEAHETF | B*44:03  A*23:01 | 0.0275  0.1817 | 0.000001  0.00013 |
| P43243 | Matrin-3 | 40%  MNG679  MNG702 | FFGETSHNY  RTEEGPTLSY | A*29:02  A*01:01 | 0.2240  0.0696 | 0.00004  0.0000003 |
| P29966 | Myristoylated alanine-rich C-kinase substrate (MARCS) | 40%  MNG679  MNG819 | AESGAKEEL  AERPGEAAVA | B*44:03  B*50:01 | 0.0649  0.0866 | 0.00006  0.00604 |
| P24821 | Tenascin (TN-C) | 40%  MNG679  MNG819 | TYLPAPEGLKF  TYLPAPEGLKF | A*23:01  A*23:01 | 0.0468  0.0147 | 0.00029  0.00017 |
| P12755 | Ski oncogene (SKI) | 40%  MNG814  MNG819 | KPSSWLRTL  QELEFLRVA | B*07:02  B*50:01 | 0.0073  0.0619 | 0.00008  0.00006 |
| O15066 | Kinesin-like protein KIF3B (KIF3B) | 40%  MNG679  MNG819 | VYVKDLSSF  VYVKDLSSF | A*23:01  A*23:01 | 0.0474  0.1181 | 0.00005  0.00039 |
| **Antigens exclusively represented by HLA class II-restricted peptides up-modulated on meningioma** | | | | | | |
| Q6PCB0 | von Willebrand factor A domain-containing protein 1 (VQA1) | 33%  MNG700  MNG819 | ADSGYYVLELVPSAQPG  DSGYYVLELVPSAQPG  SGYYVLELVPSAQPG  ADSGYYVLELVPSAQPG  DSGYYVLELVPSAQPG  SGYYVLELVPSAQPG | Class II  Class II  Class II  Class II  Class II  Class II | 23.73  49.63  18.03  21.16  10.57  4.18 | 0.00107  0.00150  0.00140  0.00066  0.00112  0.00053 |
| O14949 | Cytochrome b-c1 complex subunit 8 (QCR8) | 33%  MNG700  MNG702 | FRVVPQFVVF  FRVVPQFVVF | *Class II*  *Class II* | 5.25  22.51 | 0.00119  0.00019 |
| **Antigens exclusively represented by HLA class II-restricted peptides down-modulated on meningioma** | | | | | | |
| Q9UBX1 | Cathepsin F (CATF) | 33%  MNG700  MNG819 | LPSNAYSAIKNLGGLE  LPSNAYSAIKNLGGLE | Class II  Class II | 0.1075  0.0996 | 0.00486  0.00112 |
| Q9GZZ6 | Neuronal acetylcholine receptor subunit alpha-10 (ACH10) | 33%  MNG700  MNG819 | YTSALRPVADTDQTLNV  YTSALRPVADTDQTLNV | Class II  Class II | 0.0148  0.0014 | 0.00733  0.00073 |
| Q96NH3 | Protein broad-minded (BROMI) | 33%  MNG700  MNG819 | TLCEKLTVSLSDPDPVF  TLCEKLTVSLSDPDPVF | Class II  Class II | 0.0075  0.0014 | 0.00722  0.00073 |
| P46459 | N-ethylmaleimide-sensitive vesicle-fusing ATPase (NSF) | 33%  MNG700  MNG819 | AAEFIQQFNNQAFS  AAEFIQQFNNQAFS | Class II  Class II | 0.0573  0.0379 | 0.00639  0.00006 |

| Source Protein | Peptide | Protein frequency in meningiomas | N° PBMC donor(s) tested | Positive primings |
| --- | --- | --- | --- | --- |
| Cathepsin K (CTSK) | LLLPVVSFA | 70% (7/10) | 2 | 2 |
| Guanylate cyclase soluble subunit alpha-3 (GUCY1A3) | SVFAGVVGV | 60% (6/10) | 1 | 1 |
| Protein Wnt-5a (WNTA) | AMSSKFFLV | 60% (6/10) | 2 | 1 |
| 1-phosphatidylinositol 4,5-bisphosphate phosphodiesterase delta-4 (PLCD4) | ALSSLVIYL | 30% (3/10) | 1 | 0 |
|  | ILFKDVVATV |  | 1 | 0 |
| Sestrin-3 (SESN3) | SLPELVHAV | 70% (7/10) | 1 | 0 |
| Melanoma associated antigen D2 (MAGED2) | YSLEKVFGI | 70% (7/10) | 2 | 0 |
| Solute carrier family 25 member 44 (SLC25A44) | SLVAQSITV | 40% (4/10) | 1 | 0 |
| Desmoplakin (DSP) | SMVEDITGLRL | 60% (6/10) | 1 | 0 |

**Supplementary Table 10** Summary of the *in vitro* priming assessment of the 9 top-ranking HLA-A*02:01 restricted peptides. PBMC deriving from buffy coats of healthy donors were used and the number of healthy donors used for each peptide is depicted in the column N° PBMC donor(s) tested.
