# Supplementary material for: A T-cell antigen atlas for meningioma: novel options for immunotherapy
Source: Acta Neuropathol. 2023 Jun 27;146(2):173–90. doi: 10.1007/s00401-023-02605-w (PMC10329067; doi:10.1007/s00401-023-02605-w)
Supplement: Supplementary file 2 — Supplementary file2 (DOCX 2970 kb) [file 401_2023_2605_MOESM2_ESM.docx]

**
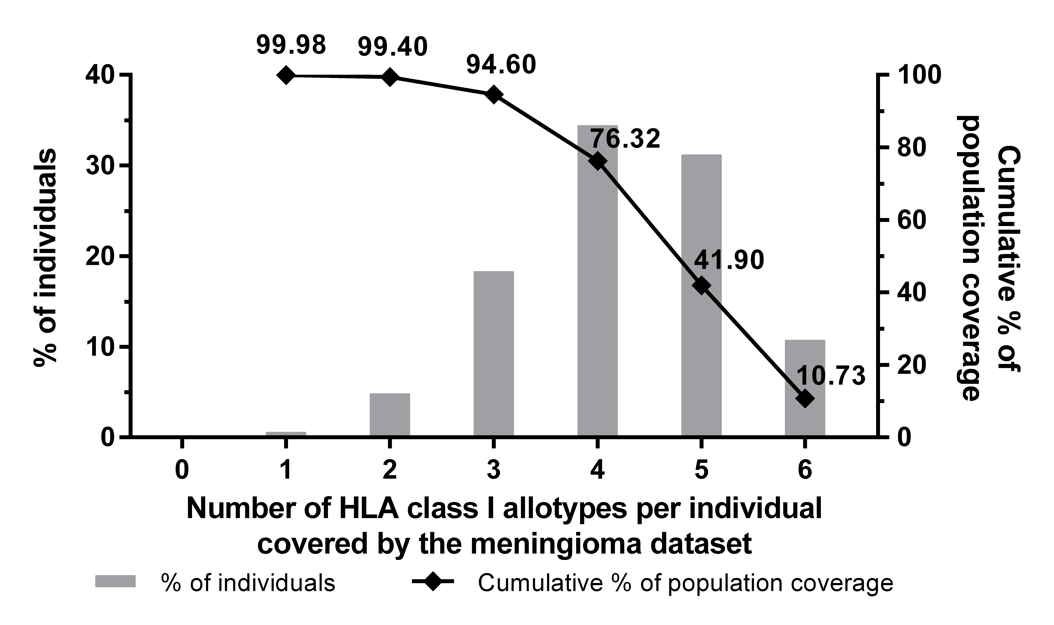
**

**Supplementary Fig. 1** HLA class I allotype population coverage. Using the population coverage tool provided by the IEDB Analysis Resource ([www.iedb.org](http://www.iedb.org)), the world population coverage of the 58 distinct HLA A, -B, and -C allotypes was calculated. The bar charts associated with the left on y-axis indicate the percentage of individuals positive for a specific number of HLA class I allotypes (max. of 6). The cumulative percentage of population coverage is displayed with the line diagram (associated with the right y axis). As a result, the HLA class I allotypes of the meningioma cohort cover 99.98% of the world population whereas 94.60% of all individuals are positive for at least three HLA class I allotypes


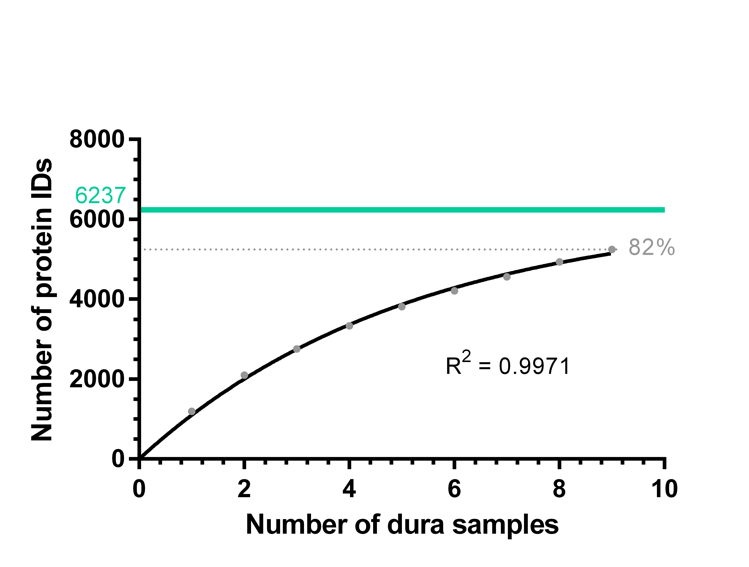

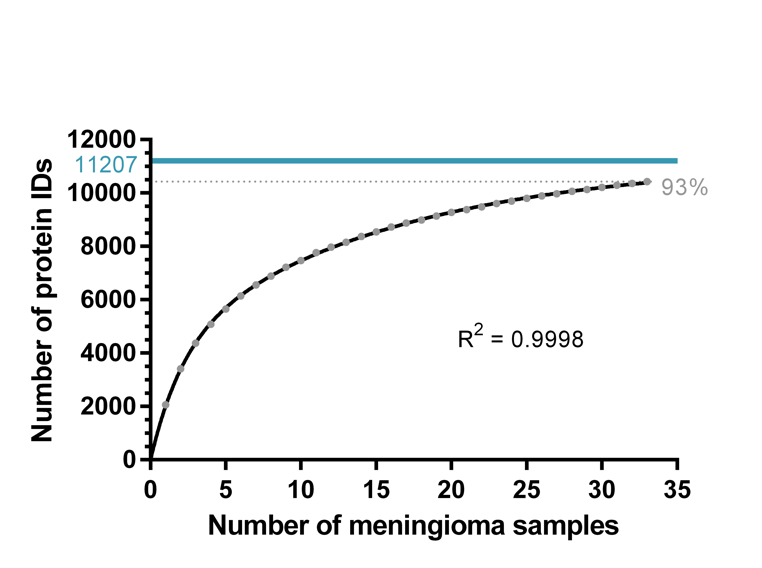


**Supplementary Fig. 2** Saturation analysis for the identification of antigens represented by HLA class I ligands on meningioma (left panel) and tumor-free dura tissue (right panel). For each source count, the mean number of antigens was calculated by 1,000 random samplings. Using non-linear regression, exponential functions with a forced y-intercept of 0 were fitted. For both models, the goodness of fit was in the uppermost range (R^2^ = 0.9998 and R^2^ = 0.9971). Based on these curves, the maximum attainable number of distinct source proteins was estimated (highlighted as solid lines)


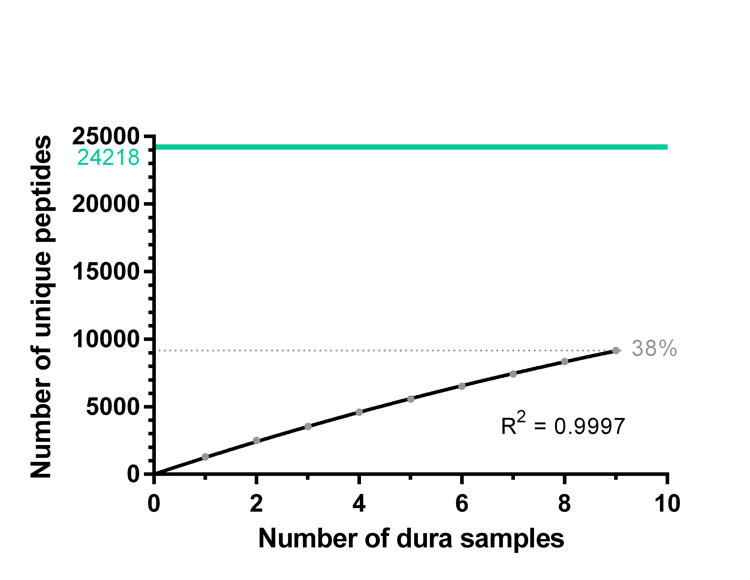

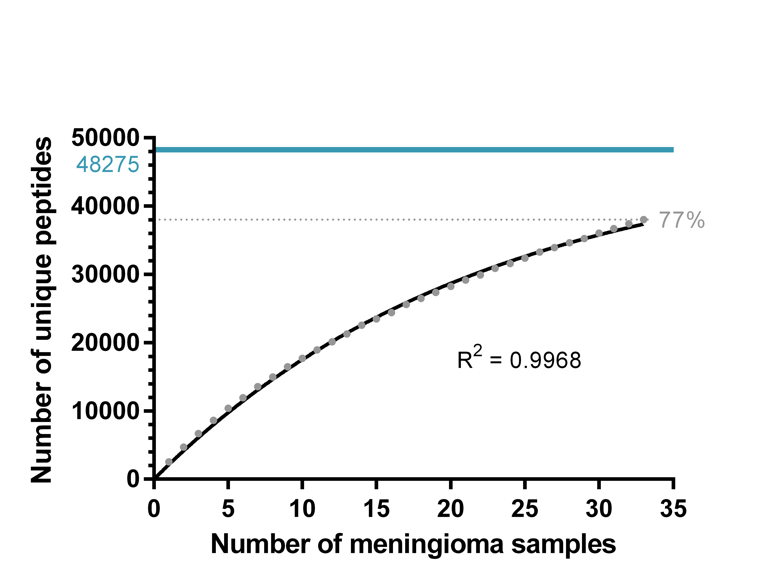


**Supplementary Fig. 3** Saturation analysis for the identification of HLA class I ligands in meningioma (left panel) or tumor-free dura tissue (right panel). For each source count, the mean number of peptides was calculated following the calculation of Supplementary Fig.2. The goodness of fit resulted R^2^ = 0.9968 and R^2^ = 0.9997 for meningioma and tumor-free dura tissue, respectively. Based on these curves, the maximum attainable number of distinct peptides was estimated (highlighted as solid lines)


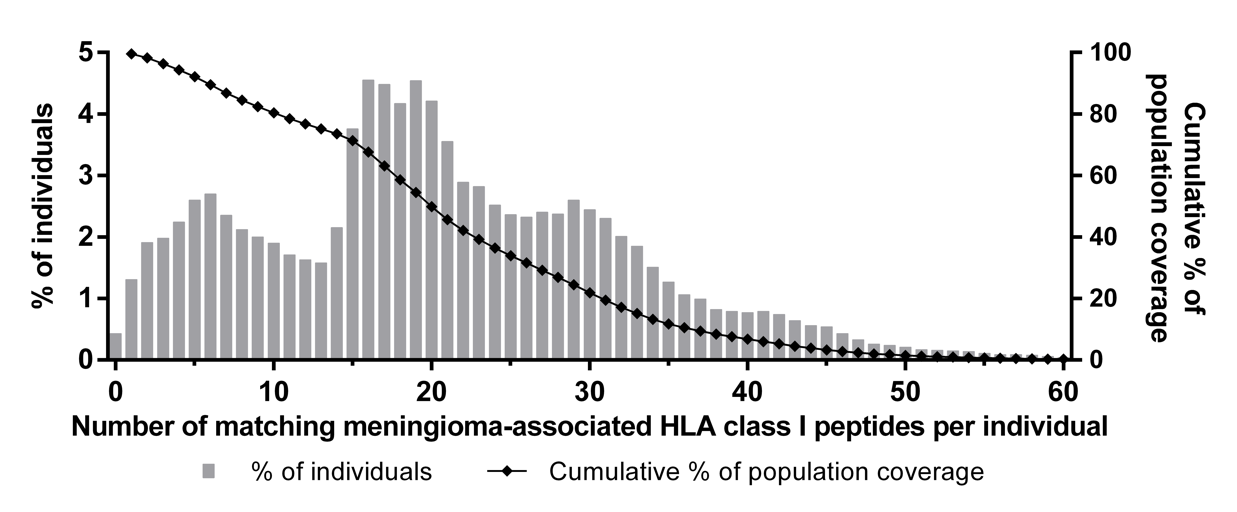


**Supplementary Fig. 4** Population coverage of meningioma-associated HLA-A, -B, and -C ligands. The percentage of individuals with a specific number of matching peptides (max. of 74) is indicated by the bar charts (associated with the left Y-axis) and was calculated by using the population coverage tool provided by the IEDB Analysis Resource ([www.iedb.org](http://www.iedb.org)), The line diagram (associated with the right Y-axis) shows the cumulative percentage of population coverage. The candidate target peptides cover 99.57% of the world population meaning that only 0.43% of all individuals are negative for all HLA class I allotypes for which meningioma-associated peptides were defined. On average, 21 peptides are expected to match per patient worldwide

**Supplementary Fig. 5** Population coverage of meningioma-associated HLA class I peptides. The population coverage of the n=141 candidate target peptides was calculated on a per-country basis exploiting the coverage tool provided by the IEDB Analysis Resource ([www.iedb.org](http://www.iedb.org)). On average, 21 HLA class I peptides match per patient worldwide. Countries not displayed in the IEDB tool or not covered by the geographic heat map add-on of Microsoft Excel are colorless. For visualization of the United Kingdom, the individual values of England, Northern Ireland, Scotland, and Wales were multiplied with the relative area portion


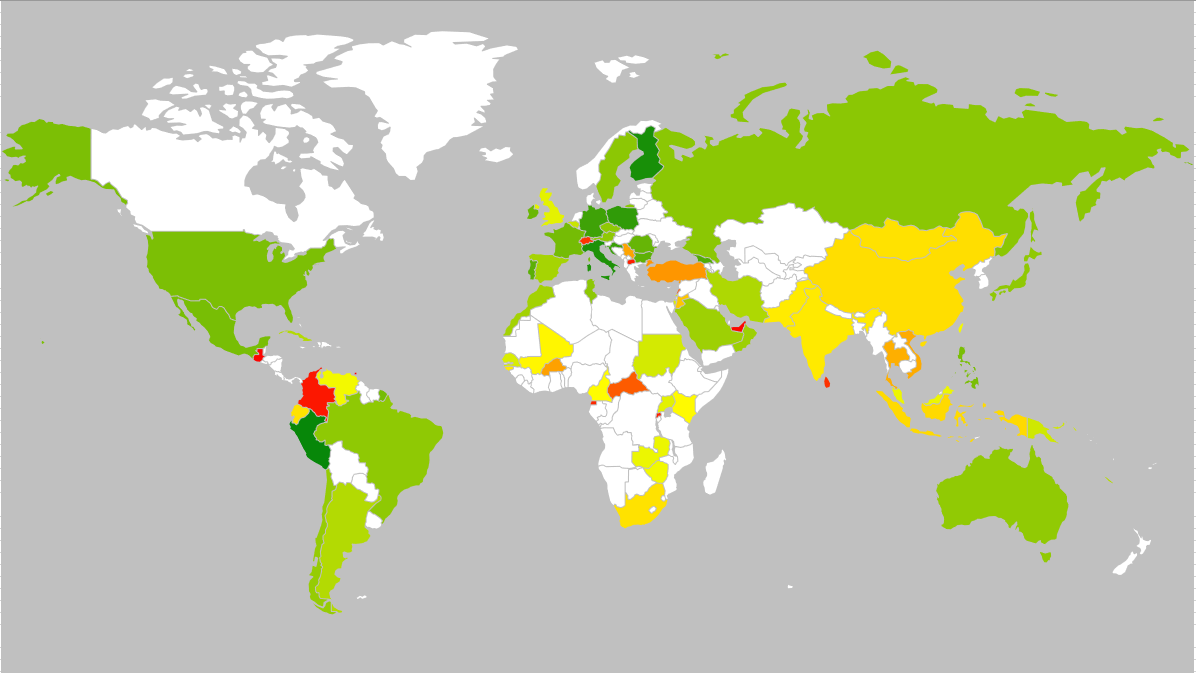




**mean number of HLA class I peptides per patient**

0 15 30


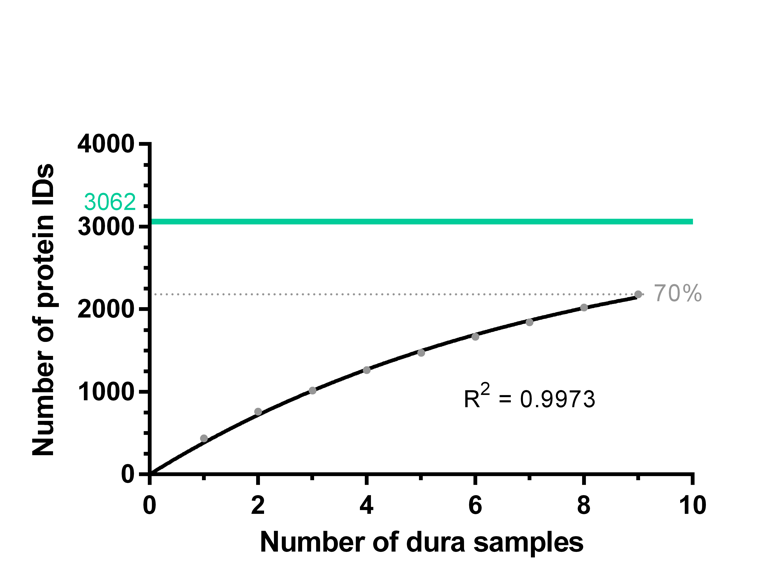

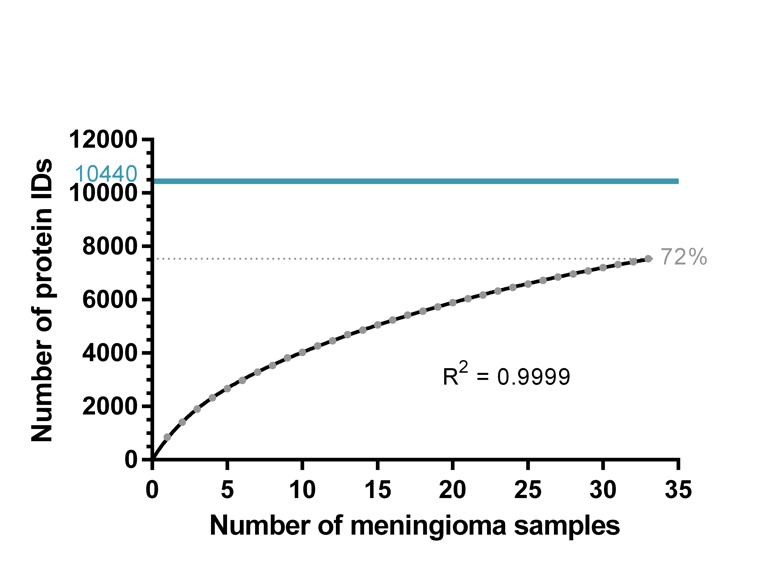


**Supplementary Fig. 6** Saturation analysis for the identification of antigens represented by HLA class II peptides on meningioma or tumor-free dura tissue. For each source count, the mean number of antigens was calculated as for Supplementary Fig. 2. The goodness of fit was in the uppermost range (R^2^ = 0.9999 and R^2^ = 0.9973). Based on these curves, the maximum attainable number of distinct source proteins was estimated (highlighted as solid lines)


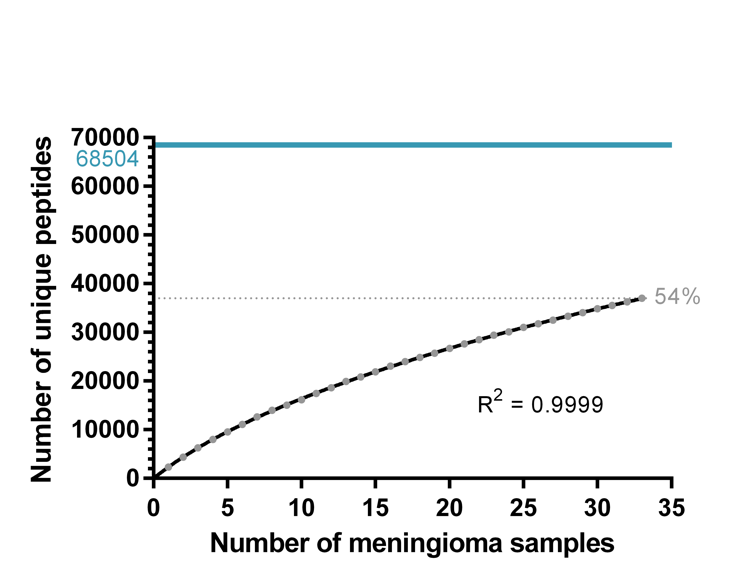

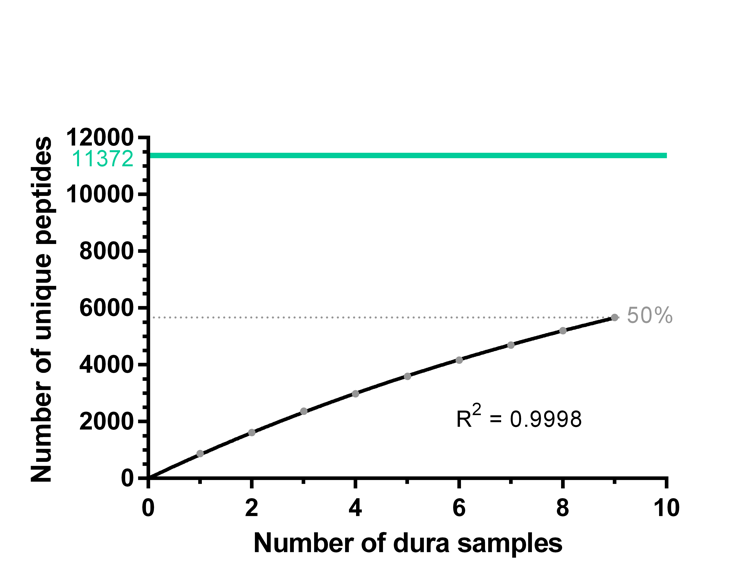
**Supplementary Fig. 7** Saturation analysis for the identification of HLA class II-presented peptides in meningioma (left panel) or tumor-free dura tissue (right panel). For each source count, the mean number of peptides was calculated following the calculation of Supplementary Fig. 2. For both models, the goodness of fit was in the uppermost range (R^2^ = 0.9999 and R^2^ = 0.9998). Based on these curves, the maximum attainable number of distinct peptides was estimated (highlighted as solid lines)


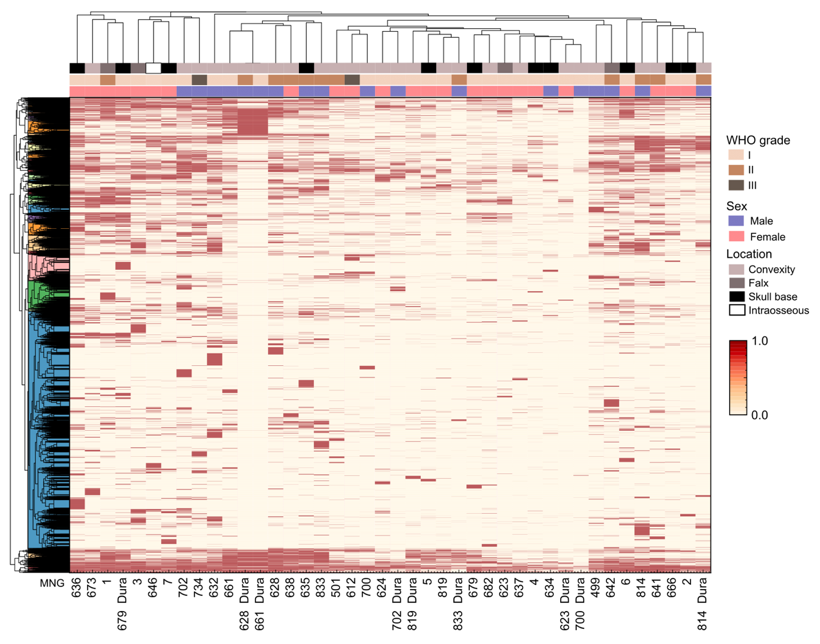


**HLA class I**

**HLA class II**


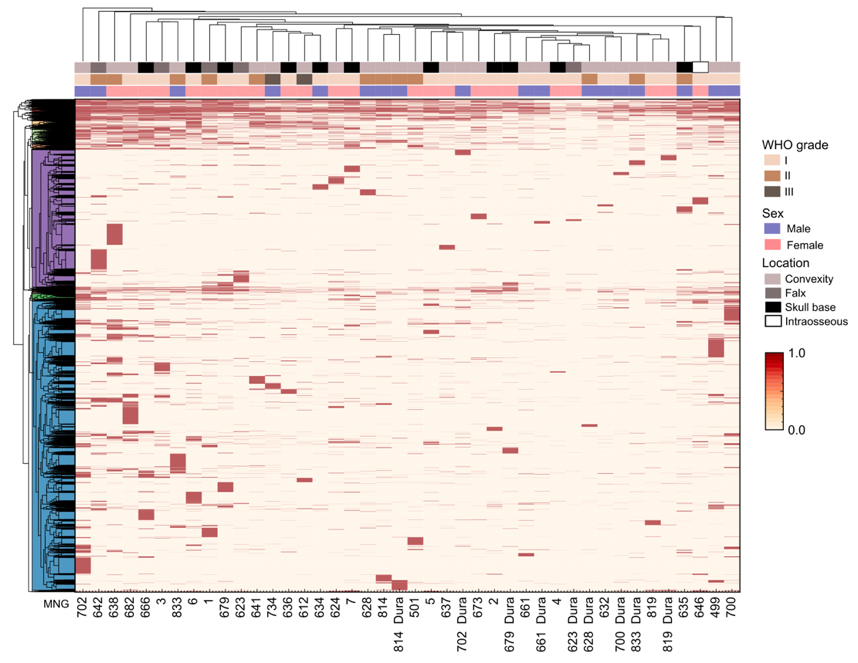


**Supplementary Fig. 8** Heatmaps depicting unsupervised hierarchical clustering of HLA class I (left panel) and HLA class II (right panel) source proteins. WHO grade, sex and location of the tumor were considered for each patient. In the HLA class I hierarchical clustering, 10450 source proteins are considered whereas the HLA class II clustering depicts 7251 source proteins
